# Biotransformation of Xanthohumol by Entomopathogenic Filamentous Fungi

**DOI:** 10.3390/ijms251910433

**Published:** 2024-09-27

**Authors:** Daniel Łój, Tomasz Janeczko, Agnieszka Bartmańska, Ewa Huszcza, Tomasz Tronina

**Affiliations:** Department of Food Chemistry and Biocatalysis, Wrocław University of Environmental and Life Sciences, Norwida 25, 50-375 Wroclaw, Poland; danloj.210@gmail.com (D.Ł.); agnieszka.bartmanska@upwr.edu.pl (A.B.); ewa.huszcza@upwr.edu.pl (E.H.)

**Keywords:** xanthohumol, biotransformation, *O*-glycosylation, entomopathogenic fungi, chalcone isomerase

## Abstract

Xanthohumol (**1**) is a major prenylated flavonoid in hops (*Humulus lupulus* L.) which exhibits a broad spectrum of health-promoting and therapeutic activities, including anti-inflammatory, antioxidant, antimicrobial, and anticancer effects. However, due to its lipophilic nature, it is poorly soluble in water and barely absorbed from the gastrointestinal tract, which greatly limits its therapeutic potential. One method of increasing the solubility of active compounds is their conjugation to polar molecules, such as sugars. Sugar moiety introduced into the flavonoid molecule significantly increases polarity, which results in better water solubility and often leads to greater bioavailability. Entomopathogenic fungi are well known for their ability to catalyze *O*-glycosylation reactions. Therefore, we investigated the ability of selected entomopathogenic filamentous fungi to biotransform xanthohumol (**1**). As a result of the experiments, one aglycone (**2**) and five glycosides (**3**–**7**) were obtained. The obtained (2″*E*)-4″-hydroxyxanthohumol 4′-*O*-β-D-(4‴-*O*-methyl)-glucopyranoside (**5**) has never been described in the literature so far. Interestingly, in addition to the expected glycosylation reactions, the tested fungi also catalyzed chalcone–flavanone cyclization reactions, which demonstrates chalcone isomerase-like activity, an enzyme typically found in plants. All these findings undoubtedly indicate that entomopathogenic filamentous fungi are still an underexploited pool of novel enzymes.

## 1. Introduction

Xanthohumol (**1**) is a major prenylated flavonoid isolated from hops (*Humulus lupulus* L.), and is known for its broad spectrum of biological activities, including anti-inflammatory, antioxidant, antibacterial, antifungal, anti-plasmodial, antiviral, and anticancer effects, as well as its potential in the prevention of metabolic disorders [1,2,3,4,5]. The potent and diverse biological activity of xanthohumol (**1**) is attributed to the presence of a prenyl group in its structure. The prenylation of flavonoids is known to enhance their antibacterial, anti-inflammatory, antioxidant, cytotoxic, insecticidal, and estrogenic activities [6]. The prenyl side chain may increase the binding affinity of flavonoids to P-glycoprotein, which results in a significant improvement in biological activity [7], including anticancer properties [8]. Furthermore, in many cases, prenylated flavonoids act selectively, showing a higher cytotoxic effect against cancer cells than against normal cells [8]. However the presence of a non-polar prenyl group in the flavonoid molecule has the adverse effect of reducing their water solubility, which in turn negatively affects their bioavailability and reduces their absorption [6], consequently limiting the therapeutic potential of these valuable biologically active compounds. Pharmacokinetic studies of xanthohumol (**1**) show that this compound is very poorly absorbed from the gastrointestinal tract. The bioavailability of this chalcone after administration to rats at doses of 40, 100, and 200 mg/kg body weight was 1.16%, 0.96%, and 0.53%, respectively [9], which may be the result of significantly limited absorption in the small intestine and the rapid metabolism of xanthohumol (**1**) by microorganisms in the colon [1].

One of the strategies to improve the pharmacokinetic properties of bioactive lipophilic compounds such as flavonoids is focused on structural modifications, including glycosylation, which emerges as a promising approach to enhance solubility and bioavailability. The introduction of sugar residues to flavonoids significantly increases their hydrophilicity, making them more soluble in water and, consequently, more bioavailable. This modification can also stabilize flavonoids against oxidative degradation and enzymatic hydrolysis, and prolong their half-life in vivo [10,11]. Quercetin, a well-known flavonoid with potent antioxidant properties, has limited solubility in water and low oral bioavailability. However, when glycosylated to isoquercetin (quercetin 3-*O*-glucoside), its water solubility is significantly enhanced, leading to nearly 500% higher absorption compared to quercetin [12]. In addition, the presence of a sugar molecule can also increase the biological activity of flavonoids. For example, isoquercetin inhibits influenza A and B viruses (IAV, IBV) 40 times more potently than quercetin and, in combination with antiviral drugs, reduces the virus’ drug resistance [13,14].

The chemical introduction of sugar moieties into flavonoids is difficult and very often involves the attachment of a sugar moiety before the formation of the flavonoid backbone. The final stage is the unblocking of hydroxyl groups by removing the protective groups in the sugar molecule, which may lead to the degradation of the synthesized glycoside. Due to the different reactivity of hydroxyl groups related to their specific position in the molecule, the presence of steric hindrances, and the possibility of creating intramolecular hydrogen bonds, there is no single strategy for introducing sugar residues into flavonoids by chemical methods [15]. In living organisms, on the other hand, through glycosyltransferase (GT) enzymes, glycosylation is a natural process and is the predominant strategy used to regulate the physicochemical and biological properties of biomolecules [16,17,18]. Therefore, it seems natural to use biotechnological methods, including the whole-cell biotransformation process of wild strains of microorganisms which has the ability to catalyze regioselective glycosylation reactions to obtain glycosylated derivatives of bioactive compounds.

Among the microorganisms that have been used to perform glycosylation reactions due to their ability to produce a wide variety of glycosyltransferases, entomopathogenic fungi, in particular, have shown remarkable potential for glycosylation of biologically active compounds, including flavonoids. Our previous studies concerning the biotransformation of flavonoids have shown that entomopathogenic filamentous fungi *Beauveria bassiana* AM278 is an effective regioselective biocatalyst for glycosylation reactions where 4-O-methylglucose is conjugated to flavonoid molecules. In contrast, non-entomopathogenic fungi belonging to *Absida* or *Rhizopus* genera introduced a glucose moiety [19,20]. However, a study conducted on a larger panel of entomopathogenic fungi showed that although they preferentially attach the 4-*O*-methyl derivative of glucose, they also catalyze the *O*-glycosylation reaction by attaching a glucose moiety and gentiobiose—a disaccharide composed of two units of D-glucose [21,22]. This phenomenon shows how rich in glycosyltransferases these fungi can be and prompted us to re-examine the microbial *O*-glycosylation process of xanthohumol (**1**), focusing exclusively on the use of entomopathogenic fungi as biocatalysts.

In addition to the expected known derivatives, including glucosides and methylglucosides of xanthohumol, a new, previously unpublished glycosylated derivative of xanthohumol was obtained. Interestingly, isoxanthohumol (**2**) and its glycoside structure were obtained. The chalcone–flavanone isomerization reaction observed in some of the fungi tested demonstrates that they are also rich in chalcone isomerases. All these findings undoubtedly indicate that entomopathogenic filamentous fungi are still an underexploited repository of novel enzymes.

## 2. Results and Discussion

Our previously described results on the biotransformation of prenylated hop flavonoids such as xanthohumol (**1**) and isoxanthohumol (**2**) using cultures of entomopathogenic filamentous fungi involved two strains from the same species, *Beauveria bassiana*. Both *B. bassiana* AM278 and *B. bassiana* AM446 were found to be capable of catalyzing regioselective *O*-glycosylation, in which the only biotransformation product obtained was 4-*O*-methylglucosides of the aforementioned flavonoids [19,23]. Among entomopathogenic filamentous fungi, *B. bassiana* strains are most commonly used in the biotransformation of flavonoids [22,24,25,26,27,28,29].

**Figure 1:**
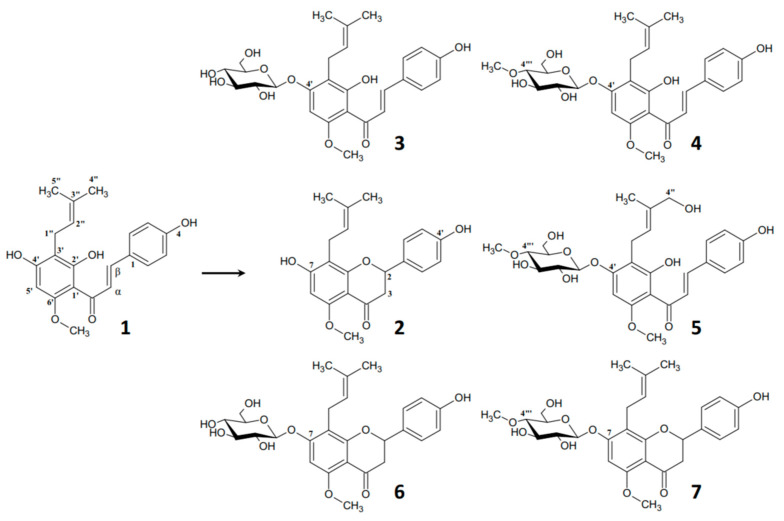
Progression of biotransformation of xanthohumol (**1**) to metabolites **2**–**7** by selected fungi.

To expand the pool of biocatalysts for the study of the glycosylation of xanthohumol (**1**), four strains of the genus *Beauveria*, *B. bassiana* KCh BBT, *B. bassiana* KCh J 1.5, *B. caledonica* KCh J3.3, and *B. feline* ENC3; three strains of the genus *Isaria*, *I. farinosa* KCh KW1.1, *I. fumosorosea* KCh J2, and *I. tenuipes* MU35; and one strain of *Metarhizium robertsii* MU4 were selected. These fungi are known for their efficient biotransformation of flavonoid compounds [30]. Among the selected strains, *I. fumosorosea* KCh J2 showed the ability to glycosylate 3-hydroxyflavone, forming three glycosylated derivatives simultaneously by attaching glucose, 4-*O*-methylglucose, and gentiobiose at the same substrate position, respectively. The same strain also showed the ability to hydroxylate flavone at the 2′ and 4′ positions and then *O*-glycosylate at these positions to form 2′- and 4′-*O*-β-D-(4″-*O*-methyl)-glucosides, respectively [31]. This demonstrates that *I. fumosorosea* KCh J2 is able to introduce various sugar molecules at multiple positions. The selection of microorganisms capable of attaching different sugar units to hydroxyl groups located in different positions of the flavonoid molecule allows the study of the glycosylation process, including its regioselectivity. Since the presence of a sugar moiety in the flavonoid molecule was proposed to be the crucial determinant of its absorption in humans [32] xanthohumol (**1**), due to its extremely low bioavailability [9], appears to be an ideal candidate to study the incorporation of sugar molecules into its structure in order to improve its water solubility and consequently its bioavailability. Herein, the aim of the study was to obtain a number of glycosylated derivatives of xanthohumol via biotransformation by selected entomopathogenic filamentous fungi. The biotransformation of xanthohumol (**1**) by tested strains yielded six products (**2**–**6**) (Figure 1).

Among the tested fungal strains, all except *B. feline* ENC3 showed very high or high xanthohumol (**1**) biotransformation capacity (Table 1). (Numeric values of conversion rate xanthohumol (**1**) and its metabolites after the 1st, 3rd, 7th, and 10th days of biotransformation are available in the Supplementary Materials—Table S1.).

Among the isolated products, three were xanthohumol glycosides **3**–**5**, along with isoxanthohumol (**2**) and two of its glycosides **6** and **7** (Figure 1). Xanthohumol 4′-*O*-β-D-glucopyranoside (**3**) was previously obtained as a major product in cultures of the fungi *Absidia glauca* AM177, *A. caerulea* AM93, *Rhizopus nigricans* UPF701, *Cunninghamella elegans* var. *elegans* 6992, and *Penicillium chrysogenum* 6933 [19,33], whereas xanthohumol 4′-*O*-β-D-(4‴-*O*-methyl)-glucopyranoside (**4**) was obtained in a culture of *B. bassiana* AM278 and *B. bassiana* AM446 [19,23]. Metabolite **2**-isoxanthohumol is a naturally occurring flavanone in hops and the main prenylated flavonoid of beer [34,35,36]. The presence of both isoxanthohumol (**2**) and its glycosides (**6** and **7**) proves the activity of the chalcone isomerase enzyme in entomopathogenic filamentous fungi. Chalcone isomerase is a key enzyme in the biosynthesis of flavonoids in plants. It catalyzes the enantioselective isomerization of 2′-OH chalcone to flavanone. Although this enzyme is found mainly in plants, it has been identified in bacteria [37] and fungi [38]. Xanthohumol (**1**) biotransformations carried out in filamentous fungal cultures in which isoxanthohumol (**2**) or its derivatives were isolated from the biotransformation products are known. Biotransformations of chalcone **1** by *Rhizopus oryzae* KCTC 6946 resulted in obtaining flavanone **2**-isoxanthohumol as the main biotransformation product [33], while *Aspergillus ochraceus* AM456 produced a dihydrofuran derivative of isoxanthohumol as a minor product [39]. The biotransformation of xanthohumol (**1**) in a *Mortierella mutabilis* AM404 culture led to the production of isoxanthohumol glucoside (**6**) [19]; this product was also observed in the aforementioned biotransformations carried out by *C. elegans* var. *elegans* 6992 and *P. chrysogenum* 6933 as minor metabolites [33]. Although there are known examples of the microbiological isomerization of chalcones to flavanones and their derivatives, to the best of our knowledge, this kind of reaction in the case of the biotransformation of xanthohumol (**1**) performed by entomopathogenic filamentous fungi has not been reported so far. Despite glycoside **6** being obtained in a small yield in the biotransformation of xanthohumol (**1**), the major way of producing it is through the biotransformation of isoxanthohumol (**2**) as a substrate, which was described in our previous work [20]. Isoxanthohumol 7-*O*-β-D-(4‴-*O*-methyl)-glucopyranoside (**7**) is known and it was obtained as a major product of the biotransformation of isoxanthohumol (**2**) by *B. bassiana* AM278 [20]. Among the obtained xanthohumol metabolites, compound **5** identified as (2″*E*)-4″-hydroxyxanthohumol 4′-*O*-β-D-(4‴-*O*-methyl)-glucopyranoside is novel and has never been described in the literature. The structure of metabolite **5** was fully confirmed using Nuclear Magnetic Resonance (NMR) spectroscopy, including (^1^H NMR, ^13^C NMR, DEPT 135, ^1^H-^1^H NMR (COSY), ^1^H-^13^C NMR (HSQC), ^1^H-^13^C NMR (HMBC)).

### Identification of (2″E)-4″-Hydroxyxanthohumol 4′-O-β-D-(4‴-O-Methyl)-Glucopyranoside (***5***)

The recorded absorption spectrum and the absorption maximum value of metabolite **5** (λ_max_ = 369.4 nm) (Figure S1—Supplementary Materials) confirmed that the obtained compound as well as xanthohumol (**1**) belongs to the same class of flavonoids—chalcones. A much higher polarity of metabolite **5** compared to substrate **1** observed in UHPLC chromatograms (Rt_5_ = 3.19 min. vs. Rt_1_ = 4.42) (Figure S2—Supplementary Materials) strongly suggested the attachment of a sugar molecule. However, the different retention times of metabolite **5** compared to previously obtained xanthohumol glycosides 4′-*O*-glucoside (**3**) and 4′-*O*-(4‴-*O*-methyl) glucoside (**4**) in UHPLC analysis proved that another additional structural modification beside glycosylation is also present.

**Figure 2:**
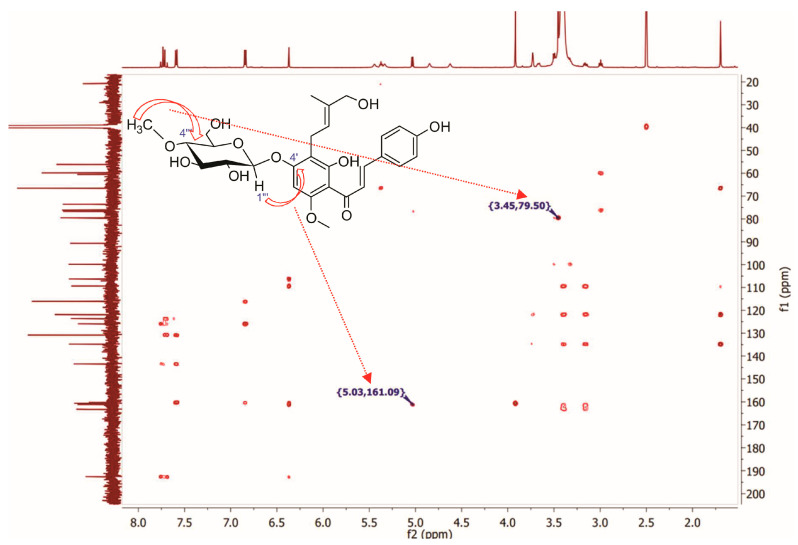
Key HMBC correlations for the structure elucidation of metabolite **5**. Open arrows represent correlations between atoms, dashed arrows represent cross-peak positions.

Recorded NMR spectra of metabolite **5** showed remarkable differences compared to substrate **1**. Apart from six additional signals (which are not present in the xanthohumol (**1**) spectrum) in the δC 60–100 ppm range in the ^13^C NMR spectrum of metabolite 5 (Figure S4—Supplementary Materials), which are coupled with signals in the δH 2.90–5.10 region in 1H NMR (confirmed by HSQC spectrum—Figure S7—Supplementary Materials) which confirms the presence of hexose moiety, there is one more additional signal of carbon atom present at δC 59.8, which correlates with a three-proton singlet present at δH 3.45 (Figures S7 and S8—Supplementary Materials). The value of chemical shift (3.45 ppm), multiplicity (singlet), and integration (3H) proves that it comes from the *O*-methyl group (-O-CH_3_). The position of this methoxy group was confirmed by HMBC spectra in which the singlet present at δH 3.45 gives long-distance correlation with the signal of a carbon atom at δC 79.5 (C-4″) (Figure S9—Supplementary Materials). Precise determination of the position of all hydrogen atoms belonging to the sugar moiety, despite the presence of a broad signal from water in the δH 3.30–3.50 range that covers most of them (an impurity present in the DMSO-d_6_ used in the analysis), was made possible by correlations of particular carbon atoms with particular hydrogen atoms of the sugar moiety in HSQC spectra. The recorded spectral data prove beyond any doubt that the conjugated sugar moiety is *β*-D-(4-*O*-methyl)-glucopyranose. The recorded HMBC spectrum was also useful to determine the position of attaching the sugar moiety. The correlation between H-1‴ (δH 5.03) and C-4′ (δC 161.1) proves that the glucose derivative is bound to the carbon atom C-4′ (Figure 2). The spectral data proved the attachment of *β*-D-(4-*O*-methyl)-glucopyranose to the hydroxyl group present at C-4′ in xanthohumol (**1**) and they are consistent with the data we published previously [19,23].

In addition to the presence of additional signals confirming the *O*-glycosylation reaction by incorporating 4-*O*-methyl-glucose at the C4′ position, significant differences in prenyl group signals were also observed. The ^1^H NMR spectrum of xanthohumol (**1**) shows two three-proton singlets present at δH 1.60 and 1.69, which originate from the hydrogen atoms of the terminal C4″ and C5″ methyl groups of the prenyl group, while only one three-proton singlet in this chemical shift range (1.70 ppm) is present in the ^1^H NMR spectrum of metabolite **5** (Figure S3, Supplementary Materials). The same trend is also shown in ^13^C NMR spectra, where there are two signals present at δC 17.69 and 25.51 which correspond to C4″ and C5″ in the spectrum of xanthohumol (**1**), whereas there is only one carbon atom signal shifted to 13.71 ppm in the ^13^C NMR spectrum of metabolite **5** (Figure S4, Supplementary Materials). These data strongly suggest a structural modification in one of the terminal methyl groups in the prenyl moiety. The missing carbon atom signal of C5” is significantly low field shifted to δC 66.5 and gives correlation with a two-proton singlet present at 3.73 ppm in the HSQC spectrum of metabolite **5**. Furthermore, the DEPT 135 spectrum confirms that the signal came from a methylene group instead of the methyl group (Figure S5, Supplementary Materials). Chemical shifts (^1^H NMR and ^13^C NMR spectra) and integration and multiplicity (^1^H NMR spectrum) prove that the terminal methyl group (-CH_3_) was hydroxylated (to -CH_2_-OH). However, an important issue of this modification is to confirm which exactly methyl group was oxidized, and whether it was the *cis* or *trans* terminal methyl group.

**Figure 3:**
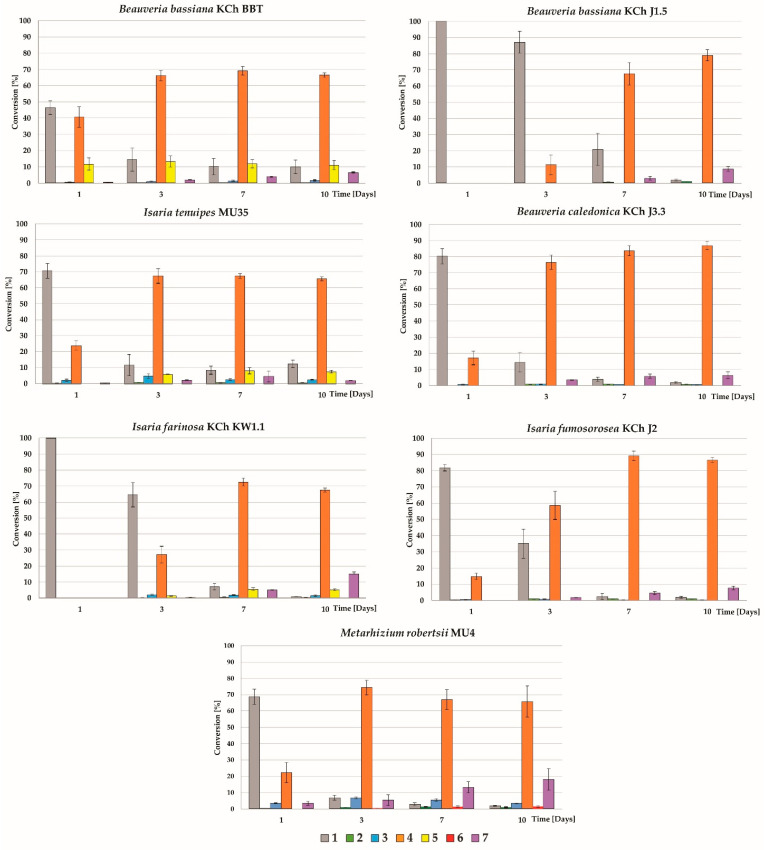
Progression of xanthohumol (**1**) biotransformation by selected fungi to metabolites **2**–**7**.

Tueting et al. [40], as well as Mao et al. [41], proved that for compounds that have a prenyl group conjugated to an aryl group (benzene or its derivatives), hydroxylation of the terminal methyl group of the prenyl group in the *cis* position causes significant changes in the multiplicity of the signal originating from the hydrogen atoms of the *trans* methyl group. It changes from a singlet to a quartet; moreover, the carbon atom signal from the hydroxylated *cis* methylene shows a chemical shift below 62 ppm. In contrast, hydroxylation of the *trans* methyl group does not change the multiplicity of the signal originating from the hydrogen atoms of the *cis* methyl group (which remains as singlet) and the signal from the hydroxylated *trans* methylene group is low field shifted to values much greater than 62 ppm. The signal of the hydrogen atoms of the terminal methyl group of the prenyl moiety in metabolite **5** is present as a singlet of three protons; additionally, the signal of the carbon atom of the newly hydroxylated group (-CH_2_-OH) in the ^13^C NMR spectrum is present at a chemical shift of 66.5 ppm, which confirms beyond any doubt that the *trans* methyl group of the prenyl moiety was hydroxylated, but the *cis* group was not. Recorded NMR spectra confirmed that metabolite **5** is a product of *trans* hydroxylation of the methyl group of the prenyl moiety and an *O*-glycosylation reaction at the C4′ position, where the conjugated sugar is a glucose derivative (4-*O*-methyl-glucopyranose). High-resolution electrospray ionization mass spectral data (Figure S10 in Supplementary Materials) showed the presence of the molecular ion [M-H]^−^ at *m*/*z* 545.2027 which confirmed the molecular formula of C_28_H_34_O_11_ (calcd. for C_28_H_34_O_11_-H, 354.2028; mass error = −0.18 ppm). ATR-IR: 3497 (C2′-OH intramolecular hydrogen bond to C=O), 3227 (C4-OH), 2919 (C-H), 2889 (C-H), 2850 (C-H), 1608 (C=O), 1590 (C=C), 1557 (C=C), 1510 (-CH_3_), 1413 (-CH_3_), 1103 (C-O), 1032 (C-O) cm^−1^ (Figure S12 in Supplementary Materials). All recorded NMR, HR MS, HR MS-MS, and ATR-IR spectra are available in the Supplementary Materials (Figures S3–S12). The progress of xanthohumol (**1**) biotransformation by particular strains of entomopathogenic fungi is shown in Figure 3.

Except for *B. feline* ENC3, all the tested microorganisms were capable of biotransforming xanthohumol (**1**). The main biotransformation product was xanthohumol 4′-*O*-β-D-(4‴-*O*-methyl)-glucopyranoside (**4**), the amount of which after 10 days of the process ranged from 65% to nearly 87%. The most efficient producers of metabolite **4** were *B. caledonica* KCh J3.3 and *I. fumosorosea* KCh J2 strains (86.4 ± 1.6% and 86.8 ± 2.5 after 10 days of biotransformation, respectively (*p* value > 0.05) (Supplementary Materials—Table S1)). *B. bassiana* KCh BBT, compared to the other strains tested, transformed substrate **1** significantly faster (*p* value < 0.05)) at the beginning of the process (46.35 ± 4.2% of the substrate left after 1 day of the biotransformation process). This strain also showed a different metabolism of xanthohumol (**1**) compared to the other fungi of the genus *Beauveria*, since it produced a metabolite **5** similar to *I. tenuipes* MU35 and *I. farinosa* KCh KW1.1 metabolite **5**. *B. bassiana* KCh BBT and *I. tenuipes* MU35 showed a similar tendency in the biotransformation of xanthohumol (**1**). After 3 days of the process, no difference in the production of metabolite **4** (*p* value > 0.05) and no increase in the formation of metabolite **5** were observed. The amount of compound **5**, although different for each strain (*p* value < 0.05), remained unchanged until the end of the process. The biotransformation of xanthohumol (**1**) by these cultures significantly slowed down after the third day of the process. The amount of substrate **1** remained at approximately the same level until the end of the process, while the other tested microorganisms transformed xanthohumol (**1**) almost completely (Figure 3). The lack of further conversion of xanthohumol (**1**) immediately following the third day of biotransformation in the presence of product **5** may suggest that it exhibits antifungal activity against tested fungi.

All organisms capable of transforming xanthohumol (**1**) also showed the ability to undergo chalcone–flavanone isomerization, which is particularly evident in the production of metabolite **7**, the cyclization product of metabolite **4**. The most effective producers of metabolite **7** were *M. robertsii* MU4 and *I. farinosa* KCh KW1.1 which accumulated 18.0 ± 6.6% and 15.0 ± 1.3% of compound **7** in the medium after 10 days of biotransformation, respectively ((*p* value > 0.05), Supplementary Materials—Table S1).

The progress in the production of all metabolites over time by the tested filamentous fungi is shown in Figure S13 available in the Supplementary Materials.

## 3. Materials and Methods

### 3.1. General Experimental Methods

Reagents and solvents (analytical or HPLC grade) were purchased from Sigma-Aldrich (Merck Group, Darmstadt, Germany) or POCH (Avantor Performance Materials Poland, Gliwice, Poland). The components of the cultivation media were purchased from POCH (Avantor Performance Materials Poland, Gliwice, Poland) and BTL (Łódź, Poland). Xanthohumol (**1**) was isolated from spent hops—a side product obtained after the supercritical carbon dioxide extraction of hops (*Humulus lupulus* cv ‘Magnum’, crop 2015), obtained from the Production of Hops Extracts of the New Chemical Syntheses Institute (Puławy, Poland) and then purified according to methods described previously [42]. The evaluation of the progress of the biotransformation process was carried out by TLC and UHPLC analyses TLC analyses which were performed on silica gel 60, F254 (0.2 mm thick) plates from Merck (Darmstadt, Germany) with solvent mixtures CHCl_3_:MeOH (from 9:1 to 19:1 depending on experiment) and then after drying, spots were visualized under short- and long-wavelength UV light; then, plates were sprayed with a methanol–sulfuric acid (1:1, *v*/*v*) solution. The biotransformation products **2**–**4** and **6**–**7** were identified by comparison to standards that were obtained and published previously [19,20,23]. The biotransformation product **5** was purified by means of a PuriFlash PF430 flash chromatography system (Interchim, Montluçon, France), preparative TLC on silica gel GF, UV254 (20 × 20 cm, 2000 micron), and Uniplate^TM^ plates (Analtech, Miles Scientific, Newark, DE, USA). ^1^H NMR, ^13^C NMR, DEPT 135, ^1^H–^1^H NMR (COSY), and ^1^H–^13^C NMR (HSQC and HMBC) were recorded on a DRX Bruker Advance II 600 (600 MHz) instrument (Bruker, Billerica, MA, USA) in DMSO-d_6_. The NMR spectra were processed using MestReNova software (MestReNova v9.0, Mestrelab Research, Santiago de Compostela, Spain). Negative-ion HRESI-MS spectra were recorded on a Waters Xevo G2 QTof Quadrupole Time-of-Flight Mass spectrometer (Waters, Milford, MA, USA). The direct infusion of ESI-MS parameters was conducted. The mass spectrometer was operated in negative ion mode. The capillary voltage was set to 2.5 kV, with a desolvation gas flow rate of 150 L/h at 250 °C. The Lock Spray infusion flow rate was 10 µL/min, and the injection volume of samples was 5 µL. Ionization mass spectra were collected at the ranges *m*/*z* 70–600. The instrument was calibrated using a sodium formate QToF calibration solution prepared according to Waters Knowledge Base WKB2458 procedure (*m*/*z* range 113.1890–1131.8860). An MS-MS experiment was performed Using Waters Auto MS/MS mode (collision energy 6 eV). ATR-IR analysis was carried out using a Thermo Scientific Nicolet iS10 spectrometer (Thermo Fisher Scientific, Waltham, MA, USA). Spectra were taken from 4000 to 520 cm^−1^ over 32 scans, with a spectral resolution of 4 cm^−1^ and a blank window for the background. IR spectrum was processed using OMNIC Software (OMNIC v8.3, Thermo Fisher Scientific, Waltham, MA, USA). UV spectra were recorded on a Cintra 303 spectrophotometer (GBC Scientific Equipment, Braeside, Australia) in methanol.

### 3.2. Microorganisms

The microorganisms *B. bassiana* KCh BBT, *B. bassiana* KChJ 1.5, *B. caledonica* KCh J3.3, *B. feline* ENC3, *I. farinosa* KCh KW1.1, *I. fumosorosea* KCh J2, *I. tenuipes* MU35, and *M. robertsii* MU4 were obtained from the collection of the Department of Food Chemistry and Biocatalysis, Wrocław University of Environmental and Life Sciences (Wrocław, Poland).

### 3.3. Conditions for Biotransformation

#### 3.3.1. Study of the Biotransformation Process over Time

Erlenmeyer flasks of a 300 mL volume containing 100 mL of the sterile cultivation medium (3% glucose and 1% Aminobak) were inoculated with a suspension of each entomopathogenic strain and then incubated for 4 days at 24 °C on a rotary shaker. After this time, 10 mg of a xanthohumol (**1**) dissolved in 250 µL of dimethyl sulfoxide (DMSO) was added to each culture. Samples (10 mL of the cultures) were collected on the 1st, 3rd, 7th, and 10th day of the process, and extracted using 10 mL of ethyl acetate. The obtained extracts were concentrated in vacuo, suspended in 1 mL of methanol (HPLC grade), centrifuged for 10 min (21,000× *g*), and analyzed using TLC and UHPLC methods. Each experiment was performed in triplicate.

#### 3.3.2. Scale-Up Biotransformation

To obtain the unknown metabolite **5**, a 2000 mL flask containing 500 mL of the sterile cultivation medium (3% glucose, 1% Aminobak) was inoculated with a culture of *B. bassiana* KCh BBT in the same way as described above. Four days after inoculation, 120 mg of substrate **1** dissolved in 3 mL of DMSO was added to each culture. The progress of the biotransformation process was evaluated in 2-day intervals. Collected samples as described above were analyzed using TLC and UHPLC methods. The biotransformation process was carried out until the substrate had almost completely reacted and no further transformation was observed (after 6 days, biotransformation was stopped). The products were extracted three times using ethyl acetate. Organic fractions were combined, dried using anhydrous MgSO_4_, concentrated in vacuo, and analyzed using TLC and UHPLC methods.

### 3.4. Purification of (2″E)-4″-Hydroxyxanthohumol 4′-O-β-D-(4‴-O-Methyl)-Glucopyranoside (***5***)

The crude biotransformation products were purified by means of a PuriFlash PF430 flash chromatography system (Interchim, Montluçon, France) using a PF-30SIHP-F0025-30 µm flash column (Interchim, Montluçon, France) with isocratic elution of a chloroform:methanol:formic acid (900:100:2, *v*/*v*) solvent mixture at a flow rate of 20 mL/min and detection at λ = 280 nm and λ = 370 nm wavelengths. The collected fractions (15 mL) were analyzed by TLC and UHPLC. The fraction containing metabolite **5** was combined, evaporated, and re-purified by the preparative TLC method on silica gel GF using Uniplate^TM^ plates (Analtech, Miles Scientific, Newark, DE, USA) and the mixture of acetone: dichloromethane:hexane:formic acid (70:50:30:1, *v*/*v*). The fraction containing metabolite **5** was scraped from the dried developed chromatogram, extracted 4 times with methanol, and then evaporated to yellow amorphous powder and analyzed by UHPLC (~98% purity) and NMR.

### 3.5. (2″E)-4″-Hydroxyxanthohumol 4′-O-β-D-(4‴-O-Methyl)-Glucopiranoside (***5***)

Compound **5** was isolated after a 6-day transformation of xanthohumol (**1**) (13.6 mg, yield 8.84%). ^1^H NMR 600 MHz, DMSO-d_6_, δ [ppm]: 14,21 (1H, s, C2′-OH), 7.74 (1H, d, *J* = 15.5 Hz, H-α), 7.70 (1H, d, *J* = 15.5 Hz, H-β), 7.59 (2H, d, *J* = 8.2 Hz, H-2, H-6), 6.85 (2H, d, *J* = 8.2 Hz, H-3, H-5), 6.37 (1H, s, H-5′), 5.44 (1H, bs, -OH), 5.37 (1H, t, *J* = 7.3 Hz, H-2″), 5.33 (1H, bs, -OH), 5.04 (1H, d, *J* = 7.7 Hz, H-1‴), 4.85 (1H, bm, -OH), 4.62 (1H, bm, -OH), 3.92 (3H, s, C6′-OCH_3_), 3.73 (2H, s, H-4″), 3.67 (1H, m, H-6″b), 3.50 (1H, m, H-6″a, overlap with H-5‴), 3.50 (1H, m, H-5‴, overlap with H-6″a), 3.46 (3H, s, C4″-OCH_3_, overlap with H-3‴), 3.45 (1H, m, H-3‴, overlap with C4″-OCH_3_), 3.40 (1H, m, H-1″a), 3.33 (1H, m, H-2‴), 3.16 (1H, m, H-1″b), 2.99 (1H, m, H-4‴), 1.70 (3H, s, H-5″). ^13^C NMR 151 MHz, DMSO-d_6_, δ [ppm]: 192.6 (C=O), 163.2 (C-2′), 161.1 (C-4′), 160.6 (C-6′), 160.3 (C-4), 143.4 (C-β), 134.8 (C-3″), 130.8 (C-2, C-6), 125.9 (C-1), 123.6 (C-α), 121.9 (C-2″), 116.1 (C-3, C-5), 109.3 (C-3′), 106.2 (C-1′), 99.8 (C-1‴), 90.6 (C-5′), 79.5 (C-4‴), 76.6 (C-3‴), 76.1 (C-5‴), 73.6 (C-2‴), 66.5 (C-4″), 60.5 (C-6‴), 59.8 (C4‴-OCH_3_), 56.1 (C6′-OCH_3_), 20.8 (C-1″), 13.7 (C-5″). HR ESI-MS: *m*/*z* calculated for C_28_H_34_O_11_-H ([M-H]^−^): 354.2028. Found 545.2027 [M-H]^−^. UV (MeOH) λ_max_: 369.39 nm.

### 3.6. Ultra High-Performance Liquid Chromatography

The UHPLC analyses were performed on an Ultimate 3000 UHPLC+ focused instrument (Thermo Scientific, Waltham, MA, USA) equipped with a DGP-3600A dual pump liquid control compartment, a TCC-3200 thermostated column module, a WPS-3000 autosampler, and a photodiode array detector (detection from 210 to 450 nm wavelength) using an analytical C-18 UHPLC column Thermo Scientific Acclaim RSLC Polar Advantage II analytical (2.1 mm × 100 mm, 2.2 µm, Thermo Scientific, Waltham, MA, USA) thermostated at 28 °C at a flow rate of 0.7 mL/min and the following elution program: gradient elution: from 0 to 3 min. (85% A → 2% A), isocratic elution: from 3 to 4.2 min. (2% A), gradient elution: from 4.2 to 4.4 min (2% A → 85% A), isocratic elution: from 4.4 to 6.0 min (85% A). Solvent A consisted of 0.1% HCOOH in water and solvent B consisted of 0.1% HCOOH in acetonitrile. The amounts of xanthohumol (**1**), isoxanthohumol (**2**), and their glycosides in the extracts after completing the 1, 3, 7, and 10 days of biotransformation process were determined using standard calibration curves and using the peak area relationship between xanthohumol (**1**) and its glucosides (detection at wavelength λ = 370 nm), isoxanthohumol (**2**) and its glucosides (detection at wavelength λ = 280 nm). The individual standard stock solutions of aglycones and glucosides (purity (UHPLC) ≥ 98%) were diluted to a series of different concentration solutions for constructing the calibration curves in the following concentration ranges: 55–440 µg/mL for xanthohumol (**1**), 48–384 µg/mL for soxanthohumol (**2**), 51–408 µg/mL for xanthohumol 4′-*O*-glucoside (**3**), 69–552 µg/mL for xanthohumol 4′-*O*-(4‴-*O*-methyl)-glucoside (**4**), 52.5–420 µg/mL for isoxanthohumol 7-*O*-glucoside (**6**), and 51.5–412 µg/mL for isoxanthohumol 7-*O*-(4‴-*O*-methyl)-glucoside (**7**). The mixtures of the standard solutions were injected in triplicate (5 µL), and the calibration curves were constructed by plotting the peak area (*Y*-axis) versus the concentration (*X*-axis) of each analyte. The results of all tested compounds showed good linearity: R^2^ = 0.9976 for xanthohumol (**1**), R^2^ = 0.9949 for isoxanthohumol (**2**), R^2^ = 0.9998 for xanthohumol 4′-*O*-glucoside (**3**), R^2^ = 0.9995 for xanthohumol 4′-*O*-(4‴-*O*-methyl)-glucoside (**4**), R^2^ = 0.9995 for isoxanthohumol 7-*O*-glucoside (**6**), and R^2^ = 0.9970 for isoxanthohumol 7-*O*-(4‴-*O*-methyl)-glucoside (**7**). Data acquisition and analysis were performed using the Chromeleon software workstation (v7.2.10) (Thermo Scientific, Waltham, MA, USA).

### 3.7. Statistical Analysis

The UHPLC conversion data are presented as the mean ± standard deviation of the mean (SD). Statistical analysis was performed with a one-way ANOVA with Tukey’s post hoc test (Excel Office ver. 2019 with Real Statistics Resource Pack, Microsoft, Redmond, WA, USA). The significance was accepted at a *p* value of <0.05. All statistical data are available in the Supplementary Materials (Pages S16–S58).

## 4. Conclusions

The study revealed that seven strains of entomopathogenic filamentous fungi demonstrated significant xanthohumol (**1**) biotransformation activity. Six biotransformation products were identified, including three xanthohumol glycosides (**3**–**5**), isoxanthohumol (**2**), and two glycosides of isoxanthohumol (**6** and **7**). Notably, a novel derivative, (2″*E*)-4″-hydroxyxanthohumol 4′-*O*-β-D-(4‴-*O*-methyl)-glucopyranoside (**5**), was characterized, marking the first report of this compound. Interestingly, the biotransformations of xanthohumol (**1**) in which this compound was produced at an early stage, were significantly slowed down, which may suggest that it has antifungal activity. However, these properties need to be confirmed in further studies. The findings indicate that entomopathogenic fungi possess significant potential as biocatalysts for the regioselective modification of flavonoid structures, thereby enhancing their chemical diversity and biological properties. The ability of these fungi to isomerize xanthohumol (**1**) into isoxanthohumol (**2**) and glucosides of xanthohumol (**4** and **5**) into the glucosides of isoxanthohumol (**6** and **7**) proves the presence of chalcone isomerase-like activity, an enzyme typically found in plant species. The successful glycosylation of xanthohumol (**1**) improves its solubility, suggesting potential applications in enhancing its bioavailability and therapeutic efficacy. The results provide a promising foundation for future research focused on the optimization of these biotransformation processes and the exploration of the therapeutic potential of the resulting glycosylated flavonoids.

## Figures and Tables

**Table 1:** Capability of transforming xanthohumol (**1**) by tested entomopathogenic filamentous fungi.

Strain Tested	Capability of Transforming Xanthohumol (1) *
*Beauveria bassiana* KCh J1.5	++
*Beauveria bassiana* KCh BBT	+
*Beauveria caledonica* KCh J3.3	++
*Beauveria feline* ENC3	−
*Isaria fumosorosea* KCh J2	++
*Isaria farinosa* KCh KW1.1	++
*Isaria tenuipes* MU35	+
*Metarhizium robertsii* MU4	++

* (++) less than 5% of substrate remaining after 10 days of biotransformation, (+) from 10% to 15% of substrate remaining after 10 days of biotransformation, and (−) no product(s) observed (by UHPLC).

## Data Availability

Data are contained within the article and the Supplementary Materials.

## Supplementary Materials

The following supporting information can be downloaded at: https://www.mdpi.com/article/10.3390/ijms251910433/s1.
