# Supplementary material for: Biotransformation of Xanthohumol by Entomopathogenic Filamentous Fungi
Source: Int J Mol Sci. 2024 Sep 27;25(19):10433. doi: 10.3390/ijms251910433 (PMC11477118; doi:10.3390/ijms251910433)
Supplement: Supplementary file 1 [file ijms-25-10433-s001.zip › ijms-3220023-supplementary.pdf]

Supplementary data

Biotransformation of Xanthohumol by Entomopathogenic Filamentous Fungi

Daniel Łój[1], Tomasz Janeczko[1,*], Agnieszka Bartmańska[1], Ewa Huszcza[1], and Tomasz Tronina[1,*]

[1] Department of Food Chemistry and Biocatalysis, Wrocław University of Environmental and Life Sciences, Norwida 25, 50-375 Wrocław, Poland

<u>**Table of Contents:**</u> **Page**

**UV spectra of xanthohumol (1), isoxanthohumol (2) and its glycosides (3-7)**	**S2**
**UHPLC chromatograms of xanthohumol (1), isoxanthohumol (2) and its glycosides (3-7)**	**S3-S5**
**NMR Spectra**	**S6-S10**
*^{1}H NMR spectra of: **A** xanthohumol (**1**), **B** (2″E)-4”-hydroxyxanthohumol 4’-O-β-D-(4’’’-O-methyl)-glucopiranoside (**5**)*	S6
*^{13}C NMR spectra of: **A** xanthohumol (**1**), **B** (2″E)-4”-hydroxyxanthohumol 4’-O-β-D-(4’’’-O-methyl)-glucopiranoside (**5**)*	S7
*Overlaid fragments of ^{13}C NMR and DEPT 135 spectra of (2″E)-4”-hydroxyxanthohumol 4’-O-β-D-(4’’’-O-methyl)- glucopiranoside (**5**)*	S8
*^{1}H-^{1}H NMR (COSY) spectrum of 4”-hydroxyxanthohumol (2″E)-4’-O-β-D-(4’’’-O-methyl)-glucopiranoside (**5**)*	S9
*^{1}H– ^{13}C NMR (HSQC) spectrum of 4”-hydroxyxanthohumol (2″E)-4’-O-β-D-(4’’’-O-methyl)-glucopiranoside (**5**)*	S9
*Fragment of ^{1}H– ^{13}C NMR (HSQC) spectrum of (2″E)-4”-hydroxyxanthohumol 4’-O-β-D-(4’’’-O-methyl)-glucopiranoside (**5**)*	S10
*^{1}H– ^{13}C NMR (HMBC) spectrum of (2″E)-4”-hydroxyxanthohumol 4’-O-β-D-(4’’’-O-methyl)-glucopiranoside (**5**)*	S10
**HR-ESI MS Spectrum of (2″*E*)-4”-hydroxyxanthohumol 4’-*O*-β-D-(4’’’-*O*-methyl)- glucopiranoside (5)**	**S11**
**HR MS-MS Spectrum of (2″*E*)-4”-hydroxyxanthohumol 4’-*O*-β-D-(4’’’-*O*-methyl)- glucopiranoside (5)**	**S12**
**ATR IR Spectrum of (2″*E*)-4”-hydroxyxanthohumol 4’-*O*-β-D-(4’’’-*O*-methyl)- glucopiranoside (5)**	**S13**
**Microbial transformation of xanthohumol (1), UHPLC conversion**	**S14**
**Progress in the production of metabolites 2-7**	**S15**
**Statistical Analysis**	**S16-S58**

Figure S1*:* **UV spectra of xanthohumol (1), isoxanthohumol (2) and its glycosides (3-7)**

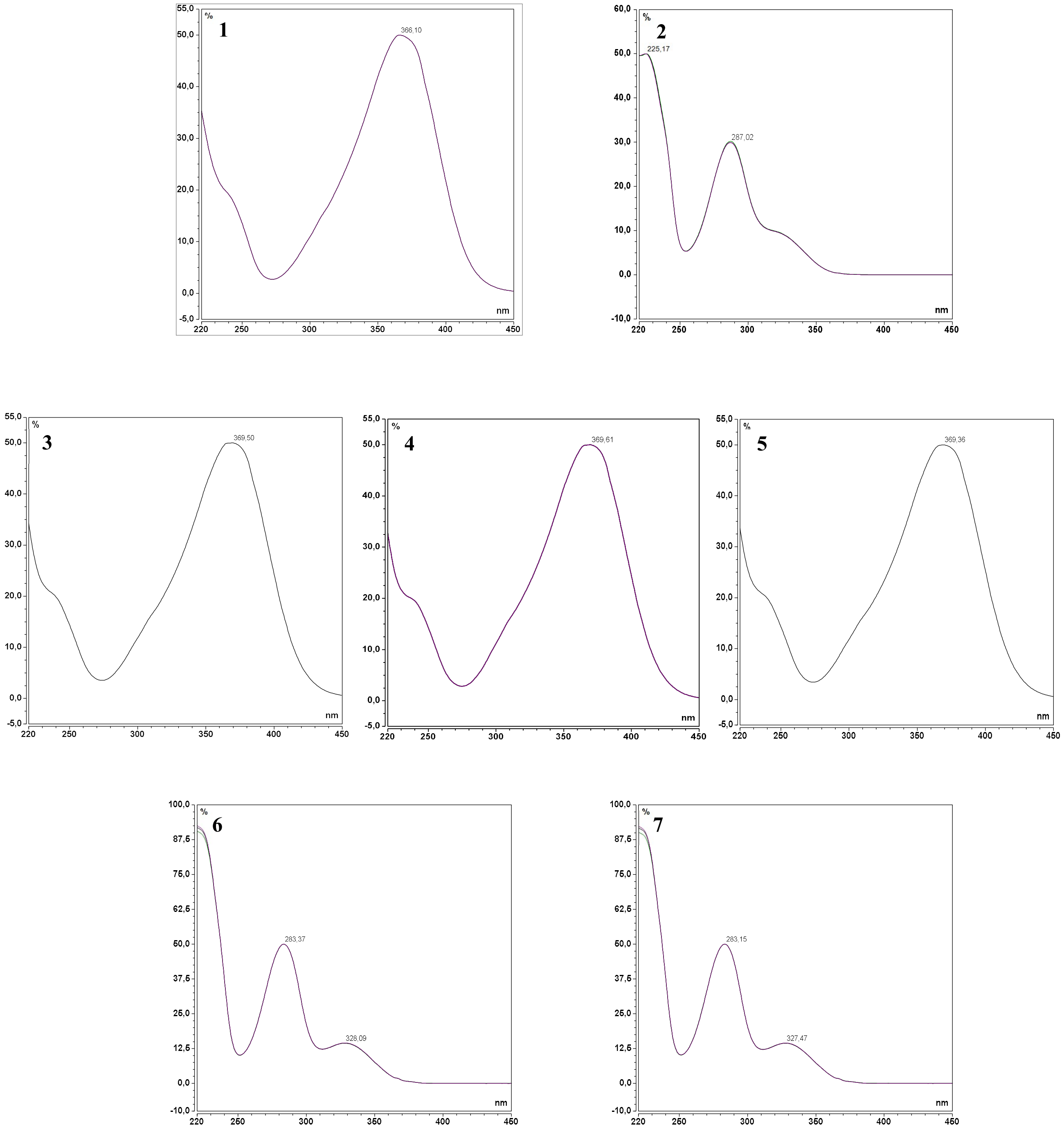

Chalcones**:** xanthohumol (**1**), xanthohumol 4’-O-*β*-D-glucopyranoside (**3**), xanthohumol 4’-O-*β*-D-(4’’’-O-methyl)-glucopyranoside (**4**), (2”E)-4”-hydroxyxanthohumol 4’-O-*β*-D-(4’’’-O-methyl)-glucopyranoside (**5**)

Flavanones**:** isoxanthohumol (**2**), isoxanthohumol 7-O-*β*-D-glucopyranoside (**6**), isoxanthohumol 7-O-*β*-D-(4’’’-O-methyl)-glucopyranoside (**7**),

Figure S2: UHPLC chromatograms of xanthohumol (1), isoxanthohumol (2) and its glycosides (3-7)

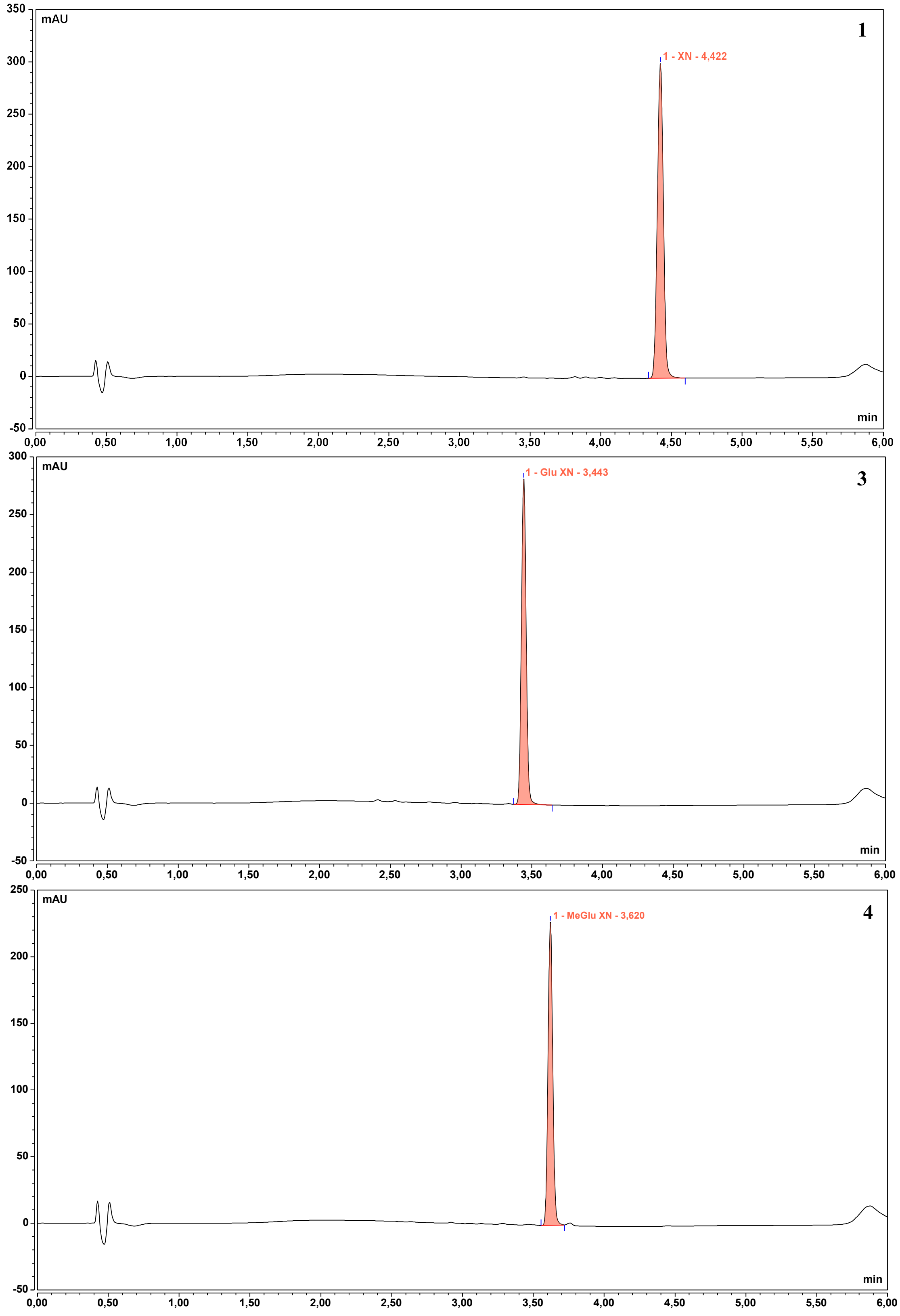

Figure S2*:* **UHPLC chromatograms of xanthohumol (1), isoxanthohumol (2) and its glycosides (3-7)** ***cont.***

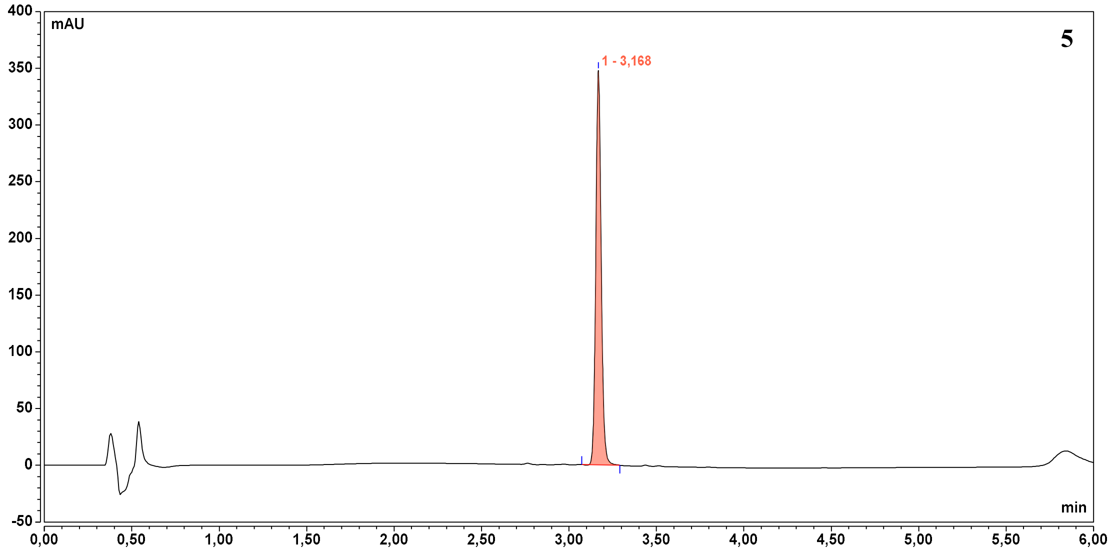

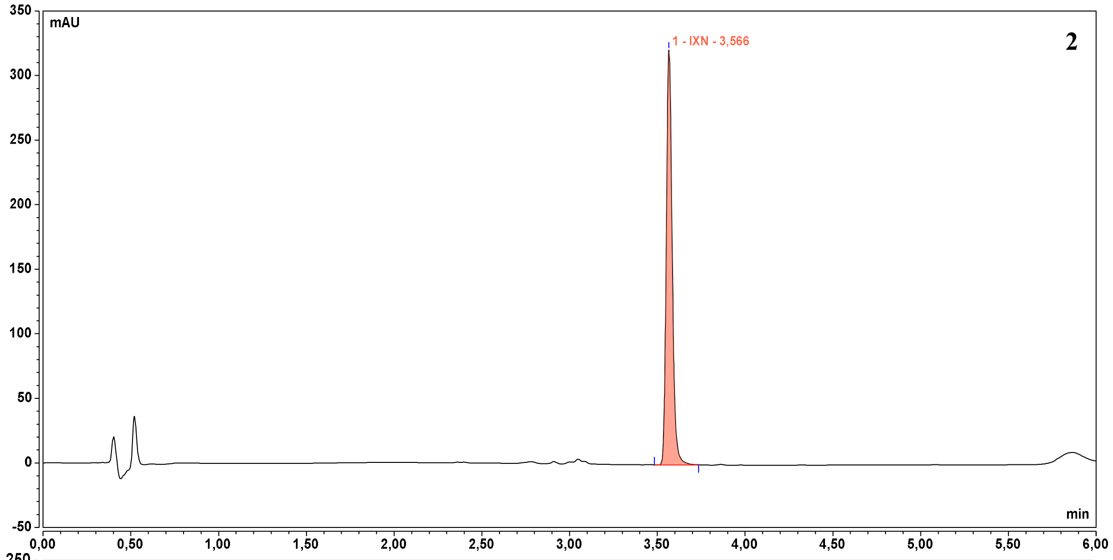

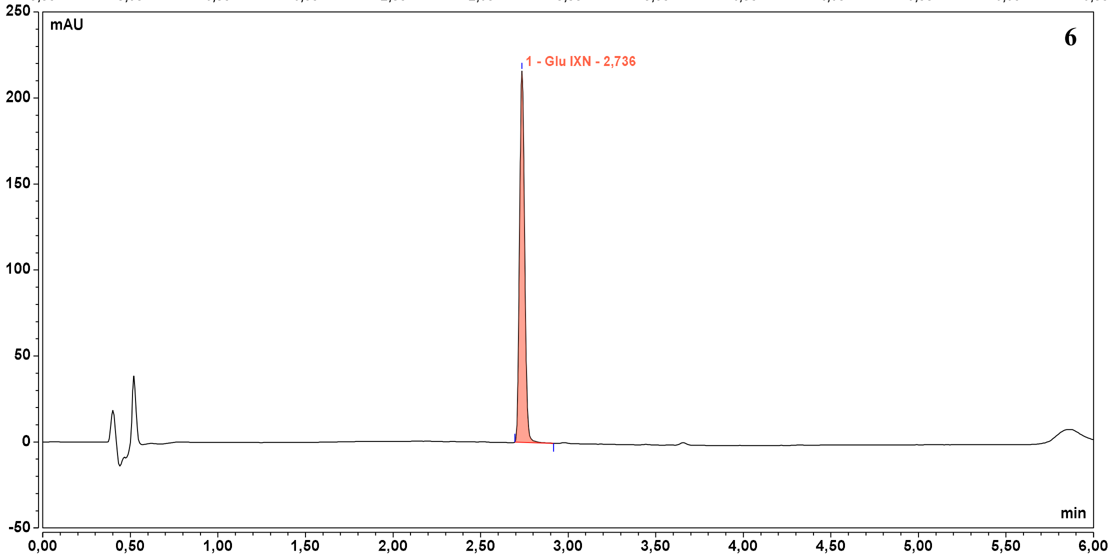

Figure S2: **UHPLC chromatograms of xanthohumol (1), isoxanthohumol (2) and its glycosides (3-7)** ***cont.***

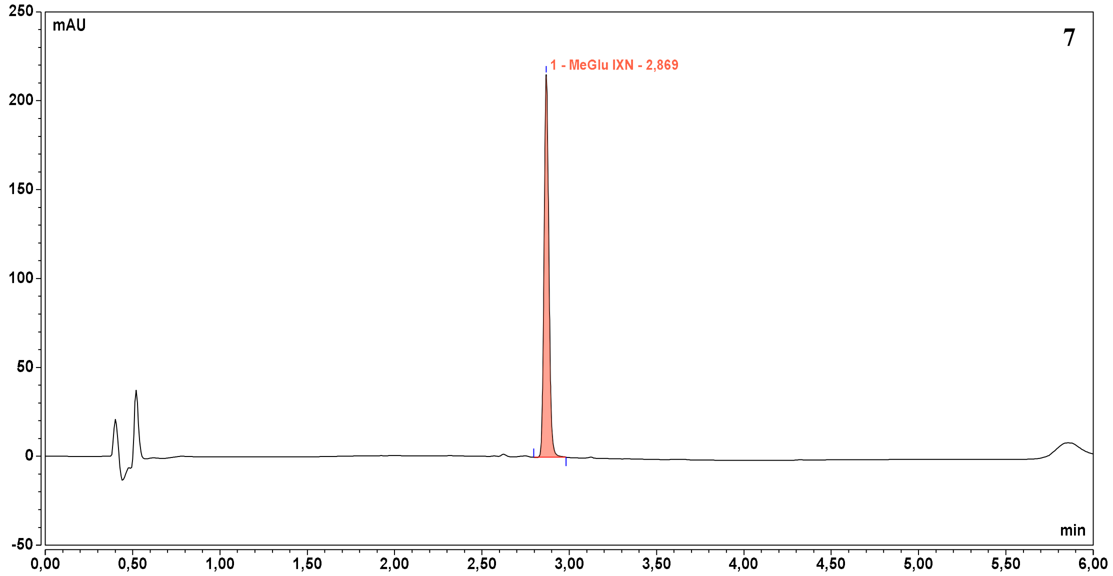

***Chalcones*:**
xanthohumol (**1**), xanthohumol 4'-O-β-D-glucopyranoside (**3**), xanthohumol 4'-O-β-D-(4'''-O-methyl)-glucopyranoside (**4**), (2''E)-4''-hydroxyxanthohumol 4'-O-β-D-(4'''-O-methyl)-glucopyranoside (**5**)
(Detection at λ_{max} = 370 nm.)

***Flavanones*:**
isoxanthohumol (**2**), isoxanthohumol 7-O-β-D-glucopyranoside (**6**), isoxanthohumol 7-O-β-D-(4'''-O-methyl)-glucopyranoside (**7**),
(Detection at λ_{max} = 280 nm.)

Figure S3. *^{1}H NMR spectra of:* ***A*** *xanthohumol (**1**),* ***B*** *(2''E)-4''-hydroxyxanthohumol 4'-O-β-D-(4'''-O-methyl)- glucopiranoside (**5**)*

A

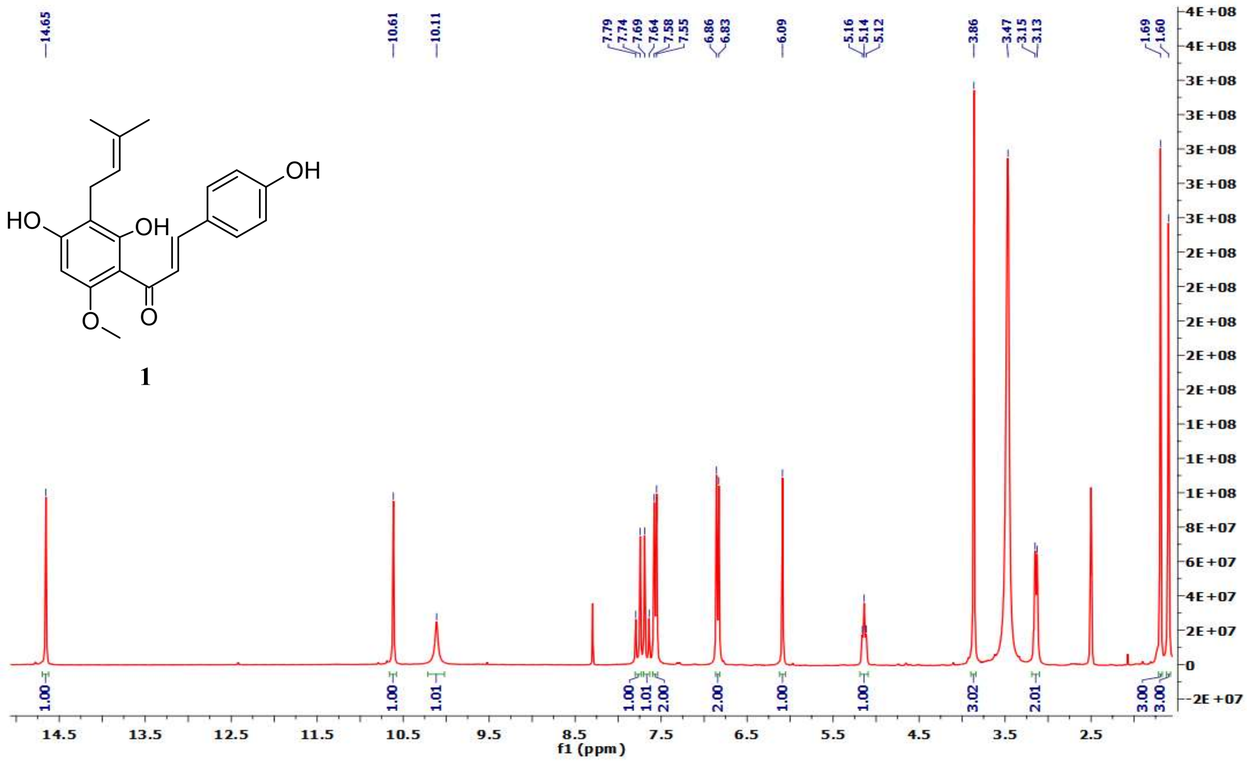

1

B

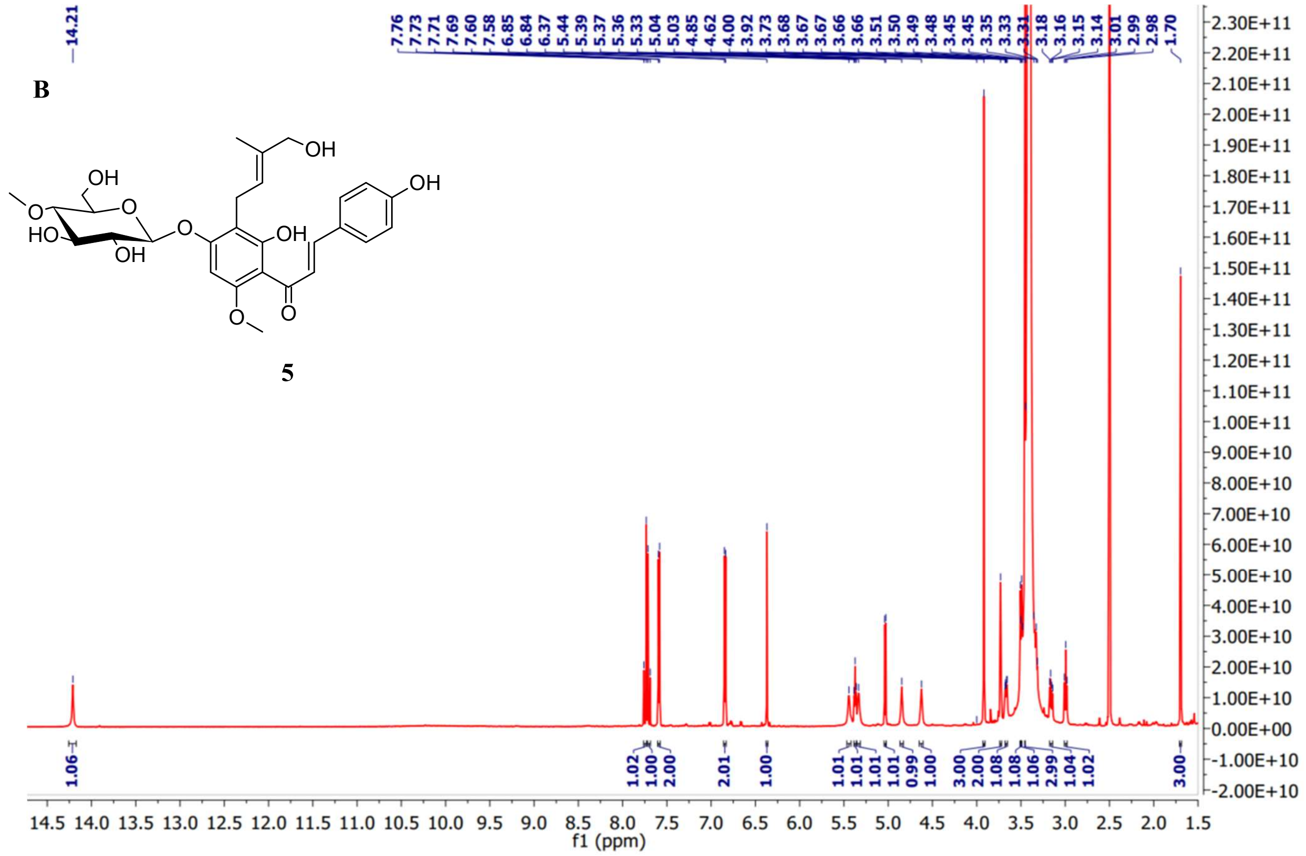

5

Figure S4. ^{13}*C NMR spectra of:* ***A*** *xanthohumol* ***(1)***, ***B*** *(2"E)-4"-hydroxyxanthohumol 4'-O-β-D-(4'''-O-methyl)- glucopiranoside* ***(5)***

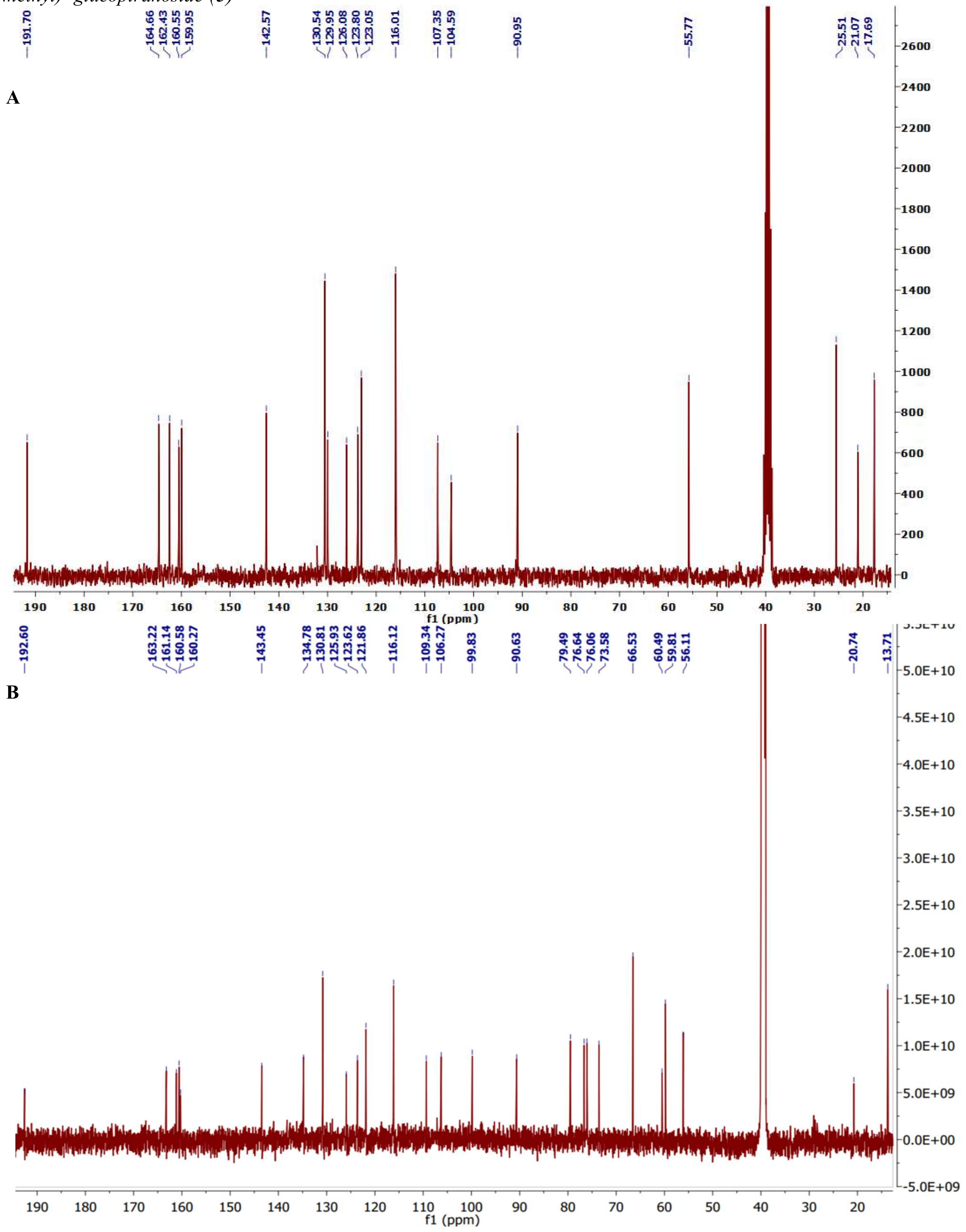

Figure S5. *Overlaid fragments of ^{13}C NMR and DEPT 135 spectra of (2"E)-4"-hydroxyxanthohumol 4'-O-β-D-(4'''-O-methyl)- glucopiranoside* ***(5)***

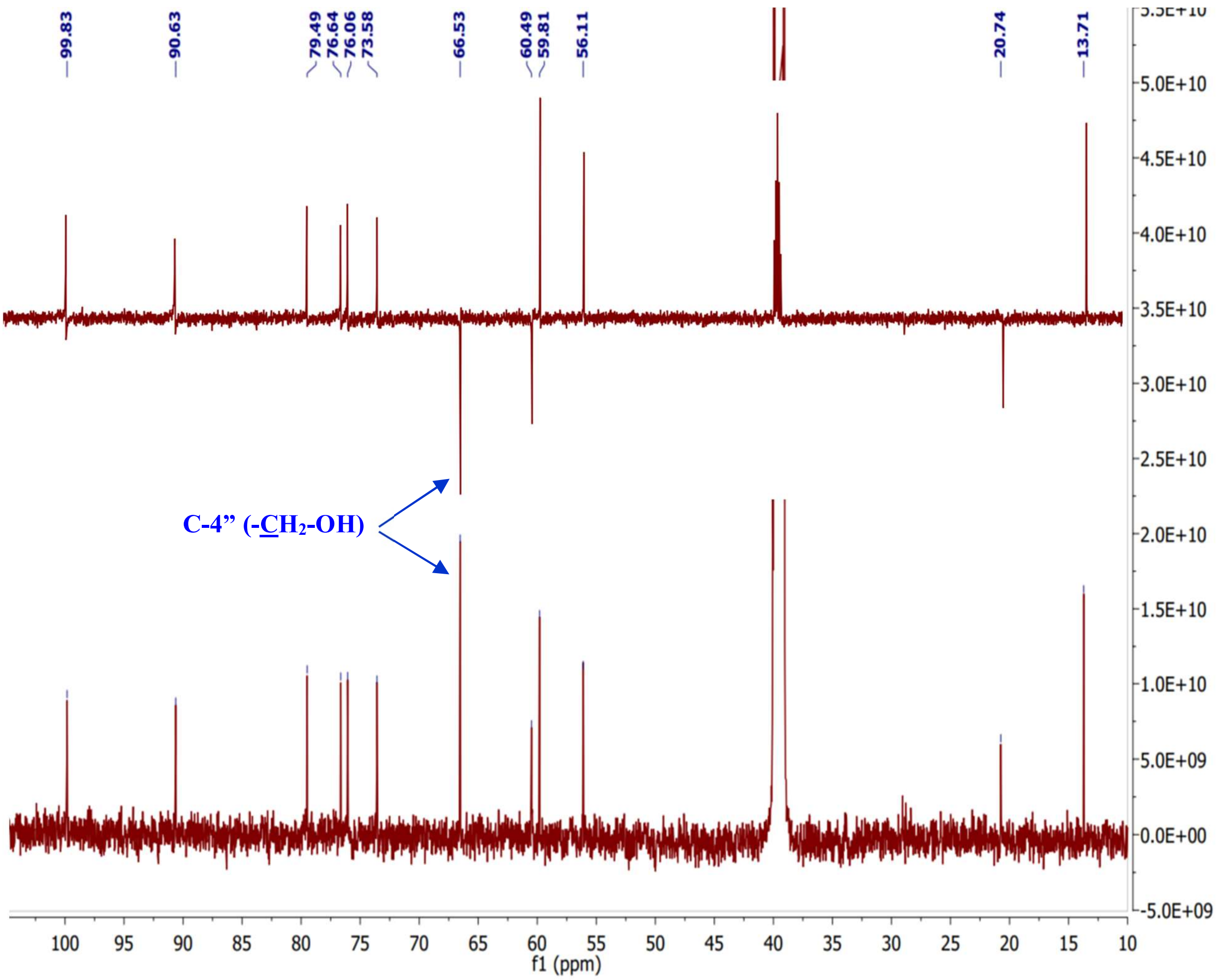

Figure S6. *^{1}H-^{1}H NMR (COSY) spectrum of (2''E)-4''-hydroxyxanthohumol 4'-O-β-D-(4'''-O-methyl)-glucopiranoside* (**5**)

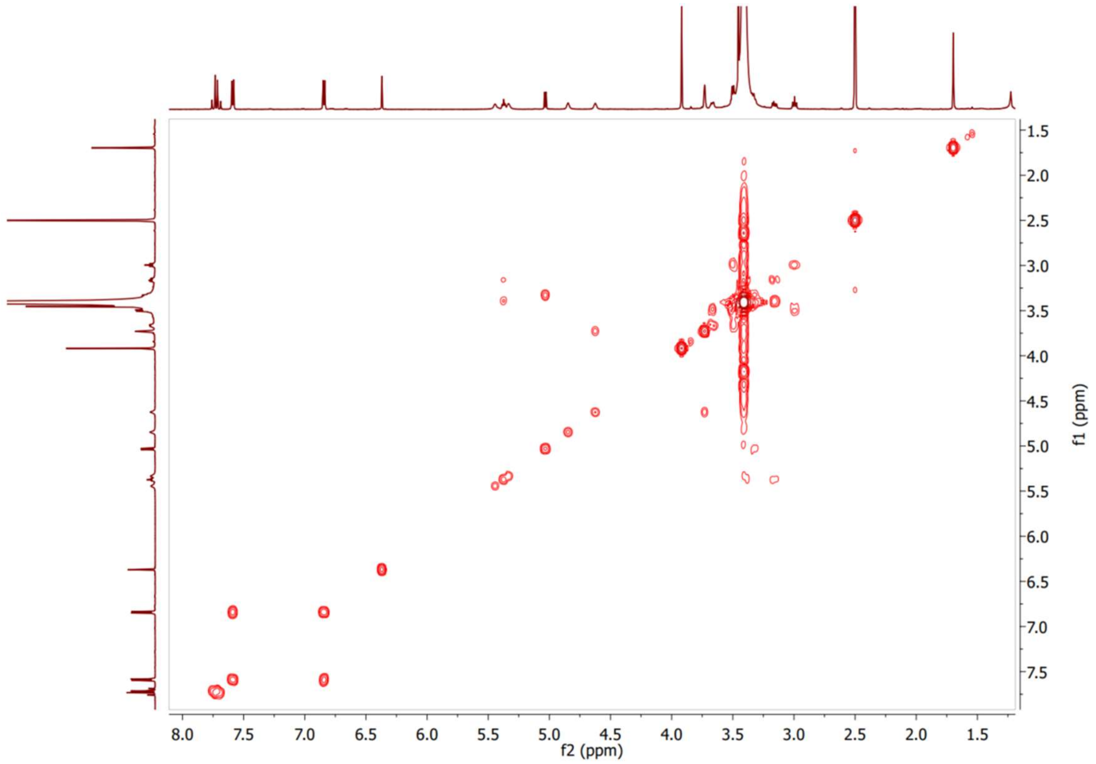

Figure S7. *^{1}H– ^{13}C NMR (HSQC) spectrum of (2''E)-4''-hydroxyxanthohumol 4'-O-β-D-(4'''-O-methyl)-glucopiranoside* (**5**)

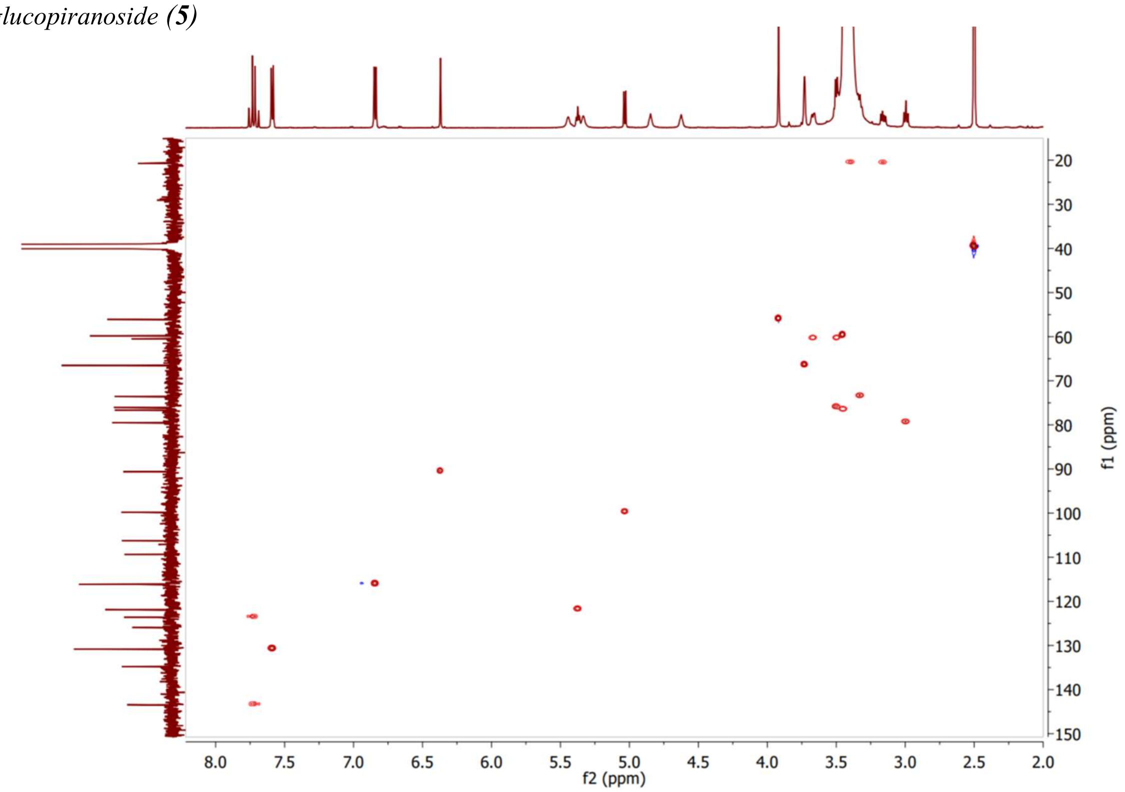

Figure S8. *Fragment of* ^{1}H*–* ^{13}C *NMR (HSQC) spectrum of (2''E)-4''-hydroxyxanthohumol 4'-O-β-D-(4'''-O-methyl)- glucopiranoside* ***(5)***

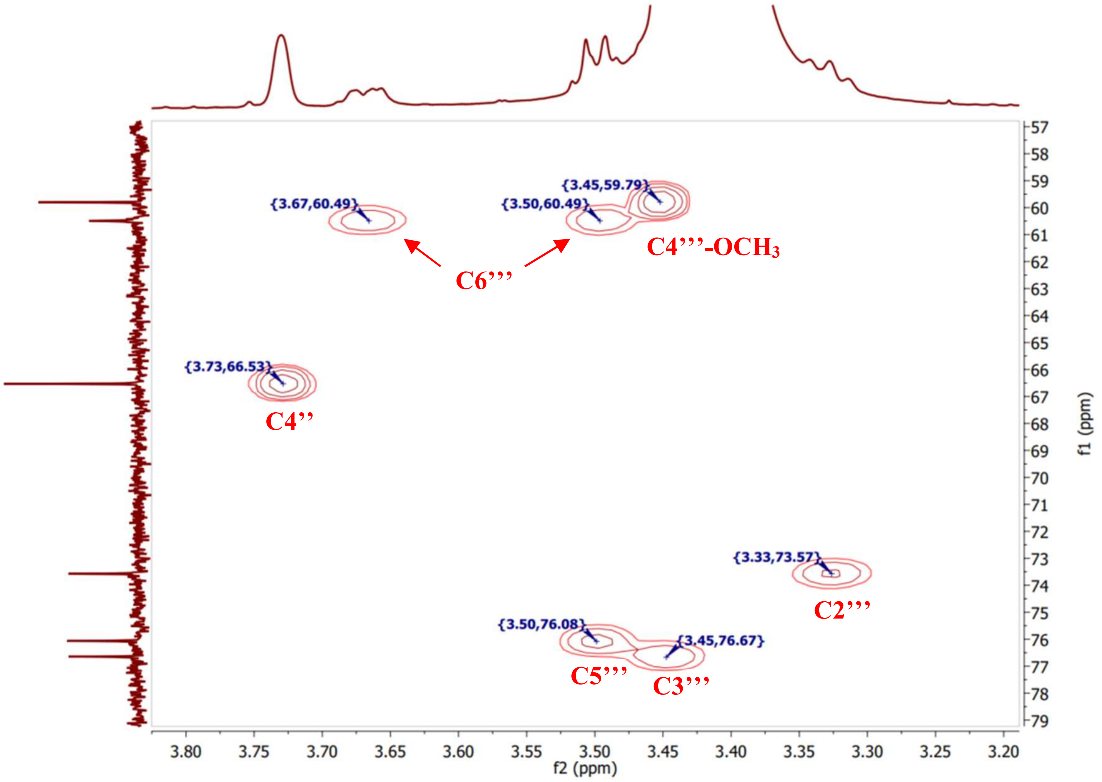

Figure S9. ^{1}H*–* ^{13}C *NMR (HMBC) spectrum of (2''E)-4''-hydroxyxanthohumol 4'-O-β-D-(4'''-O-methyl)- glucopiranoside* ***(5)***

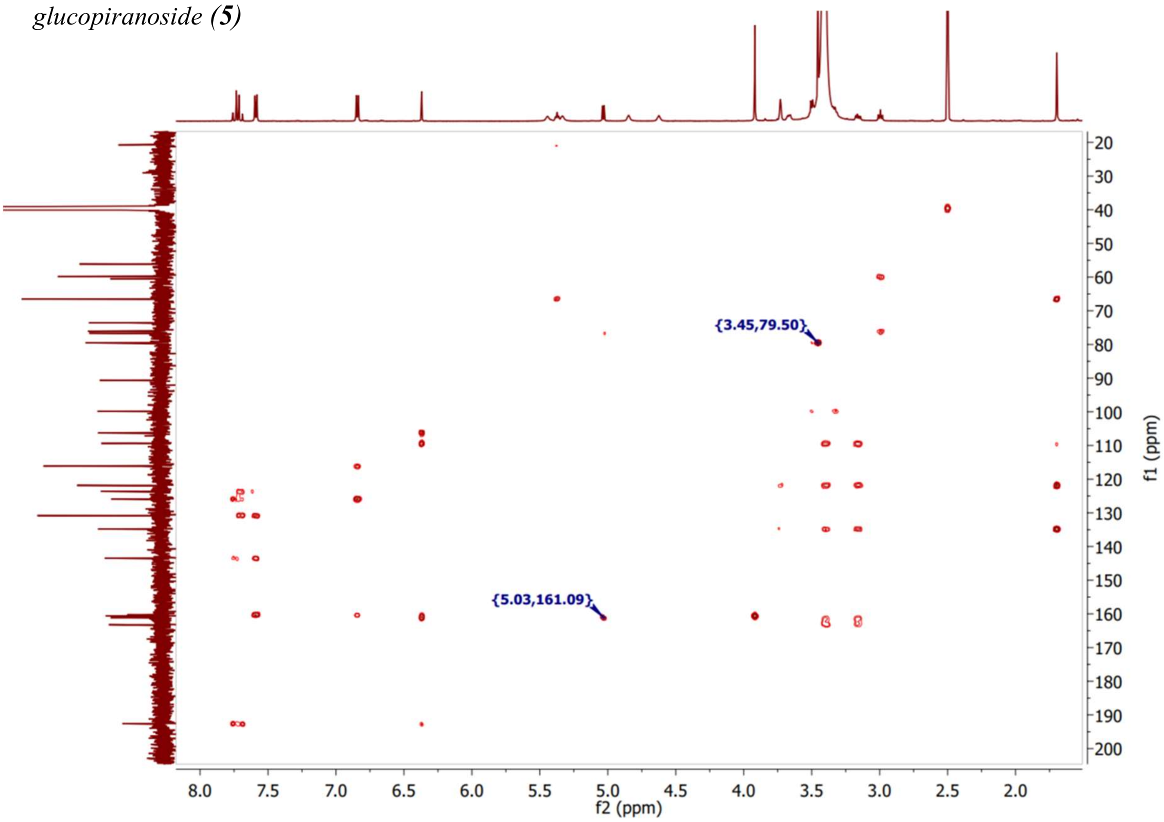

Figure S10. HR-ESI MS Spectrum of (2”*E*)-4”-hydroxyxanthohumol 4’-*O*-β-D-(4’’’-*O*-methyl)-glucopiranoside (5)

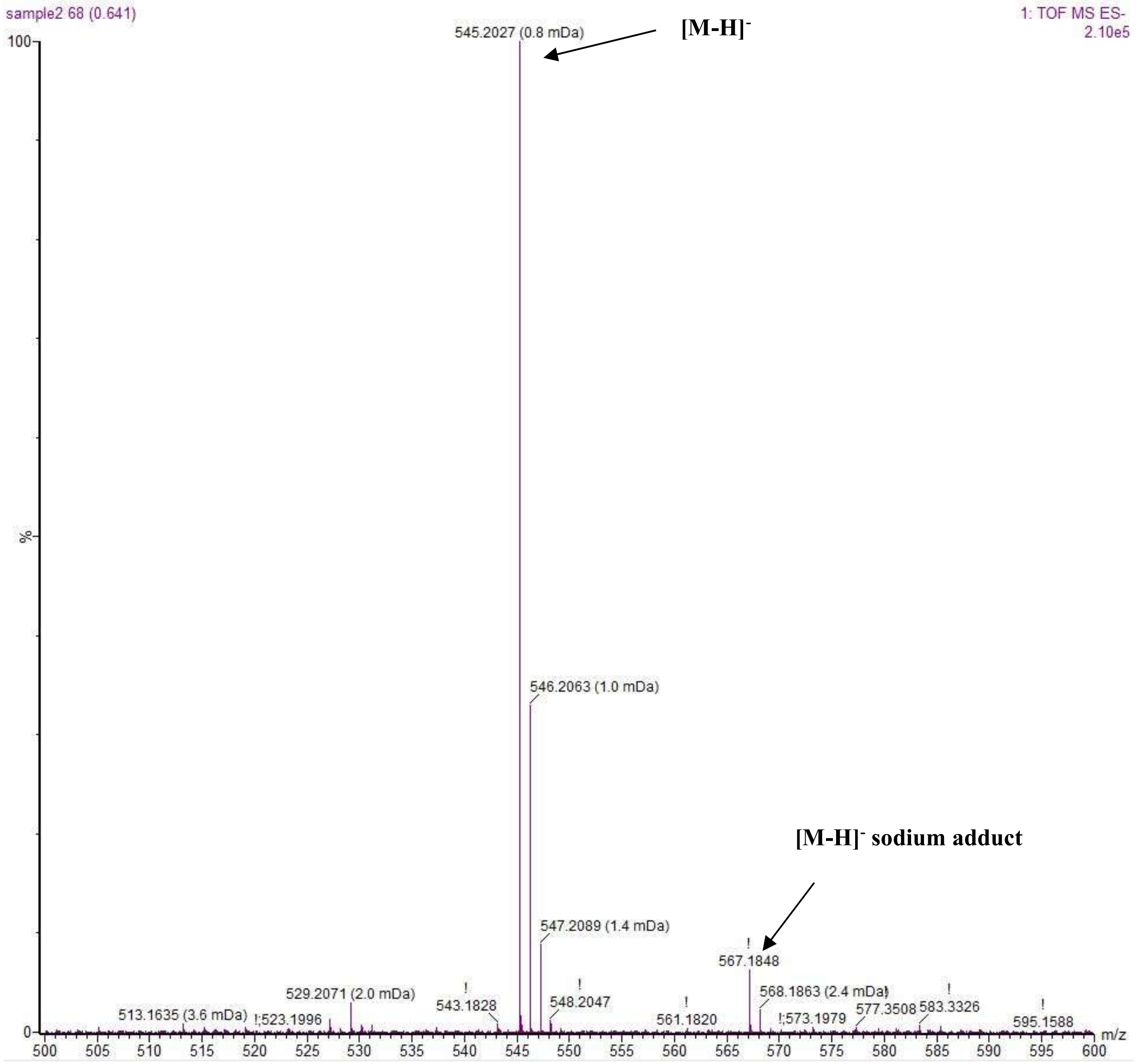

Figure S11. HR-ESI MS-MS Spectrum of (2”*E*)-4”-hydroxyxanthohumol 4’-*O*-β-D-(4’’’-*O*-methyl)-glucopiranoside (5)

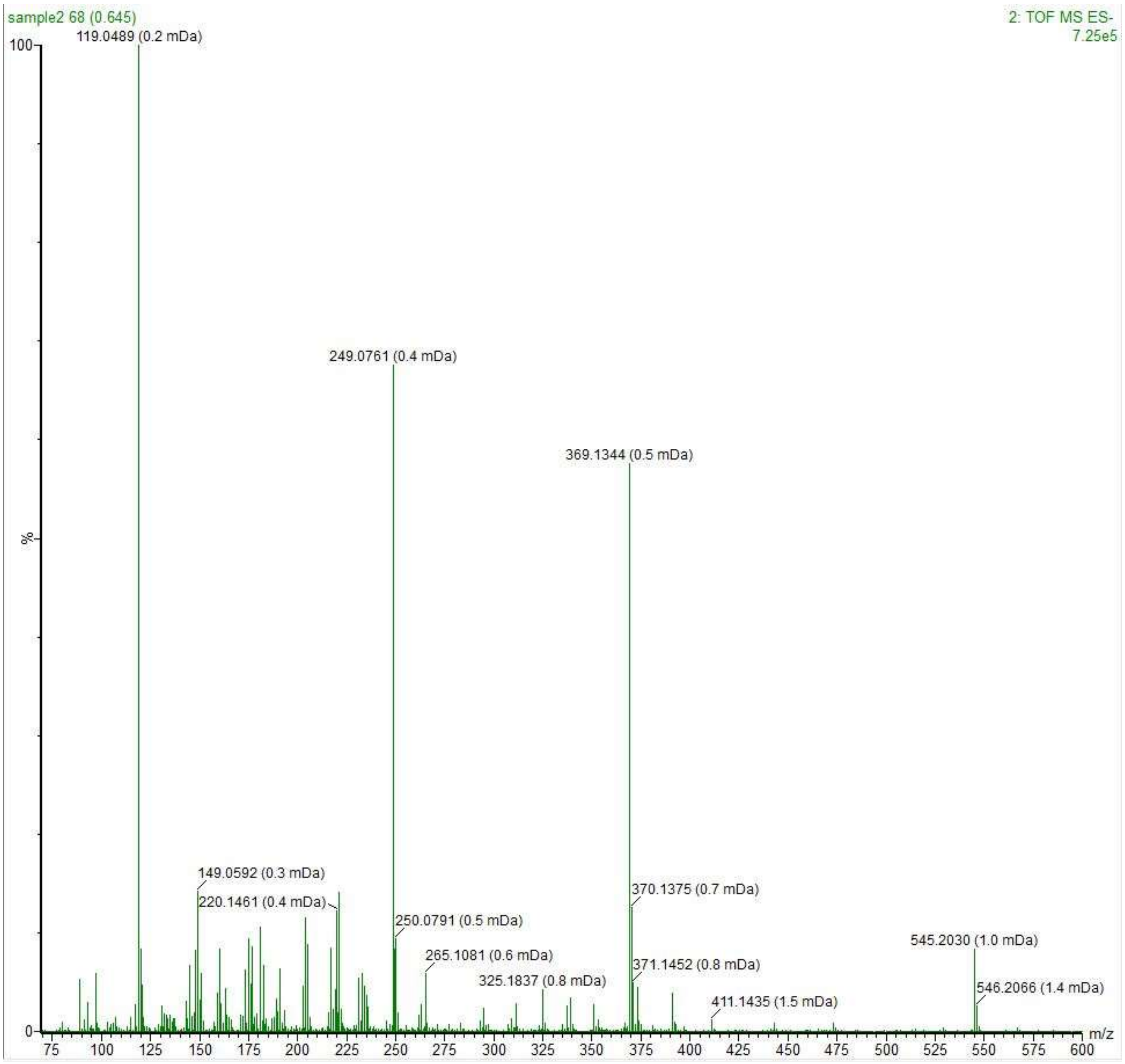

Figure S12. ATR IR Spectrum of (2”*E*)-4”-hydroxyxanthohumol 4’-*O*-β-D-(4’’’-*O*-methyl)- glucopiranoside (5)

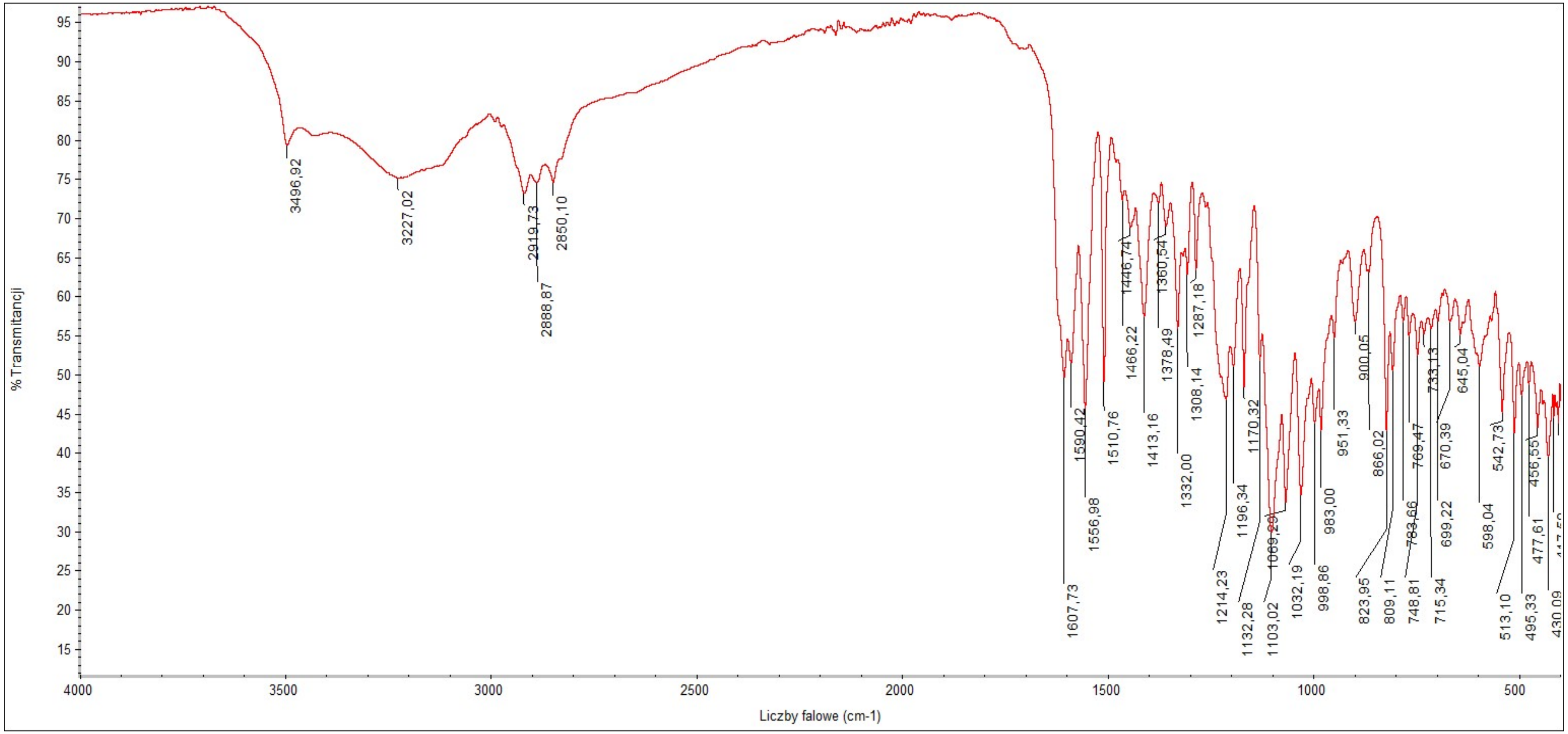

X-asix: wavenumber (cm^{-1})
Y-asix: % of transmitation

Table S1. *Microbial transformation of xanthohumol (**1**), UHPLC conversion*

Strain	Time of biotransformation [days]	Substrate [%]	Conversion [%] after 1,3,7 and 10 days of Biotransformation						
		**1**	**2**	**3**	**4**	**5**	**6**	**7**	Others
Isaria farinosa KCh KW1.1	1	100 ± 0,0	0 ± 0,0	0 ± 0,0	0 ± 0,0	0 ± 0,0	0 ± 0,0	0 ± 0,0	0 ± 0,0
	3	64,53 ± 7,59	1,96 ± 0,58	0,21 ± 0,06	27,06 ± 5,31	1,35 ± 0,21	0 ± 0,0	0,39 ± 0,13	4,51 ± 1,87
	7	7,06 ± 2,10	1,68 ± 0,40	0,54 ± 0,26	72,40 ± 2,47	5,48 ± 0,92	0 ± 0,0	4,95 ± 0,30	8,02 ± 0,71
	10	0,78 ± 0,14	1,59 ± 0,34	0,42 ± 0,13	67,50 ± 1,25	5,12 ± 0,58	0 ± 0,0	15,02 ± 1,32	9,57 ± 0,42
Beauveria bassiana KCh BBT	1	46,34 ± 4,15	0,54 ± 0,18	0,24 ± 0,04	40,66 ± 6,31	11,74 ± 3,7	0 ± 0,0	0,47 ± 0,11	0 ± 0,0
	3	14,52 ± 7,05	1,01 ± 0,26	0,39 ± 0,02	66,17 ± 3,23	13,20 ± 3,42	0 ± 0,0	1,96 ± 0,26	2,75 ± 0,27
	7	10,13 ± 5,19	1,31 ± 0,40	0,41 ± 0,02	69,14 ± 2,69	11,92 ± 2,60	0 ± 0,0	3,88 ± 0,38	3,21 ± 0,17
	10	9,98 ± 4,28	1,65 ± 0,41	0,40 ± 0,05	66,53 ± 1,45	11,01 ± 2,78	0 ± 0,0	6,42 ± 0,46	4,00 ± 0,11
Metarhizium robertsii MU4	1	68,74 ± 4,66	3,42 ± 0,27	0,32 ± 0,10	22,29 ± 6,25	0 ± 0,0	0 ± 0,0	3,41 ± 4,37	1,82 ± 0,31
	3	6,91 ± 1,50	6,74 ± 0,54	0,91 ±0,05	74,27 ± 4,43	0 ± 0,0	0,31 ± 0,08	5,37 ± 3,25	5,49 ± 0,20
	7	2,82 ± 0,90	5,47 ± 0,74	1,15 ± 0,33	66,99 ± 6,23	0 ± 0,0	1,22 ± 0,51	13,24 ± 3,39	9,10 ± 2,58
	10	1,86 ± 0,30	3,31 ± 0,29	0,98 ± 0,31	65,74 ± 9,55	0 ± 0,0	1,35 ± 0,58	18,03 ± 6,61	8,74 ± 2,62
Beauveria bassiana KCh J1.5	1	100 ± 0,0	0 ± 0,0	0 ± 0,0	0 ± 0,0	0 ± 0,0	0 ± 0,0	0 ± 0,0	0 ± 0,0
	3	87,10 ± 6,83	0 ± 0,0	0 ± 0,0	11,20 ± 6,07	0 ± 0,0	0 ± 0,0	0 ± 0,0	1,70 ± 0,77
	7	20,76 ± 9,95	0 ± 0,0	0,58 ± 0,13	67,56 ± 6,92	0 ± 0,0	0 ± 0,0	3,05 ± 1,17	8,74 ± 2,62
	10	1,83 ± 0,49	0 ± 0,0	0,88 ± 0,03	79,04 ± 3,44	0 ± 0,0	0 ± 0,0	8,74 ± 1,45	7,10 ± 2,48
Isaria fumosorosea KCh J2	1	81,82 ± 1,96	0,53 ± 0,11	0,32 ± 0,07	14,80 ± 1,94	0 ± 0,0	0 ± 0,0	0 ± 0,0	2,54 ± 0,22
	3	35,08 ± 9,01	0,81 ± 0,17	0,93 ± 0,02	58,61 ± 8,67	0 ± 0,0	0 ± 0,0	1,73 ± 0,04	2,91 ± 0,31
	7	2,42 ± 1,88	0,26 ± 0,10	0,93 ± 0,05	89,11 ± 2,91	0 ± 0,0	0 ± 0,0	4,56 ± 1,00	2,88 ± 0,09
	10	1,95 ± 0,67	0,24 ± 0,06	0,92 ± 0,07	86,44 ± 1,61	0 ± 0,0	0 ± 0,0	7,67 ± 1,28	2,82 ± 0,36
Beauveria caledonica KCh J3.3	1	80,20 ± 4,74	0,58 ± 0,16	0 ± 0,0	17,12 ± 4,21	0 ± 0,0	0 ± 0,0	0 ± 0,0	2,10 ± 0,39
	3	14,24 ± 5,86	0,83 ± 0,13	0,73 ± 0,12	76,42 ± 4,37	0 ± 0,0	0 ± 0,0	3,48 ± 0,12	4,29 ± 1,23
	7	3,89 ± 1,48	0,62 ± 0,06	0,74 ± 0,04	83,57 ± 3,04	0 ± 0,0	0 ± 0,0	5,76 ± 1,30	5,42 ± 0,44
	10	1,91 ± 0,52	0,55 0,04	0,69 ± 0,0	86,80 ± 2,51	0 ± 0,0	0 ± 0,0	6,39 ± 2,11	3,66 ± 0,69
Isaria tenuipes MU35	1	70,62 ± 4,63	2,11 ± 0,70	0,34 ± 0,06	23,77 ± 3,01	0 ± 0,0	0 ± 0,0	0,39 ± 0,07	2,76 ± 1,26
	3	11,58 ± 6,64	4,73 ± 1,47	0,72 ± 0,06	67,33 ± 4,54	5,90 ± 0,0	0 ± 0,0	2,16 ± 0,25	7,58 ± 1,53
	7	8,46 ± 2,57	2,60 ± 0,54	0,59 ± 0,07	67,32 ± 1,58	8,08 ± 1,97	0 ± 0,0	4,50 ± 3,35	8,45 ± 1,27
	10	12,31 ± 2,32	2,24 ± 0,39	0,53 ± 0,04	65,54 ± 1,18	7,54 ± 0,88	0 ± 0,0	1,91 ± 0,09	10,0 1 ± 0,67

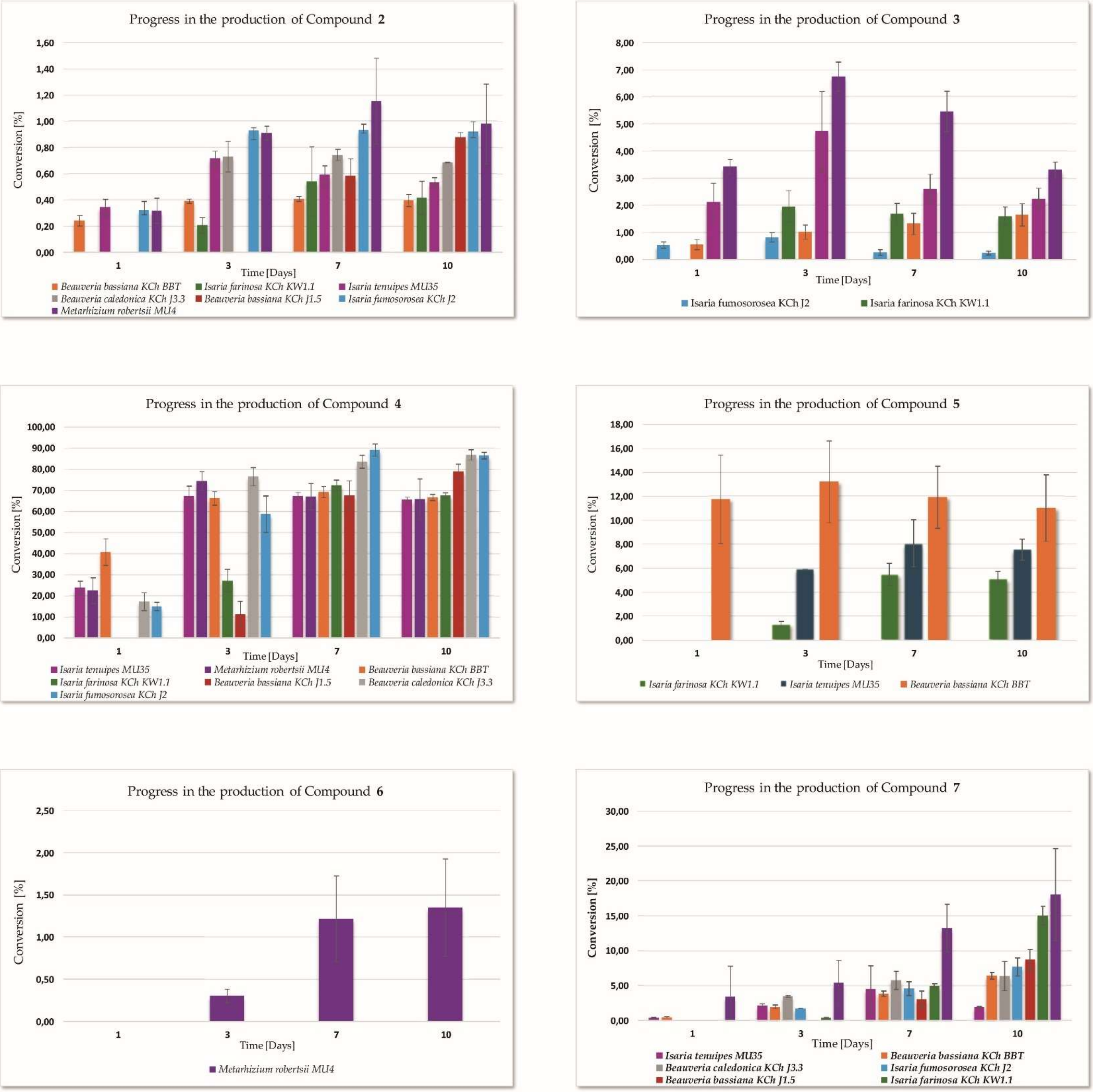

Figure S13. Progress in the production of metabolites **2-7**

Statistical analysis

ANOVA: Single Factor

DESCRIPTION Alpha 0,05

Group	Count	Sum	Mean	Variance	SS	Std Err	Lower	Upper
Isaria farinosa KCh KW1.1	3	300	100	0	0	2,489061	94,6615	105,3385
Beauveria bassiana KCh BBT	3	139,05	46,35	25,8335	51,667	2,489061	41,01054	51,68755
Metarhizium robertsii MU4	3	206,21	68,74	32,63746	65,27492	2,489061	63,39681	74,07382
Beauveria bassiana KCh J1.5	3	300	100	0	0	2,489061	94,6615	105,3385
Isaria fumosorosea KCh J2	3	245,46	81,82	5,784256	11,56851	2,489061	76,4801	87,15711
Beauveria caledonica KCh J3.3	3	240,59	80,20	33,75516	67,51033	2,489061	74,8579	85,53491
Isaria tenuipes MU35	3	211,85	70,62	32,09354	64,18708	2,489061	65,27757	75,95458

ANOVA

Sources	SS	df	MS	F	P value	Eta-sq	RMSSE	Omega Sq
Between Groups	6387,376	6	1064,563	57,27682	4,73E-09	0,960857	4,369471	0,941449
Within Groups	260,2078	14	18,58627					
Total	6647,584	20	332,3792					

Q TEST

group 1	*group 2*	*mean*	*std err*	*q-stat*	*lower*	*upper*	*p-value*	*mean-crit*	*Cohen d*
Isaria farinosa KCh KW1.1	*Beauveria bassiana KCh BBT*	53,651	2,489	21,555	41,631	65,671	7,14801E-09	12,020	12,445
Isaria farinosa KCh KW1.1	*Metarhizium robertsii MU4*	31,265	2,489	12,561	19,245	43,284	6,59943E-06	12,020	7,252
Isaria farinosa KCh KW1.1	*Beauveria bassiana KCh J1.5*	0,000	2,489	0,000	-12,020	12,020	1	12,020	0,000
Isaria farinosa KCh KW1.1	*Isaria fumosorosea KCh J2*	18,181	2,489	7,305	6,162	30,201	0,00212065	12,020	4,217
Isaria farinosa KCh KW1.1	*Beauveria caledonica KCh J3.3*	19,804	2,489	7,956	7,784	31,823	0,000949991	12,020	4,594
Isaria farinosa KCh KW1.1	*Isaria tenuipes MU35*	29,384	2,489	11,805	17,364	41,404	1,371E-05	12,020	6,816
Beauveria bassiana KCh BBT	*Metarhizium robertsii MU4*	22,386	2,489	8,994	10,367	34,406	0,000277717	12,020	5,193
Beauveria bassiana KCh BBT	*Beauveria bassiana KCh J1.5*	53,651	2,489	21,555	41,631	65,671	7,14801E-09	12,020	12,445
Beauveria bassiana KCh BBT	*Isaria fumosorosea KCh J2*	35,470	2,489	14,250	23,450	47,489	1,43091E-06	12,020	8,227
Beauveria bassiana KCh BBT	*Beauveria caledonica KCh J3.3*	33,847	2,489	13,598	21,828	45,867	2,53832E-06	12,020	7,851
Beauveria bassiana KCh BBT	*Isaria tenuipes MU35*	24,267	2,489	9,749	12,247	36,287	0,000118098	12,020	5,629
Metarhizium robertsii MU4	*Beauveria bassiana KCh J1.5*	31,265	2,489	12,561	19,245	43,284	6,59943E-06	12,020	7,252
Metarhizium robertsii MU4	*Isaria fumosorosea KCh J2*	13,083	2,489	5,256	1,064	25,103	0,029047375	12,020	3,035
Metarhizium robertsii MU4	*Beauveria caledonica KCh J3.3*	11,461	2,489	4,605	-0,559	23,481	0,066187242	12,020	2,658
Metarhizium robertsii MU4	*Isaria tenuipes MU35*	1,881	2,489	0,756	-10,139	13,900	0,997739686	12,020	0,436
Beauveria bassiana KCh J1.5	*Isaria fumosorosea KCh J2*	18,181	2,489	7,305	6,162	30,201	0,00212065	12,020	4,217
Beauveria bassiana KCh J1.5	*Beauveria caledonica KCh J3.3*	19,804	2,489	7,956	7,784	31,823	0,000949991	12,020	4,594
Beauveria bassiana KCh J1.5	*Isaria tenuipes MU35*	29,384	2,489	11,805	17,364	41,404	1,371E-05	12,020	6,816
Isaria fumosorosea KCh J2	*Beauveria caledonica KCh J3.3*	1,622	2,489	0,652	-10,397	13,642	0,999007857	12,020	0,376
Isaria fumosorosea KCh J2	*Isaria tenuipes MU35*	11,203	2,489	4,501	-0,817	23,222	0,075251448	12,020	2,598
Beauveria caledonica KCh J3.3	*Isaria tenuipes MU35*	9,580	2,489	3,849	-2,439	21,600	0,163173002	12,020	2,222

Day 1 Compound 1 conversion statistics

ANOVA: Single Factor

DESCRIPTION					Alpha	0,05		
Group	*Count*	*Sum*	*Mean*	*Variance*	*SS*	*Std Err*	*Lower*	*Upper*
Isaria farinosa KCh KW1.1	3	193,60	64,53	86,30884	172,6177	4,748403	54,3486	74,71723
Beauveria bassiana KCh BBT	3	43,58	14,53	74,51205	149,0241	4,748403	4,342317	24,71094
Metarhizium robertsii MU4	3	20,72	6,91	3,363162	6,726324	4,748403	-3,27617	17,09246
Beauveria bassiana KCh J1.5	3	261,30	87,10	70,00757	140,0151	4,748403	76,91597	97,28459
Isaria fumosorosea KCh J2	3	105,24	35,08	121,7954	243,5909	4,748403	24,89487	45,26349
Beauveria caledonica KCh J3.3	3	42,71	14,24	51,45825	102,9165	4,748403	4,051997	24,42062
Isaria tenuipes MU35	3	34,73	11,58	66,04869	132,0974	4,748403	1,392365	21,76099

ANOVA								
Sources	*SS*	*df*	*MS*	*F*	*P value*	*Eta-sq*	*RMSSE*	*Omega Sq*
Between Groups	17271,96	6	2878,661	42,5573	3,36E-08	0,948022	3,7664	0,922321
Within Groups	946,988	14	67,642					
Total	18218,95	20	910,9476					

Q TEST

group 1	*group 2*	*mean*	*std err*	*q-stat*	*lower*	*upper*	*p-value*	*mean-crit*	*Cohen d*
Isaria farinosa KCh KW1.1	*Beauveria bassiana KCh BBT*	50,006	4,748	10,531	27,076	72,936	5,05687E-05	22,930	6,080
Isaria farinosa KCh KW1.1	*Metarhizium robertsii MU4*	57,625	4,748	12,136	34,695	80,555	9,92088E-06	22,930	7,006
Isaria farinosa KCh KW1.1	*Beauveria bassiana KCh J1.5*	22,567	4,748	4,753	-0,363	45,497	0,055030544	22,930	2,744
Isaria farinosa KCh KW1.1	*Isaria fumosorosea KCh J2*	29,454	4,748	6,203	6,524	52,384	0,008589552	22,930	3,581
Isaria farinosa KCh KW1.1	*Beauveria caledonica KCh J3.3*	50,297	4,748	10,592	27,367	73,227	4,73961E-05	22,930	6,115
Isaria farinosa KCh KW1.1	*Isaria tenuipes MU35*	52,956	4,748	11,152	30,026	75,886	2,64485E-05	22,930	6,439
Beauveria bassiana KCh BBT	*Metarhizium robertsii MU4*	7,618	4,748	1,604	-15,312	30,549	0,906693358	22,930	0,926
Beauveria bassiana KCh BBT	*Beauveria bassiana KCh J1.5*	72,574	4,748	15,284	49,644	95,504	5,99788E-07	22,930	8,824
Beauveria bassiana KCh BBT	*Isaria fumosorosea KCh J2*	20,553	4,748	4,328	-2,377	43,483	0,092878748	22,930	2,499
Beauveria bassiana KCh BBT	*Beauveria caledonica KCh J3.3*	0,290	4,748	0,061	-22,640	23,220	0,999999999	22,930	0,035
Beauveria bassiana KCh BBT	*Isaria tenuipes MU35*	2,950	4,748	0,621	-19,980	25,880	0,999242794	22,930	0,359
Metarhizium robertsii MU4	*Beauveria bassiana KCh J1.5*	80,192	4,748	16,888	57,262	103,122	1,69788E-07	22,930	9,750
Metarhizium robertsii MU4	*Isaria fumosorosea KCh J2*	28,171	4,748	5,933	5,241	51,101	0,012162887	22,930	3,425
Metarhizium robertsii MU4	*Beauveria caledonica KCh J3.3*	7,328	4,748	1,543	-15,602	30,258	0,920667313	22,930	0,891
Metarhizium robertsii MU4	*Isaria tenuipes MU35*	4,669	4,748	0,983	-18,262	27,599	0,990735189	22,930	0,568
Beauveria bassiana KCh J1.5	*Isaria fumosorosea KCh J2*	52,021	4,748	10,955	29,091	74,951	3,24018E-05	22,930	6,325
Beauveria bassiana KCh J1.5	*Beauveria caledonica KCh J3.3*	72,864	4,748	15,345	49,934	95,794	5,70545E-07	22,930	8,859
Beauveria bassiana KCh J1.5	*Isaria tenuipes MU35*	75,524	4,748	15,905	52,594	98,454	3,63527E-07	22,930	9,183
Isaria fumosorosea KCh J2	*Beauveria caledonica KCh J3.3*	20,843	4,748	4,389	-2,087	43,773	0,086232421	22,930	2,534
Isaria fumosorosea KCh J2	*Isaria tenuipes MU35*	23,503	4,748	4,950	0,572	46,433	0,042937524	22,930	2,858
Beauveria caledonica KCh J3.3	*Isaria tenuipes MU35*	2,660	4,748	0,560	-20,270	25,590	0,999580071	22,930	0,323

Day 3 Compound 1 conversion statistic

ANOVA: Single Factor

DESCRIPTION Alpha 0,05

Group	Count	Sum	Mean	Variance	SS	Std Err	Lower	Upper
Isaria farinosa KCh KW1.1	3	21,18	7,06	6,607389	13,21478	3,201363	0,194283	13,92677
Beauveria bassiana KCh BBT	3	30,39	10,13	40,48102	80,96204	3,201363	3,264253	16,99674
Metarhizium robertsii MU4	3	8,47	2,82	1,208119	2,416239	3,201363	-4,04457	9,687915
Beauveria bassiana KCh J1.5	3	62,28	20,76	148,452	296,904	3,201363	13,89287	27,62535
Isaria fumosorosea KCh J2	3	7,24	2,41	5,31597	10,63194	3,201363	-4,45435	9,278132
Beauveria caledonica KCh J3.3	3	11,67	3,89	3,26597	6,531941	3,201363	-2,97721	10,75527
Isaria tenuipes MU35	3	25,39	8,46	9,89279	19,78558	3,201363	1,597329	15,32981

ANOVA

Sources	*SS*	*df*	*MS*	*F*	*P value*	*Eta-sq*	*RMSSE*	*Omega Sq*
Between Groups	730,0287	6	121,6714	3,957287	0,015924	0,629077	1,148519	0,457977
Within Groups	430,4465	14	30,74618					
Total	1160,475	20	58,02376					

Q TEST

group 1	group 2	mean	std err	q-stat	lower	upper	p-value	mean-crit	Cohen d
Isaria farinosa KCh KW1.1	*Beauveria bassiana KCh BBT*	3,070	3,201	0,959	-12,389	18,529	0,991863914	15,459	0,554
Isaria farinosa KCh KW1.1	*Metarhizium robertsii MU4*	4,239	3,201	1,324	-11,221	19,698	0,959798373	15,459	0,764
Isaria farinosa KCh KW1.1	*Beauveria bassiana KCh J1.5*	13,699	3,201	4,279	-1,761	29,158	0,098578375	15,459	2,470
Isaria farinosa KCh KW1.1	*Isaria fumosorosea KCh J2*	4,649	3,201	1,452	-10,811	20,108	0,939012021	15,459	0,838
Isaria farinosa KCh KW1.1	*Beauveria caledonica KCh J3.3*	3,171	3,201	0,991	-12,288	18,631	0,990363351	15,459	0,572
Isaria farinosa KCh KW1.1	*Isaria tenuipes MU35*	1,403	3,201	0,438	-14,056	16,862	0,999898229	15,459	0,253
Beauveria bassiana KCh BBT	*Metarhizium robertsii MU4*	7,309	3,201	2,283	-8,151	22,768	0,676476196	15,459	1,318
Beauveria bassiana KCh BBT	*Beauveria bassiana KCh J1.5*	10,629	3,201	3,320	-4,831	26,088	0,288286946	15,459	1,917
Beauveria bassiana KCh BBT	*Isaria fumosorosea KCh J2*	7,719	3,201	2,411	-7,741	23,178	0,623905792	15,459	1,392
Beauveria bassiana KCh BBT	*Beauveria caledonica KCh J3.3*	6,241	3,201	1,950	-9,218	21,701	0,80407914	15,459	1,126
Beauveria bassiana KCh BBT	*Isaria tenuipes MU35*	1,667	3,201	0,521	-13,792	17,126	0,999723768	15,459	0,301
Metarhizium robertsii MU4	*Beauveria bassiana KCh J1.5*	17,937	3,201	5,603	2,478	33,397	0,018603742	15,459	3,235
Metarhizium robertsii MU4	*Isaria fumosorosea KCh J2*	0,410	3,201	0,128	-15,050	15,869	0,999999932	15,459	0,074
Metarhizium robertsii MU4	*Beauveria caledonica KCh J3.3*	1,067	3,201	0,333	-14,392	16,527	0,999979538	15,459	0,192
Metarhizium robertsii MU4	*Isaria tenuipes MU35*	5,642	3,201	1,762	-9,817	21,101	0,864506582	15,459	1,017
Beauveria bassiana KCh J1.5	*Isaria fumosorosea KCh J2*	18,347	3,201	5,731	2,888	33,807	0,015774549	15,459	3,309
Beauveria bassiana KCh J1.5	*Beauveria caledonica KCh J3.3*	16,870	3,201	5,270	1,411	32,329	0,028555213	15,459	3,042
Beauveria bassiana KCh J1.5	*Isaria tenuipes MU35*	12,296	3,201	3,841	-3,164	27,755	0,164707651	15,459	2,217
Isaria fumosorosea KCh J2	*Beauveria caledonica KCh J3.3*	1,477	3,201	0,461	-13,982	16,937	0,999862708	15,459	0,266
Isaria fumosorosea KCh J2	*Isaria tenuipes MU35*	6,052	3,201	1,890	-9,408	21,511	0,82430924	15,459	1,091
Beauveria caledonica KCh J3.3	*Isaria tenuipes MU35*	4,575	3,201	1,429	-10,885	20,034	0,943193163	15,459	0,825

Day 7 Compound 1 conversion statistics

ANOVA: Single Factor

DESCRIPTION					Alpha	0,05		
Group	*Count*	*Sum*	*Mean*	*Variance*	*SS*	*Std Err*	*Lower*	*Upper*
Isaria farinosa KCh KW1.1	3	2,35	0,78	0,029	0,058	1,329	-2,066	3,635
Beauveria bassiana KCh BBT	3	29,97	9,99	27,438	54,876	1,329	7,138	12,839
Metarhizium robertsii MU4	3	5,57	1,86	0,131	0,263	1,329	-0,995	4,706
Beauveria bassiana KCh J1.5	3	5,49	1,83	0,354	0,709	1,329	-1,021	4,680
Isaria fumosorosea KCh J2	3	5,85	1,95	0,678	1,355	1,329	-0,901	4,800
Beauveria caledonica KCh J3.3	3	5,74	1,91	0,408	0,816	1,329	-0,937	4,764
Isaria tenuipes MU35	3	36,92	12,31	8,056	16,112	1,329	9,458	15,159

ANOVA								
Sources	*SS*	*df*	*MS*	*F*	*P value*	*Eta-sq*	*RMSSE*	*Omega Sq*
Between Groups	396,3074	6	66,05124	12,46458	6,49E-05	0,84232	2,038347	0,766114
Within Groups	74,18762	14	5,299116					
Total	470,4951	20	23,52475					

Q TEST

group 1	*group 2*	*mean*	*std err*	*q-stat*	*lower*	*upper*	*p-value*	*mean-crit*	*Cohen d*
Isaria farinosa KCh KW1.1	*Beauveria bassiana KCh BBT*	9,204	1,329	6,925	2,786	15,622	0,003417	6,418	3,998
Isaria farinosa KCh KW1.1	*Metarhizium robertsii MU4*	1,071	1,329	0,806	-5,347	7,489	0,996788	6,418	0,465
Isaria farinosa KCh KW1.1	*Beauveria bassiana KCh J1.5*	1,045	1,329	0,786	-5,373	7,463	0,997188	6,418	0,454
Isaria farinosa KCh KW1.1	*Isaria fumosorosea KCh J2*	1,165	1,329	0,876	-5,253	7,583	0,994944	6,418	0,506
Isaria farinosa KCh KW1.1	*Beauveria caledonica KCh J3.3*	1,129	1,329	0,849	-5,289	7,547	0,995726	6,418	0,490
Isaria farinosa KCh KW1.1	*Isaria tenuipes MU35*	11,523	1,329	8,670	5,105	17,941	0,000405	6,418	5,006
Beauveria bassiana KCh BBT	*Metarhizium robertsii MU4*	8,133	1,329	6,119	1,715	14,551	0,009564	6,418	3,533
Beauveria bassiana KCh BBT	*Beauveria bassiana KCh J1.5*	8,159	1,329	6,139	1,741	14,577	0,009329	6,418	3,544
Beauveria bassiana KCh BBT	*Isaria fumosorosea KCh J2*	8,039	1,329	6,049	1,621	14,457	0,010476	6,418	3,492
Beauveria bassiana KCh BBT	*Beauveria caledonica KCh J3.3*	8,075	1,329	6,076	1,657	14,493	0,010118	6,418	3,508
Beauveria bassiana KCh BBT	*Isaria tenuipes MU35*	2,320	1,329	1,745	-4,098	8,738	0,869439	6,418	1,008
Metarhizium robertsii MU4	*Beauveria bassiana KCh J1.5*	0,026	1,329	0,019	-6,392	6,444	1	6,418	0,011
Metarhizium robertsii MU4	*Isaria fumosorosea KCh J2*	0,094	1,329	0,071	-6,324	6,512	1	6,418	0,041
Metarhizium robertsii MU4	*Beauveria caledonica KCh J3.3*	0,058	1,329	0,044	-6,360	6,476	1	6,418	0,025
Metarhizium robertsii MU4	*Isaria tenuipes MU35*	10,453	1,329	7,865	4,035	16,871	0,001062	6,418	4,541
Beauveria bassiana KCh J1.5	*Isaria fumosorosea KCh J2*	0,120	1,329	0,090	-6,298	6,538	1	6,418	0,052
Beauveria bassiana KCh J1.5	*Beauveria caledonica KCh J3.3*	0,084	1,329	0,063	-6,334	6,502	1	6,418	0,036
Beauveria bassiana KCh J1.5	*Isaria tenuipes MU35*	10,478	1,329	7,884	4,060	16,896	0,001037	6,418	4,552
Isaria fumosorosea KCh J2	*Beauveria caledonica KCh J3.3*	0,036	1,329	0,027	-6,382	6,454	1	6,418	0,016
Isaria fumosorosea KCh J2	*Isaria tenuipes MU35*	10,359	1,329	7,794	3,941	16,777	0,001158	6,418	4,500
Beauveria caledonica KCh J3.3	*Isaria tenuipes MU35*	10,395	1,329	7,821	3,977	16,813	0,00112	6,418	4,515

Day 10 Compound 1 conversion statistics

ANOVA: Single Factor

DESCRIPTION					Alpha	0,05		
Group	*Count*	*Sum*	*Mean*	*Variance*	*SS*	*Std Err*	*Lower*	*Upper*
Isaria farinosa KCh KW1.1	3	0	0	0	0	0,036979	-0,07931	0,07931121
Beauveria bassiana KCh BBT	3	0,72	0,24	0,002398	0,004797	0,036979	0,162214	0,32083613
Metarhizium robertsii MU4	3	0,95	0,32	0,013847	0,027695	0,036979	0,237877	0,39649983
Beauveria bassiana KCh J1.5	3	0	0	0	0	0,036979	-0,07931	0,07931121
Isaria fumosorosea KCh J2	3	0,96	0,32	0,007118	0,014236	0,036979	0,242279	0,4009019
Beauveria caledonica KCh J3.3	3	0	0	0	0	0,036979	-0,07931	0,07931121
Isaria tenuipes MU35	3	1,03	0,34	0,005352	0,010704	0,036979	0,264898	0,42352091

ANOVA								
Sources	*SS*	*df*	*MS*	*F*	*P value*	*Eta-sq*	*RMSSE*	*Omega Sq*
Between Groups	0,499917	6	0,083319	20,31066	3,68E-06	0,896956	2,601965	0,84656299
Within Groups	0,057432	14	0,004102					
Total	0,557348	20	0,027867					

Q TEST

group 1	group 2	mean	std err	q-stat	lower	upper	p-value	mean-crit	Cohen d
Isaria farinosa KCh KW1.1	*Beauveria bassiana KCh BBT*	0,242	0,037	6,531	0,063	0,420	0,005636	0,179	3,771
Isaria farinosa KCh KW1.1	*Metarhizium robertsii MU4*	0,317	0,037	8,578	0,139	0,496	0,000451	0,179	4,952
Isaria farinosa KCh KW1.1	*Beauveria bassiana KCh J1.5*	0,000	0,037	0,000	-0,179	0,179	1	0,179	0,000
Isaria farinosa KCh KW1.1	*Isaria fumosorosea KCh J2*	0,322	0,037	8,697	0,143	0,500	0,000392	0,179	5,021
Isaria farinosa KCh KW1.1	*Beauveria caledonica KCh J3.3*	0,000	0,037	0,000	-0,179	0,179	1	0,179	0,000
Isaria farinosa KCh KW1.1	*Isaria tenuipes MU35*	0,344	0,037	9,308	0,166	0,523	0,000194	0,179	5,374
Beauveria bassiana KCh BBT	*Metarhizium robertsii MU4*	0,076	0,037	2,046	-0,103	0,254	0,769258	0,179	1,181
Beauveria bassiana KCh BBT	*Beauveria bassiana KCh J1.5*	0,242	0,037	6,531	0,063	0,420	0,005636	0,179	3,771
Beauveria bassiana KCh BBT	*Isaria fumosorosea KCh J2*	0,080	0,037	2,165	-0,099	0,259	0,72367	0,179	1,250
Beauveria bassiana KCh BBT	*Beauveria caledonica KCh J3.3*	0,242	0,037	6,531	0,063	0,420	0,005636	0,179	3,771
Beauveria bassiana KCh BBT	*Isaria tenuipes MU35*	0,103	0,037	2,777	-0,076	0,281	0,475213	0,179	1,603
Metarhizium robertsii MU4	*Beauveria bassiana KCh J1.5*	0,317	0,037	8,578	0,139	0,496	0,000451	0,179	4,952
Metarhizium robertsii MU4	*Isaria fumosorosea KCh J2*	0,004	0,037	0,119	-0,174	0,183	1	0,179	0,069
Metarhizium robertsii MU4	*Beauveria caledonica KCh J3.3*	0,317	0,037	8,578	0,139	0,496	0,000451	0,179	4,952
Metarhizium robertsii MU4	*Isaria tenuipes MU35*	0,027	0,037	0,731	-0,152	0,206	0,998121	0,179	0,422
Beauveria bassiana KCh J1.5	*Isaria fumosorosea KCh J2*	0,322	0,037	8,697	0,143	0,500	0,000392	0,179	5,021
Beauveria bassiana KCh J1.5	*Beauveria caledonica KCh J3.3*	0,000	0,037	0,000	-0,179	0,179	1	0,179	0,000
Beauveria bassiana KCh J1.5	*Isaria tenuipes MU35*	0,344	0,037	9,308	0,166	0,523	0,000194	0,179	5,374
Isaria fumosorosea KCh J2	*Beauveria caledonica KCh J3.3*	0,322	0,037	8,697	0,143	0,500	0,000392	0,179	5,021
Isaria fumosorosea KCh J2	*Isaria tenuipes MU35*	0,023	0,037	0,612	-0,156	0,201	0,999307	0,179	0,353
Beauveria caledonica KCh J3.3	*Isaria tenuipes MU35*	0,344	0,037	9,308	0,166	0,523	0,000194	0,179	5,374

Day 1 Compound 2 conversion statistics

ANOVA: Single Factor

DESCRIPTION Alpha 0,05

Group	*Count*	*Sum*	*Mean*	*Variance*	*SS*	*Std Err*	*Lower*	*Upper*
Isaria farinosa KCh KW1.1	3	0,50	0,17	0,020515	0,041031	0,049204	0,059639	0,270701
Beauveria bassiana KCh BBT	3	1,17	0,39	0,00045	0,0009	0,049204	0,284718	0,495781
Metarhizium robertsii MU4	3	2,73	0,91	0,00416	0,008319	0,049204	0,804176	1,015239
Beauveria bassiana KCh J1.5	3	0	0	0	0	0,049204	-0,10553	0,105531
Isaria fumosorosea KCh J2	3	2,79	0,93	0,00071	0,001421	0,049204	0,824198	1,035261
Beauveria caledonica KCh J3.3	3	2,19	0,73	0,020353	0,040707	0,049204	0,623886	0,834949
Isaria tenuipes MU35	3	2,15	0,72	0,004652	0,009304	0,049204	0,61013	0,821193

ANOVA

Sources	*SS*	*df*	*MS*	*F*	*P value*	*Eta-sq*	*RMSSE*	*Omega Sq*
Between Groups	2,427955	6	0,404659	55,7152	5,68E-09	0,959804	4,309494	0,939878
Within Groups	0,101682	14	0,007263					
Total	2,529637	20	0,126482					

Q TEST

group 1	group 2	mean	std err	q-stat	lower	upper	p-value	mean-crit	Cohen d
Isaria farinosa KCh KW1.1	*Beauveria bassiana KCh BBT*	0,225	0,049	4,574	-0,013	0,463	0,068705654	0,238	2,641
Isaria farinosa KCh KW1.1	*Metarhizium robertsii MU4*	0,745	0,049	15,132	0,507	0,982	6,79637E-07	0,238	8,736
Isaria farinosa KCh KW1.1	*Beauveria bassiana KCh J1.5*	0,165	0,049	3,357	-0,072	0,403	0,277718535	0,238	1,938
Isaria farinosa KCh KW1.1	*Isaria fumosorosea KCh J2*	0,765	0,049	15,539	0,527	1,002	4,87473E-07	0,238	8,971
Isaria farinosa KCh KW1.1	*Beauveria caledonica KCh J3.3*	0,564	0,049	11,468	0,327	0,802	1,9201E-05	0,238	6,621
Isaria farinosa KCh KW1.1	*Isaria tenuipes MU35*	0,550	0,049	11,188	0,313	0,788	2,55018E-05	0,238	6,459
Beauveria bassiana KCh BBT	*Metarhizium robertsii MU4*	0,519	0,049	10,557	0,282	0,757	4,91861E-05	0,238	6,095
Beauveria bassiana KCh BBT	*Beauveria bassiana KCh J1.5*	0,390	0,049	7,931	0,153	0,628	0,000979229	0,238	4,579
Beauveria bassiana KCh BBT	*Isaria fumosorosea KCh J2*	0,539	0,049	10,964	0,302	0,777	3,21098E-05	0,238	6,330
Beauveria bassiana KCh BBT	*Beauveria caledonica KCh J3.3*	0,339	0,049	6,893	0,102	0,577	0,003557305	0,238	3,980
Beauveria bassiana KCh BBT	*Isaria tenuipes MU35*	0,325	0,049	6,614	0,088	0,563	0,005075059	0,238	3,818
Metarhizium robertsii MU4	*Beauveria bassiana KCh J1.5*	0,910	0,049	18,489	0,672	1,147	5,29983E-08	0,238	10,674
Metarhizium robertsii MU4	*Isaria fumosorosea KCh J2*	0,020	0,049	0,407	-0,218	0,258	0,999934019	0,238	0,235
Metarhizium robertsii MU4	*Beauveria caledonica KCh J3.3*	0,180	0,049	3,664	-0,057	0,418	0,200559111	0,238	2,116
Metarhizium robertsii MU4	*Isaria tenuipes MU35*	0,194	0,049	3,944	-0,044	0,432	0,146408038	0,238	2,277
Beauveria bassiana KCh J1.5	*Isaria fumosorosea KCh J2*	0,930	0,049	18,896	0,692	1,167	3,99649E-08	0,238	10,909
Beauveria bassiana KCh J1.5	*Beauveria caledonica KCh J3.3*	0,729	0,049	14,824	0,492	0,967	8,77597E-07	0,238	8,559
Beauveria bassiana KCh J1.5	*Isaria tenuipes MU35*	0,716	0,049	14,545	0,478	0,953	1,11136E-06	0,238	8,397
Isaria fumosorosea KCh J2	*Beauveria caledonica KCh J3.3*	0,200	0,049	4,071	-0,037	0,438	0,126243176	0,238	2,350
Isaria fumosorosea KCh J2	*Isaria tenuipes MU35*	0,214	0,049	4,351	-0,024	0,452	0,090396882	0,238	2,512
Beauveria caledonica KCh J3.3	*Isaria tenuipes MU35*	0,014	0,049	0,280	-0,224	0,251	0,99999278	0,238	0,161

Day 3 Compound 2 conversion statistics

ANOVA: Single Factor

DESCRIPTION					Alpha	0,05		
Group	*Count*	*Sum*	*Mean*	*Variance*	*SS*	*Std Err*	*Lower*	*Upper*
Isaria farinosa KCh KW1.1	3	0,914485	0,304828	0,278761	0,557522	0,190987	-0,1048	0,714454
Beauveria bassiana KCh BBT	3	1,22	0,41	0,000503	0,001006	0,190987	-0,00149	0,817764
Metarhizium robertsii MU4	3	3,46	1,15	0,161399	0,322797	0,190987	0,744144	1,563395
Beauveria bassiana KCh J1.5	3	1,75	0,58	0,025204	0,050408	0,190987	0,173695	0,992946
Isaria fumosorosea KCh J2	3	1,86	0,62	0,290566	0,581132	0,190987	0,209449	1,0287
Beauveria caledonica KCh J3.3	3	2,23	0,74	0,002619	0,005238	0,190987	0,333204	1,152455
Isaria tenuipes MU35	3	1,77	0,59	0,006941	0,013883	0,190987	0,181842	1,001093

ANOVA								
Sources	*SS*	*df*	*MS*	*F*	*P value*	*Eta-sq*	*RMSSE*	*Omega Sq*
Between Groups	1,337404	6	0,222901	2,036969	0,12794	0,466093	0,824008	0,22856
Within Groups	1,531987	14	0,109428					
Total	2,869391	20	0,14347					

Q TEST

group 1	group 2	mean	std err	q-stat	lower	upper	p-value	mean-crit	Cohen d
Isaria farinosa KCh KW1.1	*Beauveria bassiana KCh BBT*	0,103	0,191	0,541	-0,819	1,026	0,999656	0,922	0,312
Isaria farinosa KCh KW1.1	*Metarhizium robertsii MU4*	0,849	0,191	4,445	-0,073	1,771	0,080573	0,922	2,566
Isaria farinosa KCh KW1.1	*Beauveria bassiana KCh J1.5*	0,278	0,191	1,458	-0,644	1,201	0,937879	0,922	0,842
Isaria farinosa KCh KW1.1	*Isaria fumosorosea KCh J2*	0,314	0,191	1,645	-0,608	1,237	0,896585	0,922	0,950
Isaria farinosa KCh KW1.1	*Beauveria caledonica KCh J3.3*	0,438	0,191	2,293	-0,484	1,360	0,672272	0,922	1,324
Isaria farinosa KCh KW1.1	*Isaria tenuipes MU35*	0,287	0,191	1,501	-0,636	1,209	0,929579	0,922	0,867
Beauveria bassiana KCh BBT	*Metarhizium robertsii MU4*	0,746	0,191	3,904	-0,177	1,668	0,15323	0,922	2,254
Beauveria bassiana KCh BBT	*Beauveria bassiana KCh J1.5*	0,175	0,191	0,917	-0,747	1,097	0,99356	0,922	0,530
Beauveria bassiana KCh BBT	*Isaria fumosorosea KCh J2*	0,211	0,191	1,104	-0,711	1,133	0,983236	0,922	0,638
Beauveria bassiana KCh BBT	*Beauveria caledonica KCh J3.3*	0,335	0,191	1,752	-0,588	1,257	0,867403	0,922	1,012
Beauveria bassiana KCh BBT	*Isaria tenuipes MU35*	0,183	0,191	0,960	-0,739	1,106	0,991822	0,922	0,554
Metarhizium robertsii MU4	*Beauveria bassiana KCh J1.5*	0,570	0,191	2,987	-0,352	1,493	0,396514	0,922	1,724
Metarhizium robertsii MU4	*Isaria fumosorosea KCh J2*	0,535	0,191	2,800	-0,388	1,457	0,466354	0,922	1,616
Metarhizium robertsii MU4	*Beauveria caledonica KCh J3.3*	0,411	0,191	2,152	-0,511	1,333	0,72897	0,922	1,242
Metarhizium robertsii MU4	*Isaria tenuipes MU35*	0,562	0,191	2,944	-0,360	1,485	0,411919	0,922	1,700
Beauveria bassiana KCh J1.5	*Isaria fumosorosea KCh J2*	0,036	0,191	0,187	-0,887	0,958	0,999999	0,922	0,108
Beauveria bassiana KCh J1.5	*Beauveria caledonica KCh J3.3*	0,160	0,191	0,835	-0,763	1,082	0,996098	0,922	0,482
Beauveria bassiana KCh J1.5	*Isaria tenuipes MU35*	0,008	0,191	0,043	-0,914	0,930	1	0,922	0,025
Isaria fumosorosea KCh J2	*Beauveria caledonica KCh J3.3*	0,124	0,191	0,648	-0,799	1,046	0,99904	0,922	0,374
Isaria fumosorosea KCh J2	*Isaria tenuipes MU35*	0,028	0,191	0,145	-0,895	0,950	1	0,922	0,083
Beauveria caledonica KCh J3.3	*Isaria tenuipes MU35*	0,151	0,191	0,793	-0,771	1,074	0,997064	0,922	0,458

Day 7 Compound 2 conversion statistics

ANOVA: Single Factor

DESCRIPTION Alpha 0,05

Group	Count	Sum	Mean	Variance	SS	Std Err	Lower	Upper
Isaria farinosa KCh KW1.1	3	0	0	0	0	0,085929	-0,1843	0,184299
Beauveria bassiana KCh BBT	3	1,19	0,40	0,003134	0,006268	0,085929	0,211779	0,580377
Metarhizium robertsii MU4	3	2,94	0,98	0,14008	0,28016	0,085929	0,794612	1,16321
Beauveria bassiana KCh J1.5	3	2,64	0,88	0,001748	0,003497	0,085929	0,695095	1,063693
Isaria fumosorosea KCh J2	3	2,76	0,92	0,008126	0,016252	0,085929	0,737217	1,105815
Beauveria caledonica KCh J3.3	3	2,06	0,69	4,28E-06	8,56E-06	0,085929	0,501968	0,870566
Isaria tenuipes MU35	3	1,60	0,53	0,001966	0,003931	0,085929	0,350522	0,71912

ANOVA

Sources	SS	df	MS	F	P value	Eta-sq	RMSSE	Omega Sq
Between Groups	2,198214	6	0,366369	16,53944	1,26E-05	0,876365	2,348009	0,816171
Within Groups	0,310117	14	0,022151					
Total	2,508331	20	0,125417					

Q TEST

group 1	*group 2*	*mean*	*std err*	*q-stat*	*lower*	*upper*	*p-value*	*mean-crit*	*Cohen d*
Isaria farinosa KCh KW1.1	*Beauveria bassiana KCh BBT*	0,396	0,086	4,609	-0,019	0,811	0,065794	0,415	2,661
Isaria farinosa KCh KW1.1	*Metarhizium robertsii MU4*	0,979	0,086	11,392	0,564	1,394	2,07E-05	0,415	6,577
Isaria farinosa KCh KW1.1	*Beauveria bassiana KCh J1.5*	0,879	0,086	10,234	0,464	1,294	6,95E-05	0,415	5,909
Isaria farinosa KCh KW1.1	*Isaria fumosorosea KCh J2*	0,922	0,086	10,724	0,507	1,336	4,12E-05	0,415	6,192
Isaria farinosa KCh KW1.1	*Beauveria caledonica KCh J3.3*	0,686	0,086	7,986	0,271	1,101	0,000916	0,415	4,611
Isaria farinosa KCh KW1.1	*Isaria tenuipes MU35*	0,535	0,086	6,224	0,120	0,950	0,008359	0,415	3,593
Beauveria bassiana KCh BBT	*Metarhizium robertsii MU4*	0,583	0,086	6,783	0,168	0,998	0,004092	0,415	3,916
Beauveria bassiana KCh BBT	*Beauveria bassiana KCh J1.5*	0,483	0,086	5,625	0,068	0,898	0,018094	0,415	3,247
Beauveria bassiana KCh BBT	*Isaria fumosorosea KCh J2*	0,525	0,086	6,115	0,110	0,940	0,00962	0,415	3,530
Beauveria bassiana KCh BBT	*Beauveria caledonica KCh J3.3*	0,290	0,086	3,377	-0,125	0,705	0,27204	0,415	1,950
Beauveria bassiana KCh BBT	*Isaria tenuipes MU35*	0,139	0,086	1,615	-0,276	0,554	0,904232	0,415	0,932
Metarhizium robertsii MU4	*Beauveria bassiana KCh J1.5*	0,100	0,086	1,158	-0,315	0,514	0,97879	0,415	0,669
Metarhizium robertsii MU4	*Isaria fumosorosea KCh J2*	0,057	0,086	0,668	-0,358	0,472	0,998861	0,415	0,386
Metarhizium robertsii MU4	*Beauveria caledonica KCh J3.3*	0,293	0,086	3,406	-0,122	0,708	0,264161	0,415	1,966
Metarhizium robertsii MU4	*Isaria tenuipes MU35*	0,444	0,086	5,168	0,029	0,859	0,032515	0,415	2,984
Beauveria bassiana KCh J1.5	*Isaria fumosorosea KCh J2*	0,042	0,086	0,490	-0,373	0,457	0,999805	0,415	0,283
Beauveria bassiana KCh J1.5	*Beauveria caledonica KCh J3.3*	0,193	0,086	2,248	-0,222	0,608	0,69086	0,415	1,298
Beauveria bassiana KCh J1.5	*Isaria tenuipes MU35*	0,345	0,086	4,010	-0,070	0,760	0,135592	0,415	2,315
Isaria fumosorosea KCh J2	*Beauveria caledonica KCh J3.3*	0,235	0,086	2,738	-0,180	0,650	0,490596	0,415	1,581
Isaria fumosorosea KCh J2	*Isaria tenuipes MU35*	0,387	0,086	4,500	-0,028	0,802	0,075299	0,415	2,598
Beauveria caledonica KCh J3.3	*Isaria tenuipes MU35*	0,151	0,086	1,762	-0,264	0,566	0,864472	0,415	1,018

Day 10 Compound 2 conversion statistics

ANOVA: Single Factor

DESCRIPTION					Alpha	0,05		
Group	*Count*	*Sum*	*Mean*	*Variance*	*SS*	*Std Err*	*Lower*	*Upper*
Isaria farinosa KCh KW1.1	3	0	0	0	0	0,230779	-0,50283	0,502825
Beauveria bassiana KCh BBT	3	1,63	0,54	0,051217	0,102434	0,230779	0,04034	1,04599
Metarhizium robertsii MU4	3	10,27	3,42	0,106567	0,213134	0,230779	2,920463	3,926113
Isaria fumosorosea KCh J2	3	1,59	0,53	0,019666	0,039332	0,230779	0,026032	1,031682
Beauveria caledonica KCh J3.3	3	1,75	0,58	0,03747	0,07494	0,230779	0,081305	1,086955
Isaria tenuipes MU35	3	6,34	2,11	0,743745	1,487489	0,230779	1,612043	2,617693

ANOVA

Sources	*SS*	*df*	*MS*	*F*	*P value*	*Eta-sq*	*RMSSE*	*Omega Sq*
Between Groups	25,44345	5	5,088689	31,84862	1,58E-06	0,929924	3,258252	0,895497
Within Groups	1,917328	12	0,159777					
Total	27,36078	17	1,609457					

Q TEST

group 1	*group 2*	*mean*	*std err*	*q-stat*	*lower*	*upper*	*p-value*	*mean-crit*	*Cohen d*
Isaria farinosa KCh KW1.1	*Beauveria bassiana KCh BBT*	0,543	0,231	2,354	-0,553	1,639	0,576545788	1,096	1,359
Isaria farinosa KCh KW1.1	*Metarhizium robertsii MU4*	3,423	0,231	14,834	2,327	4,519	2,51753E-06	1,096	8,564
Isaria farinosa KCh KW1.1	*Isaria fumosorosea KCh J2*	0,529	0,231	2,292	-0,567	1,625	0,6016993	1,096	1,323
Isaria farinosa KCh KW1.1	*Beauveria caledonica KCh J3.3*	0,584	0,231	2,531	-0,512	1,680	0,505752569	1,096	1,461
Isaria farinosa KCh KW1.1	*Isaria tenuipes MU35*	2,115	0,231	9,164	1,019	3,211	0,000336721	1,096	5,291
Beauveria bassiana KCh BBT	*Metarhizium robertsii MU4*	2,880	0,231	12,480	1,784	3,976	1,57944E-05	1,096	7,205
Beauveria bassiana KCh BBT	*Isaria fumosorosea KCh J2*	0,014	0,231	0,062	-1,082	1,111	0,99999997	1,096	0,036
Beauveria bassiana KCh BBT	*Beauveria caledonica KCh J3.3*	0,041	0,231	0,178	-1,055	1,137	0,999994286	1,096	0,102
Beauveria bassiana KCh BBT	*Isaria tenuipes MU35*	1,572	0,231	6,810	0,476	2,668	0,00436316	1,096	3,932
Metarhizium robertsii MU4	*Isaria fumosorosea KCh J2*	2,894	0,231	12,542	1,798	3,991	1,49999E-05	1,096	7,241
Metarhizium robertsii MU4	*Beauveria caledonica KCh J3.3*	2,839	0,231	12,302	1,743	3,935	1,83287E-05	1,096	7,103
Metarhizium robertsii MU4	*Isaria tenuipes MU35*	1,308	0,231	5,670	0,212	2,405	0,016700443	1,096	3,273
Isaria fumosorosea KCh J2	*Beauveria caledonica KCh J3.3*	0,055	0,231	0,240	-1,041	1,151	0,999974704	1,096	0,138
Isaria fumosorosea KCh J2	*Isaria tenuipes MU35*	1,586	0,231	6,872	0,490	2,682	0,004062257	1,096	3,968
Beauveria caledonica KCh J3.3	*Isaria tenuipes MU35*	1,531	0,231	6,633	0,435	2,627	0,00535902	1,096	3,830

Day 1 Compound 3 conversion statistics

ANOVA: Single Factor

DESCRIPTION					Alpha	0,05		
Group	*Count*	*Sum*	*Mean*	*Variance*	*SS*	*Std Err*	*Lower*	*Upper*
Isaria farinosa KCh KW1.1	3	5,87	1,96	0,505578	1,011155	0,490174	0,887942	3,023937
Beauveria bassiana KCh BBT	3	3,03	1,01	0,101763	0,203525	0,490174	-0,0582	2,077794
Metarhizium robertsii MU4	3	20,23	6,74	0,438746	0,877492	0,490174	5,674746	7,810742
Isaria fumosorosea KCh J2	3	1,4508	0,4836	0,014454	0,028909	0,490174	-0,5844	1,551598
Beauveria caledonica KCh J3.3	3	2,50	0,83	0,026245	0,052489	0,490174	-0,23398	1,902015
Isaria tenuipes MU35	3	14,19	4,73	3,238089	6,476179	0,490174	3,662038	5,798034

ANOVA

Sources	*SS*	*df*	*MS*	*F*	*P value*	*Eta-sq*	*RMSSE*	*Omega Sq*
Between Groups	96,71019	5	19,34204	26,83366	4,02E-06	0,917903	2,990745	0,877691
Within Groups	8,649749	12	0,720812					
Total	105,3599	17	6,197643					

Q TEST

group 1	group 2	mean	std err	q-stat	lower	upper	p-value	mean-crit	Cohen d
Isaria farinosa KCh KW1.1	*Beauveria bassiana KCh BBT*	0,946	0,490	1,930	-1,382	3,274	0,74580305	2,328	1,114
Isaria farinosa KCh KW1.1	*Metarhizium robertsii MU4*	4,787	0,490	9,766	2,458	7,115	0,000184518	2,328	5,638
Isaria farinosa KCh KW1.1	*Isaria fumosorosea KCh J2*	1,472	0,490	3,004	-0,856	3,801	0,337465772	2,328	1,734
Isaria farinosa KCh KW1.1	*Beauveria caledonica KCh J3.3*	1,122	0,490	2,289	-1,206	3,450	0,602832567	2,328	1,321
Isaria farinosa KCh KW1.1	*Isaria tenuipes MU35*	2,774	0,490	5,659	0,446	5,102	0,01690416	2,328	3,267
Beauveria bassiana KCh BBT	*Metarhizium robertsii MU4*	5,733	0,490	11,696	3,405	8,061	3,08489E-05	2,328	6,753
Beauveria bassiana KCh BBT	*Isaria fumosorosea KCh J2*	0,526	0,490	1,073	-1,802	2,855	0,969520553	2,328	0,620
Beauveria bassiana KCh BBT	*Beauveria caledonica KCh J3.3*	0,176	0,490	0,359	-2,153	2,504	0,999814855	2,328	0,207
Beauveria bassiana KCh BBT	*Isaria tenuipes MU35*	3,720	0,490	7,590	1,392	6,049	0,001803273	2,328	4,382
Metarhizium robertsii MU4	*Isaria fumosorosea KCh J2*	6,259	0,490	12,769	3,931	8,587	1,24344E-05	2,328	7,372
Metarhizium robertsii MU4	*Beauveria caledonica KCh J3.3*	5,909	0,490	12,054	3,580	8,237	2,26266E-05	2,328	6,960
Metarhizium robertsii MU4	*Isaria tenuipes MU35*	2,013	0,490	4,106	-0,316	4,341	0,105881442	2,328	2,371
Isaria fumosorosea KCh J2	*Beauveria caledonica KCh J3.3*	0,350	0,490	0,715	-1,978	2,679	0,994961758	2,328	0,413
Isaria fumosorosea KCh J2	*Isaria tenuipes MU35*	4,246	0,490	8,663	1,918	6,575	0,000565043	2,328	5,002
Beauveria caledonica KCh J3.3	*Isaria tenuipes MU35*	3,896	0,490	7,948	1,568	6,224	0,001214475	2,328	4,589

Day 3 Compound 3 conversion statistics

ANOVA: Single Factor

DESCRIPTION					Alpha	0,05		
Group	*Count*	*Sum*	*Mean*	*Variance*	*SS*	*Std Err*	*Lower*	*Upper*
Isaria farinosa KCh KW1.1	3	5,03	1,68	0,236264	0,472529	0,312656	0,994812	2,357249
Beauveria bassiana KCh BBT	3	3,94	1,31	0,236618	0,473236	0,312656	0,633122	1,995559
Metarhizium robertsii MU4	3	16,40	5,47	0,830967	1,661934	0,312656	4,785665	6,148102
Isaria fumosorosea KCh J2	3	0,78	0,26	0,015843	0,031686	0,312656	-0,42282	0,93962
Beauveria caledonica KCh J3.3	3	1,86	0,62	0,00575	0,011499	0,312656	-0,06012	1,302312
Isaria tenuipes MU35	3	7,80	2,60	0,434125	0,868249	0,312656	1,919366	3,281803

ANOVA								
Sources	*SS*	*df*	*MS*	*F*	*P value*	*Eta-sq*	*RMSSE*	*Omega Sq*
Between Groups	53,66688	5	10,73338	36,60008	7,34E-07	0,938462	3,492853	0,908163
Within Groups	3,519132	12	0,293261					
Total	57,18601	17	3,363883					

Q TEST

group 1	group 2	mean	std err	q-stat	lower	upper	p-value	mean-crit	Cohen d
Isaria farinosa KCh KW1.1	*Beauveria bassiana KCh BBT*	0,362	0,313	1,157	-1,123	1,847	0,958487299	1,485	0,668
Isaria farinosa KCh KW1.1	*Metarhizium robertsii MU4*	3,791	0,313	12,125	2,306	5,276	2,13083E-05	1,485	7,000
Isaria farinosa KCh KW1.1	*Isaria fumosorosea KCh J2*	1,418	0,313	4,534	-0,067	2,903	0,064509041	1,485	2,618
Isaria farinosa KCh KW1.1	*Beauveria caledonica KCh J3.3*	1,055	0,313	3,374	-0,430	2,540	0,234765202	1,485	1,948
Isaria farinosa KCh KW1.1	*Isaria tenuipes MU35*	0,925	0,313	2,957	-0,561	2,410	0,352325464	1,485	1,707
Beauveria bassiana KCh BBT	*Metarhizium robertsii MU4*	4,153	0,313	13,282	2,667	5,638	8,21976E-06	1,485	7,668
Beauveria bassiana KCh BBT	*Isaria fumosorosea KCh J2*	1,056	0,313	3,377	-0,429	2,541	0,233995362	1,485	1,950
Beauveria bassiana KCh BBT	*Beauveria caledonica KCh J3.3*	0,693	0,313	2,217	-0,792	2,178	0,63192227	1,485	1,280
Beauveria bassiana KCh BBT	*Isaria tenuipes MU35*	1,286	0,313	4,114	-0,199	2,771	0,104942334	1,485	2,375
Metarhizium robertsii MU4	*Isaria fumosorosea KCh J2*	5,208	0,313	16,659	3,723	6,694	7,06835E-07	1,485	9,618
Metarhizium robertsii MU4	*Beauveria caledonica KCh J3.3*	4,846	0,313	15,499	3,361	6,331	1,56229E-06	1,485	8,948
Metarhizium robertsii MU4	*Isaria tenuipes MU35*	2,866	0,313	9,168	1,381	4,351	0,000335502	1,485	5,293
Isaria fumosorosea KCh J2	*Beauveria caledonica KCh J3.3*	0,363	0,313	1,160	-1,122	1,848	0,958016743	1,485	0,670
Isaria fumosorosea KCh J2	*Isaria tenuipes MU35*	2,342	0,313	7,491	0,857	3,827	0,002012423	1,485	4,325
Beauveria caledonica KCh J3.3	*Isaria tenuipes MU35*	1,979	0,313	6,331	0,494	3,465	0,007624427	1,485	3,655

Day 7 Compound 3 conversion statistics

ANOVA: Single Factor

DESCRIPTION					Alpha	0,05		
Group	*Count*	*Sum*	*Mean*	*Variance*	*SS*	*Std Err*	*Lower*	*Upper*
Isaria farinosa KCh KW1.1	3	5,03	1,68	0,236264	0,472529	0,217297	1,202582	2,149479
Beauveria bassiana KCh BBT	3	4,95	1,65	0,253296	0,506592	0,217297	1,17721	2,124108
Metarhizium robertsii MU4	3	9,93	3,31	0,123347	0,246695	0,217297	2,837405	3,784303
Isaria fumosorosea KCh J2	3	0,72	0,24	0,005982	0,011964	0,217297	-0,23347	0,713428
Beauveria caledonica KCh J3.3	3	1,66	0,55	0,002939	0,005878	0,217297	0,078568	1,025466
Isaria tenuipes MU35	3	6,72	2,24	0,228093	0,456186	0,217297	1,765185	2,712083

ANOVA								
Sources	*SS*	*df*	*MS*	*F*	*P value*	*Eta-sq*	*RMSSE*	*Omega Sq*
Between Groups	18,87112	5	3,774224	26,64404	4,18E-06	0,917367	2,980159	0,876898
Within Groups	1,699843	12	0,141654					
Total	20,57096	17	1,210057					

Q TEST

group 1	group 2	mean	std err	q-stat	lower	upper	p-value	mean-crit	Cohen d
Isaria farinosa KCh KW1.1	*Beauveria bassiana KCh BBT*	0,025	0,217	0,117	-1,007	1,058	0,999999291	1,032	0,067
Isaria farinosa KCh KW1.1	*Metarhizium robertsii MU4*	1,635	0,217	7,523	0,603	2,667	0,001941281	1,032	4,344
Isaria farinosa KCh KW1.1	*Isaria fumosorosea KCh J2*	1,436	0,217	6,609	0,404	2,468	0,005511921	1,032	3,816
Isaria farinosa KCh KW1.1	*Beauveria caledonica KCh J3.3*	1,124	0,217	5,173	0,092	2,156	0,030237747	1,032	2,986
Isaria farinosa KCh KW1.1	*Isaria tenuipes MU35*	0,563	0,217	2,589	-0,470	1,595	0,483270938	1,032	1,495
Beauveria bassiana KCh BBT	*Metarhizium robertsii MU4*	1,660	0,217	7,640	0,628	2,692	0,001704683	1,032	4,411
Beauveria bassiana KCh BBT	*Isaria fumosorosea KCh J2*	1,411	0,217	6,492	0,379	2,443	0,006315704	1,032	3,748
Beauveria bassiana KCh BBT	*Beauveria caledonica KCh J3.3*	1,099	0,217	5,056	0,066	2,131	0,03476058	1,032	2,919
Beauveria bassiana KCh BBT	*Isaria tenuipes MU35*	0,588	0,217	2,706	-0,444	1,620	0,439335709	1,032	1,562
Metarhizium robertsii MU4	*Isaria fumosorosea KCh J2*	3,071	0,217	14,132	2,039	4,103	4,24344E-06	1,032	8,159
Metarhizium robertsii MU4	*Beauveria caledonica KCh J3.3*	2,759	0,217	12,696	1,727	3,791	1,32036E-05	1,032	7,330
Metarhizium robertsii MU4	*Isaria tenuipes MU35*	1,072	0,217	4,934	0,040	2,104	0,040180523	1,032	2,849
Isaria fumosorosea KCh J2	*Beauveria caledonica KCh J3.3*	0,312	0,217	1,436	-0,720	1,344	0,904020299	1,032	0,829
Isaria fumosorosea KCh J2	*Isaria tenuipes MU35*	1,999	0,217	9,198	0,966	3,031	0,000325344	1,032	5,310
Beauveria caledonica KCh J3.3	*Isaria tenuipes MU35*	1,687	0,217	7,762	0,654	2,719	0,001490135	1,032	4,481

Day 10 Compound 3 conversion statistics

ANOVA: Single Factor

DESCRIPTION					Alpha	0,05		
Group	*Count*	*Sum*	*Mean*	*Variance*	*SS*	*Std Err*	*Lower*	*Upper*
Isaria farinosa KCh KW1.1	3	0	0	0	0	2,795216	-5,99514	5,995142
Beauveria bassiana KCh BBT	3	121,99	40,66	59,781	119,562	2,795216	34,66685	46,65713
Metarhizium robertsii MU4	3	66,88	22,29	58,50743	117,0149	2,795216	16,29915	28,28944
Beauveria bassiana KCh J1.5	3	0	0	0	0	2,795216	-5,99514	5,995142
Isaria fumosorosea KCh J2	3	44,39	14,80	5,640105	11,28021	2,795216	8,801428	20,79171
Beauveria caledonica KCh J3.3	3	51,35	17,12	26,59502	53,19004	2,795216	11,12066	23,11095
Isaria tenuipes MU35	3	71,32	23,77	13,55433	27,10866	2,795216	17,77689	29,76718

ANOVA

Sources	*SS*	*df*	*MS*	*F*	*P value*	*Eta-sq*	*RMSSE*	*Omega Sq*
Between Groups	3649,889	6	608,3149	25,95234	8,06E-07	0,917508	2,941221	0,876987
Within Groups	328,1558	14	23,4397					
Total	3978,045	20	198,9023					

Q TEST

group 1	*group 2*	*mean*	*std err*	*q-stat*	*lower*	*upper*	*p-value*	*mean-crit*	*Cohen d*
Isaria farinosa KCh KW1.1	*Beauveria bassiana KCh BBT*	40,662	2,795	14,547	27,164	54,160	1,10937E-06	13,498	8,399
Isaria farinosa KCh KW1.1	*Metarhizium robertsii MU4*	22,294	2,795	7,976	8,796	35,792	0,000927617	13,498	4,605
Isaria farinosa KCh KW1.1	*Beauveria bassiana KCh J1.5*	0,000	2,795	0,000	-13,498	13,498	1	13,498	0,000
Isaria farinosa KCh KW1.1	*Isaria fumosorosea KCh J2*	14,797	2,795	5,294	1,298	28,295	0,027694825	13,498	3,056
Isaria farinosa KCh KW1.1	*Beauveria caledonica KCh J3.3*	17,116	2,795	6,123	3,618	30,614	0,009516038	13,498	3,535
Isaria farinosa KCh KW1.1	*Isaria tenuipes MU35*	23,772	2,795	8,505	10,274	37,270	0,000492127	13,498	4,910
Beauveria bassiana KCh BBT	*Metarhizium robertsii MU4*	18,368	2,795	6,571	4,870	31,866	0,005357669	13,498	3,794
Beauveria bassiana KCh BBT	*Beauveria bassiana KCh J1.5*	40,662	2,795	14,547	27,164	54,160	1,10937E-06	13,498	8,399
Beauveria bassiana KCh BBT	*Isaria fumosorosea KCh J2*	25,865	2,795	9,253	12,367	39,364	0,000206215	13,498	5,342
Beauveria bassiana KCh BBT	*Beauveria caledonica KCh J3.3*	23,546	2,795	8,424	10,048	37,044	0,000541627	13,498	4,863
Beauveria bassiana KCh BBT	*Isaria tenuipes MU35*	16,890	2,795	6,042	3,392	30,388	0,010559401	13,498	3,489
Metarhizium robertsii MU4	*Beauveria bassiana KCh J1.5*	22,294	2,795	7,976	8,796	35,792	0,000927617	13,498	4,605
Metarhizium robertsii MU4	*Isaria fumosorosea KCh J2*	7,498	2,795	2,682	-6,000	20,996	0,512666005	13,498	1,549
Metarhizium robertsii MU4	*Beauveria caledonica KCh J3.3*	5,178	2,795	1,853	-8,320	18,677	0,836670343	13,498	1,070
Metarhizium robertsii MU4	*Isaria tenuipes MU35*	1,478	2,795	0,529	-12,020	14,976	0,999698514	13,498	0,305
Beauveria bassiana KCh J1.5	*Isaria fumosorosea KCh J2*	14,797	2,795	5,294	1,298	28,295	0,027694825	13,498	3,056
Beauveria bassiana KCh J1.5	*Beauveria caledonica KCh J3.3*	17,116	2,795	6,123	3,618	30,614	0,009516038	13,498	3,535
Beauveria bassiana KCh J1.5	*Isaria tenuipes MU35*	23,772	2,795	8,505	10,274	37,270	0,000492127	13,498	4,910
Isaria fumosorosea KCh J2	*Beauveria caledonica KCh J3.3*	2,319	2,795	0,830	-11,179	15,817	0,996233811	13,498	0,479
Isaria fumosorosea KCh J2	*Isaria tenuipes MU35*	8,975	2,795	3,211	-4,523	22,474	0,321226186	13,498	1,854
Beauveria caledonica KCh J3.3	*Isaria tenuipes MU35*	6,656	2,795	2,381	-6,842	20,154	0,636186897	13,498	1,375

Day 1 Compound 4 conversion statistics

ANOVA: Single Factor

DESCRIPTION					Alpha	0,05		
Group	*Count*	*Sum*	*Mean*	*Variance*	*SS*	*Std Err*	*Lower*	*Upper*
Isaria farinosa KCh KW1.1	3	81,19	27,06	42,33158	84,66315	3,87179	18,75797	35,36629
Beauveria bassiana KCh BBT	3	198,50	66,17	15,61364	31,22729	3,87179	57,86207	74,4704
Metarhizium robertsii MU4	3	222,81	74,27	29,43482	58,86963	3,87179	65,96609	82,57442
Beauveria bassiana KCh J1.5	3	33,61	11,20	55,22644	110,4529	3,87179	2,899273	19,5076
Isaria fumosorosea KCh J2	3	175,84	58,61	112,6864	225,3728	3,87179	50,30973	66,91806
Beauveria caledonica KCh J3.3	3	229,26	76,42	28,63701	57,27403	3,87179	68,11743	84,72575
Isaria tenuipes MU35	3	202,00	67,33	30,876	61,75201	3,87179	59,03075	75,63908

ANOVA								
Sources	*SS*	*df*	*MS*	*F*	*P value*	*Eta-sq*	*RMSSE*	*Omega Sq*
Between Groups	11449,75	6	1908,291	42,43262	3,43E-08	0,947877	3,760878	0,922106
Within Groups	629,6118	14	44,97227					
Total	12079,36	20	603,968					

Q TEST

group 1	*group 2*	*mean*	*std err*	*q-stat*	*lower*	*upper*	*p-value*	*mean-crit*	*Cohen d*
Isaria farinosa KCh KW1.1	*Beauveria bassiana KCh BBT*	39,104	3,872	10,100	20,407	57,801	8,03905E-05	18,697	5,831
Isaria farinosa KCh KW1.1	*Metarhizium robertsii MU4*	47,208	3,872	12,193	28,511	65,905	9,38591E-06	18,697	7,040
Isaria farinosa KCh KW1.1	*Beauveria bassiana KCh J1.5*	15,859	3,872	4,096	-2,838	34,556	0,122603046	18,697	2,365
Isaria farinosa KCh KW1.1	*Isaria fumosorosea KCh J2*	31,552	3,872	8,149	12,855	50,249	0,000752286	18,697	4,705
Isaria farinosa KCh KW1.1	*Beauveria caledonica KCh J3.3*	49,359	3,872	12,748	30,663	68,056	5,52964E-06	18,697	7,360
Isaria farinosa KCh KW1.1	*Isaria tenuipes MU35*	40,273	3,872	10,402	21,576	58,970	5,80555E-05	18,697	6,005
Beauveria bassiana KCh BBT	*Metarhizium robertsii MU4*	8,104	3,872	2,093	-10,593	26,801	0,751587855	18,697	1,208
Beauveria bassiana KCh BBT	*Beauveria bassiana KCh J1.5*	54,963	3,872	14,196	36,266	73,660	1,49998E-06	18,697	8,196
Beauveria bassiana KCh BBT	*Isaria fumosorosea KCh J2*	7,552	3,872	1,951	-11,145	26,249	0,803736884	18,697	1,126
Beauveria bassiana KCh BBT	*Beauveria caledonica KCh J3.3*	10,255	3,872	2,649	-8,442	28,952	0,526209361	18,697	1,529
Beauveria bassiana KCh BBT	*Isaria tenuipes MU35*	1,169	3,872	0,302	-17,528	19,866	0,99998863	18,697	0,174
Metarhizium robertsii MU4	*Beauveria bassiana KCh J1.5*	63,067	3,872	16,289	44,370	81,764	2,68888E-07	18,697	9,404
Metarhizium robertsii MU4	*Isaria fumosorosea KCh J2*	15,656	3,872	4,044	-3,041	34,353	0,13036054	18,697	2,335
Metarhizium robertsii MU4	*Beauveria caledonica KCh J3.3*	2,151	3,872	0,556	-16,546	20,848	0,999598877	18,697	0,321
Metarhizium robertsii MU4	*Isaria tenuipes MU35*	6,935	3,872	1,791	-11,762	25,632	0,855873357	18,697	1,034
Beauveria bassiana KCh J1.5	*Isaria fumosorosea KCh J2*	47,410	3,872	12,245	28,714	66,107	8,92404E-06	18,697	7,070
Beauveria bassiana KCh J1.5	*Beauveria caledonica KCh J3.3*	65,218	3,872	16,844	46,521	83,915	1,75507E-07	18,697	9,725
Beauveria bassiana KCh J1.5	*Isaria tenuipes MU35*	56,131	3,872	14,498	37,435	74,828	1,15709E-06	18,697	8,370
Isaria fumosorosea KCh J2	*Beauveria caledonica KCh J3.3*	17,808	3,872	4,599	-0,889	36,505	0,06661874	18,697	2,655
Isaria fumosorosea KCh J2	*Isaria tenuipes MU35*	8,721	3,872	2,252	-9,976	27,418	0,688872013	18,697	1,300
Beauveria caledonica KCh J3.3	*Isaria tenuipes MU35*	9,087	3,872	2,347	-9,610	27,784	0,650354366	18,697	1,355

Day 3 Compound 4 conversion statistics

ANOVA: Single Factor

DESCRIPTION					Alpha	0,05		
Group	*Count*	*Sum*	*Mean*	*Variance*	*SS*	*Std Err*	*Lower*	*Upper*
Isaria farinosa KCh KW1.1	3	217,21	72,40	9,132429	18,26486	2,930142	66,11938	78,68844
Beauveria bassiana KCh BBT	3	207,42	69,14	10,83076	21,66151	2,930142	62,85463	75,42369
Metarhizium robertsii MU4	3	200,97	66,99	58,24323	116,4865	2,930142	60,70688	73,27594
Beauveria bassiana KCh J1.5	3	202,69	67,56	71,81589	143,6318	2,930142	61,27814	73,8472
Isaria fumosorosea KCh J2	3	267,33	89,11	12,68509	25,37017	2,930142	82,82581	95,39487
Beauveria caledonica KCh J3.3	3	250,71	83,57	13,84606	27,69212	2,930142	77,28632	89,85539
Isaria tenuipes MU35	3	201,95	67,32	3,746971	7,493942	2,930142	61,03106	73,60012

ANOVA								
Sources	*SS*	*df*	*MS*	*F*	*P value*	*Eta-sq*	*RMSSE*	*Omega Sq*
Between Groups	1442,462	6	240,4104	9,333715	0,000314	0,800007	1,76387	0,704235
Within Groups	360,6008	14	25,7572					
Total	1803,063	20	90,15316					

Q TEST

group 1	*group 2*	*mean*	*std err*	*q-stat*	*lower*	*upper*	*p-value*	*mean-crit*	*Cohen d*
Isaria farinosa KCh KW1.1	*Beauveria bassiana KCh BBT*	3,265	2,930	1,114	-10,885	17,414	0,982485	14,14966	0,643281
Isaria farinosa KCh KW1.1	*Metarhizium robertsii MU4*	5,413	2,930	1,847	-8,737	19,562	0,83842	14,14966	1,066471
Isaria farinosa KCh KW1.1	*Beauveria bassiana KCh J1.5*	4,841	2,930	1,652	-9,308	18,991	0,89484	14,14966	0,953911
Isaria farinosa KCh KW1.1	*Isaria fumosorosea KCh J2*	16,706	2,930	5,702	2,557	30,856	0,016386	14,14966	3,291806
Isaria farinosa KCh KW1.1	*Beauveria caledonica KCh J3.3*	11,167	2,930	3,811	-2,983	25,317	0,170325	14,14966	2,200315
Isaria farinosa KCh KW1.1	*Isaria tenuipes MU35*	5,088	2,930	1,737	-9,061	19,238	0,871981	14,14966	1,002594
Beauveria bassiana KCh BBT	*Metarhizium robertsii MU4*	2,148	2,930	0,733	-12,002	16,297	0,998089	14,14966	0,42319
Beauveria bassiana KCh BBT	*Beauveria bassiana KCh J1.5*	1,576	2,930	0,538	-12,573	15,726	0,999667	14,14966	0,31063
Beauveria bassiana KCh BBT	*Isaria fumosorosea KCh J2*	19,971	2,930	6,816	5,822	34,121	0,003924	14,14966	3,935087
Beauveria bassiana KCh BBT	*Beauveria caledonica KCh J3.3*	14,432	2,930	4,925	0,282	28,581	0,044279	14,14966	2,843596
Beauveria bassiana KCh BBT	*Isaria tenuipes MU35*	1,824	2,930	0,622	-12,326	15,973	0,999235	14,14966	0,359313
Metarhizium robertsii MU4	*Beauveria bassiana KCh J1.5*	0,571	2,930	0,195	-13,578	14,721	0,999999	14,14966	0,112561
Metarhizium robertsii MU4	*Isaria fumosorosea KCh J2*	22,119	2,930	7,549	7,969	36,269	0,001566	14,14966	4,358277
Metarhizium robertsii MU4	*Beauveria caledonica KCh J3.3*	16,579	2,930	5,658	2,430	30,729	0,017327	14,14966	3,266786
Metarhizium robertsii MU4	*Isaria tenuipes MU35*	0,324	2,930	0,111	-13,825	14,474	1	14,14966	0,063877
Beauveria bassiana KCh J1.5	*Isaria fumosorosea KCh J2*	21,548	2,930	7,354	7,398	35,697	0,001994	14,14966	4,245717
Beauveria bassiana KCh J1.5	*Beauveria caledonica KCh J3.3*	16,008	2,930	5,463	1,859	30,158	0,022271	14,14966	3,154226
Beauveria bassiana KCh J1.5	*Isaria tenuipes MU35*	0,247	2,930	0,084	-13,903	14,397	1	14,14966	0,048683
Isaria fumosorosea KCh J2	*Beauveria caledonica KCh J3.3*	5,539	2,930	1,891	-8,610	19,689	0,824252	14,14966	1,091491
Isaria fumosorosea KCh J2	*Isaria tenuipes MU35*	21,795	2,930	7,438	7,645	35,944	0,001796	14,14966	4,2944
Beauveria caledonica KCh J3.3	*Isaria tenuipes MU35*	16,255	2,930	5,548	2,106	30,405	0,019981	14,14966	3,202909

Day 7 Compound 4 conversion statistics

ANOVA: Single Factor

DESCRIPTION					Alpha	0,05		
Group	*Count*	*Sum*	*Mean*	*Variance*	*SS*	*Std Err*	*Lower*	*Upper*
Isaria farinosa KCh KW1.1	3	202,52	67,51	2,329988	4,659975	2,89026	61,30613	73,70412
Beauveria bassiana KCh BBT	3	199,60	66,53	3,16917	6,33834	2,89026	60,33592	72,73391
Metarhizium robertsii MU4	3	197,23	65,74	136,6985	273,3971	2,89026	59,54333	71,94131
Beauveria bassiana KCh J1.5	3	237,11	79,04	17,79168	35,58337	2,89026	72,83638	85,23436
Isaria fumosorosea KCh J2	3	259,32	86,44	3,91199	7,823979	2,89026	80,24143	92,63941
Beauveria caledonica KCh J3.3	3	260,40	86,80	9,424197	18,84839	2,89026	80,60075	92,99873
Isaria tenuipes MU35	3	196,61	65,54	2,100109	4,200218	2,89026	59,33619	71,73417

ANOVA

Sources	*SS*	*df*	*MS*	*F*	*P value*	*Eta-sq*	*RMSSE*	*Omega Sq*
Between Groups	1745,047	6	290,8411	11,60542	9,67E-05	0,832601	1,966843	0,751868
Within Groups	350,8513	14	25,06081					
Total	2095,898	20	104,7949					

Q TEST

group 1	group 2	mean	std err	q-stat	lower	upper	p-value	mean-crit	Cohen d
Isaria farinosa KCh KW1.1	*Beauveria bassiana KCh BBT*	0,970	2,890	0,336	-12,987	14,927	0,999979	13,957	0,194
Isaria farinosa KCh KW1.1	*Metarhizium robertsii MU4*	1,763	2,890	0,610	-12,194	15,720	0,999318	13,957	0,352
Isaria farinosa KCh KW1.1	*Beauveria bassiana KCh J1.5*	11,530	2,890	3,989	-2,427	25,487	0,138884	13,957	2,303
Isaria farinosa KCh KW1.1	*Isaria fumosorosea KCh J2*	18,935	2,890	6,551	4,978	32,892	0,005494	13,957	3,782
Isaria farinosa KCh KW1.1	*Beauveria caledonica KCh J3.3*	19,295	2,890	6,676	5,338	33,252	0,004688	13,957	3,854
Isaria farinosa KCh KW1.1	*Isaria tenuipes MU35*	1,970	2,890	0,682	-11,987	15,927	0,998725	13,957	0,394
Beauveria bassiana KCh BBT	*Metarhizium robertsii MU4*	0,793	2,890	0,274	-13,164	14,750	0,999994	13,957	0,158
Beauveria bassiana KCh BBT	*Beauveria bassiana KCh J1.5*	12,500	2,890	4,325	-1,457	26,458	0,093248	13,957	2,497
Beauveria bassiana KCh BBT	*Isaria fumosorosea KCh J2*	19,906	2,890	6,887	5,948	33,863	0,003585	13,957	3,976
Beauveria bassiana KCh BBT	*Beauveria caledonica KCh J3.3*	20,265	2,890	7,011	6,308	34,222	0,003063	13,957	4,048
Beauveria bassiana KCh BBT	*Isaria tenuipes MU35*	1,000	2,890	0,346	-12,957	14,957	0,999975	13,957	0,200
Metarhizium robertsii MU4	*Beauveria bassiana KCh J1.5*	13,293	2,890	4,599	-0,664	27,250	0,066626	13,957	2,655
Metarhizium robertsii MU4	*Isaria fumosorosea KCh J2*	20,698	2,890	7,161	6,741	34,655	0,002537	13,957	4,135
Metarhizium robertsii MU4	*Beauveria caledonica KCh J3.3*	21,057	2,890	7,286	7,100	35,014	0,002171	13,957	4,206
Metarhizium robertsii MU4	*Isaria tenuipes MU35*	0,207	2,890	0,072	-13,750	14,164	1	13,957	0,041
Beauveria bassiana KCh J1.5	*Isaria fumosorosea KCh J2*	7,405	2,890	2,562	-6,552	21,362	0,561555	13,957	1,479
Beauveria bassiana KCh J1.5	*Beauveria caledonica KCh J3.3*	7,764	2,890	2,686	-6,193	21,721	0,511041	13,957	1,551
Beauveria bassiana KCh J1.5	*Isaria tenuipes MU35*	13,500	2,890	4,671	-0,457	27,457	0,060946	13,957	2,697
Isaria fumosorosea KCh J2	*Beauveria caledonica KCh J3.3*	0,359	2,890	0,124	-13,598	14,316	1	13,957	0,072
Isaria fumosorosea KCh J2	*Isaria tenuipes MU35*	20,905	2,890	7,233	6,948	34,862	0,002319	13,957	4,176
Beauveria caledonica KCh J3.3	*Isaria tenuipes MU35*	21,265	2,890	7,357	7,307	35,222	0,001986	13,957	4,248

Day 10 Compound 4 conversion statistics

ANOVA: Single Factor

DESCRIPTION Alpha 0,05

Group	Count	Sum	Mean	Variance	SS	Std Err	Lower	Upper
Isaria farinosa KCh KW1.1	3	0	0	0	0	1,510689	-3,69652	3,696522
Beauveria bassiana KCh BBT	3	35,21	11,74	20,53962	41,07925	1,510689	8,039652	15,4327
Isaria tenuipes MU35	3	0	0	0	0	1,510689	-3,69652	3,696522

ANOVA

Sources	SS	df	MS	F	P value	Eta-sq	RMSSE	Omega Sq
Between Groups	275,4756	2	137,7378	20,11786	0,002185	0,87023	2,589586	0,809466
Within Groups	41,07925	6	6,846542					
Total	316,5548	8	39,56936					

Q TEST

group 1	group 2	mean	std err	q-stat	lower	upper	p-value	mean-crit	Cohen d
Isaria farinosa KCh KW1.1	*Beauveria bassiana KCh BBT*	11,736	1,511	7,769	5,181	18,291	0,003675	6,555	4,485
Isaria farinosa KCh KW1.1	*Isaria tenuipes MU35*	0,000	1,511	0,000	-6,555	6,555	1	6,555	0,000
Beauveria bassiana KCh BBT	*Isaria tenuipes MU35*	11,736	1,511	7,769	5,181	18,291	0,003675	6,555	4,485

Day 1 Compound 5 conversion statistics

ANOVA: Single Factor

DESCRIPTION					Alpha	0,05		
Group	*Count*	*Sum*	*Mean*	*Variance*	*SS*	*Std Err*	*Lower*	*Upper*
Isaria farinosa KCh KW1.1	3	4,04	1,35	0,067938	0,135876	1,522216	-2,37928	5,070181
Beauveria bassiana KCh BBT	3	39,59	13,20	17,54694	35,09389	1,522216	9,470498	16,91995
Isaria tenuipes MU35	3	17,71	5,90	3,23939	6,47878	1,522216	2,176982	9,626438

ANOVA

Sources	*SS*	*df*	*MS*	*F*	*P value*	*Eta-sq*	*RMSSE*	*Omega Sq*
Between Groups	214,372	2	107,186	15,41928	0,004321	0,837127	2,267104	0,762147
Within Groups	41,70854	6	6,951424					
Total	256,0805	8	32,01006					

Q TEST

group 1	*group 2*	*mean*	*std err*	*q-stat*	*lower*	*upper*	*p-value*	*mean-crit*	*Cohen d*
Isaria farinosa KCh KW1.1	*Beauveria bassiana KCh BBT*	11,850	1,522	7,785	5,245	18,455	0,003638	6,605	4,494
Isaria farinosa KCh KW1.1	*Isaria tenuipes MU35*	4,556	1,522	2,993	-2,049	11,161	0,166478	6,605	1,728
Beauveria bassiana KCh BBT	*Isaria tenuipes MU35*	7,294	1,522	4,791	0,689	13,898	0,033924	6,605	2,766

Day 3 Compound 5 conversion statistics

ANOVA: Single Factor

DESCRIPTION					Alpha	0,05		
Group	*Count*	*Sum*	*Mean*	*Variance*	*SS*	*Std Err*	*Lower*	*Upper*
Isaria farinosa KCh KW1.1	3	16,43	5,48	1,269793	2,539586	1,384127	2,090583	8,864258
Beauveria bassiana KCh BBT	3	35,76	11,92	10,17224	20,34448	1,384127	8,53161	15,30528
Isaria tenuipes MU35	3	24,23	8,08	5,80024	11,60048	1,384127	4,690567	11,46424

ANOVA

Sources	*SS*	*df*	*MS*	*F*	*P value*	*Eta-sq*	*RMSSE*	*Omega Sq*
Between Groups	63,00036	2	31,50018	5,480747	0,044265	0,646258	1,351634	0,498928
Within Groups	34,48455	6	5,747424					
Total	97,4849	8	12,18561					

Q TEST

group 1	*group 2*	*mean*	*std err*	*q-stat*	*lower*	*upper*	*p-value*	*mean-crit*	*Cohen d*
Isaria farinosa KCh KW1.1	*Beauveria bassiana KCh BBT*	6,441	1,384	4,653	0,435	12,447	0,038135	6,006	2,687
Isaria farinosa KCh KW1.1	*Isaria tenuipes MU35*	2,600	1,384	1,878	-3,406	8,606	0,431797	6,006	1,085
Beauveria bassiana KCh BBT	*Isaria tenuipes MU35*	3,841	1,384	2,775	-2,165	9,847	0,20235	6,006	1,602

Day 7 Compound 5 conversion statistics

ANOVA: Single Factor

DESCRIPTION					Alpha	0,05		
Group	*Count*	*Sum*	*Mean*	*Variance*	*SS*	*Std Err*	*Lower*	*Upper*
Isaria farinosa KCh KW1.1	3	15,37	5,12	0,513128	1,026255	1,213234	2,154831	8,092185
Beauveria bassiana KCh BBT	3	33,03	11,01	11,5739	23,1478	1,213234	8,041059	13,97841
Isaria tenuipes MU35	3	22,61	7,54	1,160405	2,320809	1,213234	4,567523	10,50488

ANOVA

Sources	*SS*	*df*	*MS*	*F*	*P value*	*Eta-sq*	*RMSSE*	*Omega Sq*
Between Groups	52,53421	2	26,26711	5,948422	0,037681	0,664745	1,408122	0,52373
Within Groups	26,49487	6	4,415811					
Total	79,02908	8	9,878635					

Q TEST

group 1	*group 2*	*mean*	*std err*	*q-stat*	*lower*	*upper*	*p-value*	*mean-crit*	*Cohen d*
Isaria farinosa KCh KW1.1	*Beauveria bassiana KCh BBT*	5,886	1,213	4,852	0,622	11,150	0,032243	5,264	2,801
Isaria farinosa KCh KW1.1	*Isaria tenuipes MU35*	2,413	1,213	1,989	-2,852	7,677	0,395838	5,264	1,148
Beauveria bassiana KCh BBT	*Isaria tenuipes MU35*	3,474	1,213	2,863	-1,791	8,738	0,187079	5,264	1,653

Day 10 Compound 5 conversion statistics

ANOVA: Single Factor

DESCRIPTION Alpha 0,05

Group	*Count*	*Sum*	*Mean*	*Variance*	*SS*	*Std Err*	*Lower*	*Upper*
Isaria farinosa KCh KW1.1	3	0	0	0	0	1,167287	-2,50358	2,503581
Beauveria bassiana KCh BBT	3	1,40	0,47	0,019631	0,039263	1,167287	-2,03548	2,971686
Metarhizium robertsii MU4	3	10,22	3,41	28,58717	57,17434	1,167287	0,904558	5,91172
Beauveria bassiana KCh J1.5	3	0	0	0	0	1,167287	-2,50358	2,503581
Isaria fumosorosea KCh J2	3	0	0	0	0	1,167287	-2,50358	2,503581
Beauveria caledonica KCh J3.3	3	0	0	0	0	1,167287	-2,50358	2,503581
Isaria tenuipes MU35	3	1,18	0,39	0,006916	0,013832	1,167287	-2,11167	2,89549

ANOVA

Sources	*SS*	*df*	*MS*	*F*	*P value*	*Eta-sq*	*RMSSE*	*Omega Sq*
Between Groups	28,15704	6	4,692839	1,148046	0,38599	0,329768	0,618613	0,040582
Within Groups	57,22744	14	4,087674					
Total	85,38448	20	4,269224					

Q TEST

group 1	group 2	mean	std err	q-stat	lower	upper	p-value	mean-crit	Cohen d
Isaria farinosa KCh KW1.1	*Beauveria bassiana KCh BBT*	0,468	1,167	0,401	-5,169	6,105	0,999939	5,637	0,232
Isaria farinosa KCh KW1.1	*Metarhizium robertsii MU4*	3,408	1,167	2,920	-2,229	9,045	0,420905	5,637	1,686
Isaria farinosa KCh KW1.1	*Beauveria bassiana KCh J1.5*	0,000	1,167	0,000	-5,637	5,637	1	5,637	0,000
Isaria farinosa KCh KW1.1	*Isaria fumosorosea KCh J2*	0,000	1,167	0,000	-5,637	5,637	1	5,637	0,000
Isaria farinosa KCh KW1.1	*Beauveria caledonica KCh J3.3*	0,000	1,167	0,000	-5,637	5,637	1	5,637	0,000
Isaria farinosa KCh KW1.1	*Isaria tenuipes MU35*	0,392	1,167	0,336	-5,245	6,029	0,999979	5,637	0,194
Beauveria bassiana KCh BBT	*Metarhizium robertsii MU4*	2,940	1,167	2,519	-2,697	8,577	0,57941	5,637	1,454
Beauveria bassiana KCh BBT	*Beauveria bassiana KCh J1.5*	0,468	1,167	0,401	-5,169	6,105	0,999939	5,637	0,232
Beauveria bassiana KCh BBT	*Isaria fumosorosea KCh J2*	0,468	1,167	0,401	-5,169	6,105	0,999939	5,637	0,232
Beauveria bassiana KCh BBT	*Beauveria caledonica KCh J3.3*	0,468	1,167	0,401	-5,169	6,105	0,999939	5,637	0,232
Beauveria bassiana KCh BBT	*Isaria tenuipes MU35*	0,076	1,167	0,065	-5,561	5,713	1	5,637	0,038
Metarhizium robertsii MU4	*Beauveria bassiana KCh J1.5*	3,408	1,167	2,920	-2,229	9,045	0,420905	5,637	1,686
Metarhizium robertsii MU4	*Isaria fumosorosea KCh J2*	3,408	1,167	2,920	-2,229	9,045	0,420905	5,637	1,686
Metarhizium robertsii MU4	*Beauveria caledonica KCh J3.3*	3,408	1,167	2,920	-2,229	9,045	0,420905	5,637	1,686
Metarhizium robertsii MU4	*Isaria tenuipes MU35*	3,016	1,167	2,584	-2,621	8,653	0,552577	5,637	1,492
Beauveria bassiana KCh J1.5	*Isaria fumosorosea KCh J2*	0,000	1,167	0,000	-5,637	5,637	1	5,637	0,000
Beauveria bassiana KCh J1.5	*Beauveria caledonica KCh J3.3*	0,000	1,167	0,000	-5,637	5,637	1	5,637	0,000
Beauveria bassiana KCh J1.5	*Isaria tenuipes MU35*	0,392	1,167	0,336	-5,245	6,029	0,999979	5,637	0,194
Isaria fumosorosea KCh J2	*Beauveria caledonica KCh J3.3*	0,000	1,167	0,000	-5,637	5,637	1	5,637	0,000
Isaria fumosorosea KCh J2	*Isaria tenuipes MU35*	0,392	1,167	0,336	-5,245	6,029	0,999979	5,637	0,194
Beauveria caledonica KCh J3.3	*Isaria tenuipes MU35*	0,392	1,167	0,336	-5,245	6,029	0,999979	5,637	0,194

Day 1 Compound 6 conversion statistics

ANOVA: Single Factor

DESCRIPTION					Alpha	0,05		
Group	*Count*	*Sum*	*Mean*	*Variance*	*SS*	*Std Err*	*Lower*	*Upper*
Isaria farinosa KCh KW1.1	3	1,16	0,39	0,023709	0,047419	0,876154	-1,49273	2,265598
Beauveria bassiana KCh BBT	3	5,87	1,96	0,1044	0,208801	0,876154	0,078277	3,836604
Metarhizium robertsii MU4	3	16,12	5,37	15,87705	31,7541	0,876154	3,493912	7,252239
Beauveria bassiana KCh J1.5	3	0	0	0	0	0,876154	-1,87916	1,879164
Isaria fumosorosea KCh J2	3	5,19	1,73	0,002097	0,004195	0,876154	-0,14873	3,609599
Beauveria caledonica KCh J3.3	3	10,45	3,48	0,02305	0,046101	0,876154	1,604767	5,363094
Isaria tenuipes MU35	3	6,48	2,16	0,090256	0,180512	0,876154	0,281295	4,039622

ANOVA								
Sources	*SS*	*df*	*MS*	*F*	*P value*	*Eta-sq*	*RMSSE*	*Omega Sq*
Between Groups	60,33966	6	10,05661	4,366861	0,010844	0,651751	1,206491	0,490306
Within Groups	32,24113	14	2,302938					
Total	92,58078	20	4,629039					

Q TEST

group 1	*group 2*	*mean*	*std err*	*q-stat*	*lower*	*upper*	*p-value*	*mean-crit*	*Cohen d*
Isaria farinosa KCh KW1.1	*Beauveria bassiana KCh BBT*	1,571	0,876	1,793	-2,660	5,802	0,85532	4,231	1,035
Isaria farinosa KCh KW1.1	*Metarhizium robertsii MU4*	4,987	0,876	5,692	0,756	9,218	0,0166	4,231	3,286
Isaria farinosa KCh KW1.1	*Beauveria bassiana KCh J1.5*	0,386	0,876	0,441	-3,845	4,617	0,999894	4,231	0,255
Isaria farinosa KCh KW1.1	*Isaria fumosorosea KCh J2*	1,344	0,876	1,534	-2,887	5,575	0,922677	4,231	0,886
Isaria farinosa KCh KW1.1	*Beauveria caledonica KCh J3.3*	3,097	0,876	3,535	-1,133	7,328	0,230548	4,231	2,041
Isaria farinosa KCh KW1.1	*Isaria tenuipes MU35*	1,774	0,876	2,025	-2,457	6,005	0,777149	4,231	1,169
Beauveria bassiana KCh BBT	*Metarhizium robertsii MU4*	3,416	0,876	3,898	-0,815	7,647	0,154227	4,231	2,251
Beauveria bassiana KCh BBT	*Beauveria bassiana KCh J1.5*	1,957	0,876	2,234	-2,274	6,188	0,696255	4,231	1,290
Beauveria bassiana KCh BBT	*Isaria fumosorosea KCh J2*	0,227	0,876	0,259	-4,004	4,458	0,999995	4,231	0,150
Beauveria bassiana KCh BBT	*Beauveria caledonica KCh J3.3*	1,526	0,876	1,742	-2,704	5,757	0,870343	4,231	1,006
Beauveria bassiana KCh BBT	*Isaria tenuipes MU35*	0,203	0,876	0,232	-4,028	4,434	0,999998	4,231	0,134
Metarhizium robertsii MU4	*Beauveria bassiana KCh J1.5*	5,373	0,876	6,133	1,142	9,604	0,009403	4,231	3,541
Metarhizium robertsii MU4	*Isaria fumosorosea KCh J2*	3,643	0,876	4,158	-0,588	7,874	0,113991	4,231	2,400
Metarhizium robertsii MU4	*Beauveria caledonica KCh J3.3*	1,889	0,876	2,156	-2,342	6,120	0,727206	4,231	1,245
Metarhizium robertsii MU4	*Isaria tenuipes MU35*	3,213	0,876	3,667	-1,018	7,444	0,199997	4,231	2,117
Beauveria bassiana KCh J1.5	*Isaria fumosorosea KCh J2*	1,730	0,876	1,975	-2,501	5,961	0,795127	4,231	1,140
Beauveria bassiana KCh J1.5	*Beauveria caledonica KCh J3.3*	3,484	0,876	3,976	-0,747	7,715	0,140987	4,231	2,296
Beauveria bassiana KCh J1.5	*Isaria tenuipes MU35*	2,160	0,876	2,466	-2,070	6,391	0,601242	4,231	1,424
Isaria fumosorosea KCh J2	*Beauveria caledonica KCh J3.3*	1,753	0,876	2,001	-2,477	5,984	0,785689	4,231	1,155
Isaria fumosorosea KCh J2	*Isaria tenuipes MU35*	0,430	0,876	0,491	-3,801	4,661	0,999804	4,231	0,283
Beauveria caledonica KCh J3.3	*Isaria tenuipes MU35*	1,323	0,876	1,511	-2,907	5,554	0,927597	4,231	0,872

Day 3 Compound 6 conversion statistics

ANOVA: Single Factor

DESCRIPTION					Alpha	0,05		
Group	*Count*	*Sum*	*Mean*	*Variance*	*SS*	*Std Err*	*Lower*	*Upper*
Isaria farinosa KCh KW1.1	3	14,86	4,95	0,132009	0,264019	1,389607	1,974239	7,935059
Beauveria bassiana KCh BBT	3	11,63	3,88	0,211088	0,422176	1,389607	0,895558	6,856378
Metarhizium robertsii MU4	3	39,73	13,24	17,26399	34,52797	1,389607	10,26433	16,22515
Beauveria bassiana KCh J1.5	3	9,16	3,05	2,055446	4,110892	1,389607	0,07447	6,03529
Isaria fumosorosea KCh J2	3	13,69	4,56	1,507563	3,015126	1,389607	1,581744	7,542564
Beauveria caledonica KCh J3.3	3	17,27	5,76	2,520906	5,041811	1,389607	2,776851	8,737671
Isaria tenuipes MU35	3	13,50	4,50	16,86014	33,72029	1,389607	1,519509	7,480329

ANOVA								
Sources	*SS*	*df*	*MS*	*F*	*P value*	*Eta-sq*	*RMSSE*	*Omega Sq*
Between Groups	211,6205	6	35,27009	6,088377	0,002594	0,722938	1,424591	0,592472
Within Groups	81,10228	14	5,79302					
Total	292,7228	20	14,63614					

Q TEST

group 1	group 2	mean	std err	q-stat	lower	upper	p-value	mean-crit	Cohen d
Isaria farinosa KCh KW1.1	*Beauveria bassiana KCh BBT*	1,079	1,390	0,776	-5,632	7,789	0,997379	6,710	0,448
Isaria farinosa KCh KW1.1	*Metarhizium robertsii MU4*	8,290	1,390	5,966	1,580	15,000	0,011656	6,710	3,444
Isaria farinosa KCh KW1.1	*Beauveria bassiana KCh J1.5*	1,900	1,390	1,367	-4,811	8,610	0,953438	6,710	0,789
Isaria farinosa KCh KW1.1	*Isaria fumosorosea KCh J2*	0,392	1,390	0,282	-6,318	7,103	0,999992	6,710	0,163
Isaria farinosa KCh KW1.1	*Beauveria caledonica KCh J3.3*	0,803	1,390	0,578	-5,908	7,513	0,9995	6,710	0,333
Isaria farinosa KCh KW1.1	*Isaria tenuipes MU35*	0,455	1,390	0,327	-6,256	7,165	0,999982	6,710	0,189
Beauveria bassiana KCh BBT	*Metarhizium robertsii MU4*	9,369	1,390	6,742	2,658	16,079	0,004309	6,710	3,893
Beauveria bassiana KCh BBT	*Beauveria bassiana KCh J1.5*	0,821	1,390	0,591	-5,889	7,531	0,99943	6,710	0,341
Beauveria bassiana KCh BBT	*Isaria fumosorosea KCh J2*	0,686	1,390	0,494	-6,024	7,397	0,999797	6,710	0,285
Beauveria bassiana KCh BBT	*Beauveria caledonica KCh J3.3*	1,881	1,390	1,354	-4,829	8,592	0,955469	6,710	0,782
Beauveria bassiana KCh BBT	*Isaria tenuipes MU35*	0,624	1,390	0,449	-6,086	7,334	0,999883	6,710	0,259
Metarhizium robertsii MU4	*Beauveria bassiana KCh J1.5*	10,190	1,390	7,333	3,479	16,900	0,002047	6,710	4,234
Metarhizium robertsii MU4	*Isaria fumosorosea KCh J2*	8,683	1,390	6,248	1,972	15,393	0,008103	6,710	3,607
Metarhizium robertsii MU4	*Beauveria caledonica KCh J3.3*	7,487	1,390	5,388	0,777	14,198	0,024527	6,710	3,111
Metarhizium robertsii MU4	*Isaria tenuipes MU35*	8,745	1,390	6,293	2,034	15,455	0,00765	6,710	3,633
Beauveria bassiana KCh J1.5	*Isaria fumosorosea KCh J2*	1,507	1,390	1,085	-5,203	8,218	0,984689	6,710	0,626
Beauveria bassiana KCh J1.5	*Beauveria caledonica KCh J3.3*	2,702	1,390	1,945	-4,008	9,413	0,805793	6,710	1,123
Beauveria bassiana KCh J1.5	*Isaria tenuipes MU35*	1,445	1,390	1,040	-5,265	8,155	0,987635	6,710	0,600
Isaria fumosorosea KCh J2	*Beauveria caledonica KCh J3.3*	1,195	1,390	0,860	-5,515	7,906	0,99543	6,710	0,497
Isaria fumosorosea KCh J2	*Isaria tenuipes MU35*	0,062	1,390	0,045	-6,648	6,773	1	6,710	0,026
Beauveria caledonica KCh J3.3	*Isaria tenuipes MU35*	1,257	1,390	0,905	-5,453	7,968	0,994009	6,710	0,522

Day 7 Compound 6 conversion statistics

ANOVA: Single Factor

DESCRIPTION					Alpha	0,05		
Group	*Count*	*Sum*	*Mean*	*Variance*	*SS*	*Std Err*	*Lower*	*Upper*
Isaria farinosa KCh KW1.1	3	45,05	15,02	2,619504	5,239007	1,962774	10,80565	19,22511
Beauveria bassiana KCh BBT	3	19,25	6,42	0,319364	0,638729	1,962774	2,205768	10,62523
Metarhizium robertsii MU4	3	54,08	18,03	65,62558	131,2512	1,962774	13,81572	22,23518
Beauveria bassiana KCh J1.5	3	26,23	8,74	3,168066	6,336133	1,962774	4,532133	12,9516
Isaria fumosorosea KCh J2	3	23,02	7,67	2,476648	4,953296	1,962774	3,462556	11,88202
Beauveria caledonica KCh J3.3	3	19,16	6,39	6,680536	13,36107	1,962774	2,17571	10,59517
Isaria tenuipes MU35	3	5,73	1,91	0,01245	0,024899	1,962774	-2,30135	6,118112

ANOVA								
Sources	*SS*	*df*	*MS*	*F*	*P value*	*Eta-sq*	*RMSSE*	*Omega Sq*
Between Groups	549,2579	6	91,54299	7,920691	0,00073	0,772447	1,624879	0,66413
Within Groups	161,8043	14	11,55745					
Total	711,0622	20	35,55311					

Q TEST

group 1	group 2	mean	std err	q-stat	lower	upper	p-value	mean-crit	Cohen d
Isaria farinosa KCh KW1.1	*Beauveria bassiana KCh BBT*	8,600	1,963	4,381	-0,878	18,078	0,087072	9,4782	2,5297
Isaria farinosa KCh KW1.1	*Metarhizium robertsii MU4*	3,010	1,963	1,534	-6,468	12,488	0,922763	9,4782	0,8854
Isaria farinosa KCh KW1.1	*Beauveria bassiana KCh J1.5*	6,274	1,963	3,196	-3,205	15,752	0,325876	9,4782	1,8454
Isaria farinosa KCh KW1.1	*Isaria fumosorosea KCh J2*	7,343	1,963	3,741	-2,135	16,821	0,184198	9,4782	2,1600
Isaria farinosa KCh KW1.1	*Beauveria caledonica KCh J3.3*	8,630	1,963	4,397	-0,848	18,108	0,085463	9,4782	2,5385
Isaria farinosa KCh KW1.1	*Isaria tenuipes MU35*	13,107	1,963	6,678	3,629	22,585	0,004676	9,4782	3,8554
Beauveria bassiana KCh BBT	*Metarhizium robertsii MU4*	11,610	1,963	5,915	2,132	21,088	0,012443	9,4782	3,4151
Beauveria bassiana KCh BBT	*Beauveria bassiana KCh J1.5*	2,326	1,963	1,185	-7,152	11,805	0,976249	9,4782	0,6843
Beauveria bassiana KCh BBT	*Isaria fumosorosea KCh J2*	1,257	1,963	0,640	-8,221	10,735	0,999102	9,4782	0,3697
Beauveria bassiana KCh BBT	*Beauveria caledonica KCh J3.3*	0,030	1,963	0,015	-9,448	9,508	1	9,4782	0,0088
Beauveria bassiana KCh BBT	*Isaria tenuipes MU35*	4,507	1,963	2,296	-4,971	13,985	0,671073	9,4782	1,3258
Metarhizium robertsii MU4	*Beauveria bassiana KCh J1.5*	9,284	1,963	4,730	-0,195	18,762	0,056624	9,4782	2,7308
Metarhizium robertsii MU4	*Isaria fumosorosea KCh J2*	10,353	1,963	5,275	0,875	19,831	0,028369	9,4782	3,0454
Metarhizium robertsii MU4	*Beauveria caledonica KCh J3.3*	11,640	1,963	5,930	2,162	21,118	0,0122	9,4782	3,4239
Metarhizium robertsii MU4	*Isaria tenuipes MU35*	16,117	1,963	8,211	6,639	25,595	0,000698	9,4782	4,7408
Beauveria bassiana KCh J1.5	*Isaria fumosorosea KCh J2*	1,070	1,963	0,545	-8,409	10,548	0,999641	9,4782	0,3146
Beauveria bassiana KCh J1.5	*Beauveria caledonica KCh J3.3*	2,356	1,963	1,201	-7,122	11,835	0,974722	9,4782	0,6931
Beauveria bassiana KCh J1.5	*Isaria tenuipes MU35*	6,833	1,963	3,482	-2,645	16,312	0,244067	9,4782	2,0101
Isaria fumosorosea KCh J2	*Beauveria caledonica KCh J3.3*	1,287	1,963	0,656	-8,191	10,765	0,998974	9,4782	0,3785
Isaria fumosorosea KCh J2	*Isaria tenuipes MU35*	5,764	1,963	2,937	-3,714	15,242	0,414691	9,4782	1,6955
Beauveria caledonica KCh J3.3	*Isaria tenuipes MU35*	4,477	1,963	2,281	-5,001	13,955	0,677308	9,4782	1,3169

Day 10 Compound 6 conversion statistics
